# A new strategy for hit generation: Novel *in cellulo* active inhibitors of CYP121A1 from *Mycobacterium tuberculosis* via a combined X-ray crystallographic and phenotypic screening approach (XP screen)

**DOI:** 10.1016/j.ejmech.2022.114105

**Published:** 2022-02-15

**Authors:** Martyn Frederickson, Irwin R. Selvam, Dimitrios Evangelopoulos, Kirsty J. McLean, Mona M. Katariya, Richard B. Tunnicliffe, Bethany Campbell, Madeline E. Kavanagh, Sitthivut Charoensutthivarakul, Richard T. Blankley, Colin W. Levy, Luiz Pedro S. de Carvalho, David Leys, Andrew W. Munro, Anthony G. Coyne, Chris Abell

**Affiliations:** a Yusuf Hamied Department of Chemistry, University of Cambridge, Lensfield Road, Cambridge, CB2 1EW, United Kingdom; b Department of Chemistry, Manchester Institute of Biotechnology, University of Manchester, 131 Princess Street, Manchester, M1 7DN, United Kingdom; c Mycobacterium Metabolism and Antibiotic Research Laboratory, Francis Crick Institute, 1 Midland Road, London, NW1 1AT, United Kingdom; d Manchester Protein Structure Facility (MPSF), Manchester Institute of Biotechnology, University of Manchester, Manchester, M1 7DN, United Kingdom; e Agilent Technologies U.K. Ltd, 5500 Lakeside, Cheadle Royal, Cheshire, SK8 3GR, United Kingdom

**Keywords:** CYP121, Mycobacterium tuberculosis, X-ray crystallography, Drug discovery, Tuberculosis

## Abstract

There is a pressing need for new drugs against tuberculosis (TB) to combat the growing resistance to current antituberculars. Herein a novel strategy is described for hit generation against promising TB targets involving X-ray crystallographic screening in combination with phenotypic screening. This combined approach (XP Screen) affords both a validation of target engagement as well as determination of *in cellulo* activity. The utility of this method is illustrated by way of an XP Screen against CYP121A1, a cytochrome P450 enzyme from *Mycobacterium tuberculosis* (*Mtb*) championed as a validated drug discovery target. A focused screening set was synthesized and tested by such means, with several members of the set showing promising activity against *Mtb* strain H37Rv. One compound was observed as an X-ray hit against CYP121A1 and showed improved activity against *Mtb* strain H37Rv under multiple assay conditions (pan-assay activity). Data obtained during X-ray crystallographic screening were utilized in a structure-based campaign to design a limited number of analogues (less than twenty), many of which also showed pan-assay activity against *Mtb* strain H37Rv. These included the benzo[*b*][1,4]oxazine derivative (MIC_90_ 6.25 μM), a novel hit compound suitable as a starting point for a more involved hit to lead candidate medicinal chemistry campaign.

## Highlights

• CYP121 from *M.tuberculosis* has been previously shown to be a crucial target for the survival of the mycobacteria.
• Strategies previously employed have identified high affinity inhibitors however these have lacked activity on *M.tuberculosis.*
• The strategy reported here uses a combination of X-ray crystallography and phenotypic screening (XP Screen) to identify compounds.
• The XP screen approach identified a number of compounds which show good affinity (up to 3.2 μM) and MIC against *M.tuberculosis* (up to 6.25 μM).

## Graphical abstract

**Figure:**
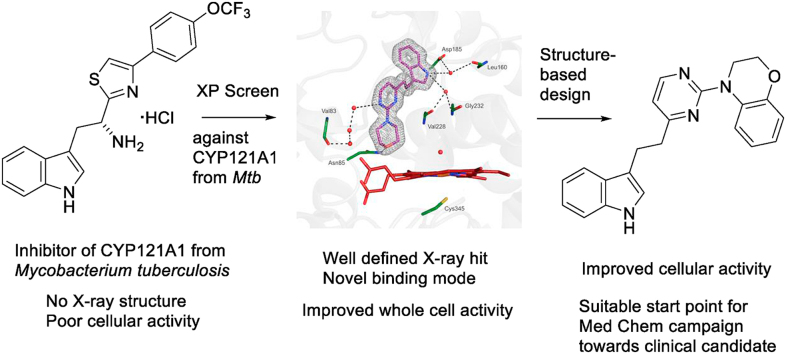

## 1 Introduction

The pathogenic bacterium *Mycobacterium tuberculosis* (*Mtb*) is the causative agent of tuberculosis (TB) for which over 10 million people globally were diagnosed and treated in 2019 and which was responsible for over 1.4 million deaths [1]. Antibiotic resistant strains of *Mtb* constitute a major worldwide healthcare problem, ensuring the continued demand for the development of new anti-TB drugs, particularly those that work *via* novel mechanisms. The *Mtb* genome encodes twenty cytochrome P450 enzymes (CYPs), an unusually large number given its relatively small size (4.41 Mb pairs), representing a CYP gene density over 240-fold greater than the human genome [2,3]. The preponderance of P450s in the *Mtb* genome, indicating a heavy reliance by the bacterium (which primarily exploits highly perfused tissues) on this class of mono-oxygenase enzymes, has resulted in *Mtb* CYPs being highlighted as viable targets for new anti-TB therapeutics.

**Fig. 1:**
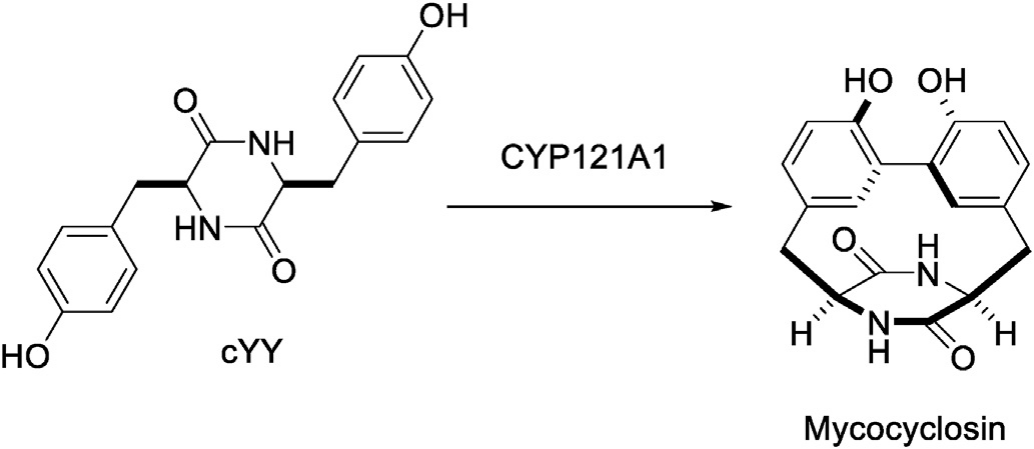
Synthesis of mycocyclosin from cyclo(l-tyrosyl-l-tyrosyl) (cYY) by CYP121A1.

CYP121A1 (mycocyclosin synthase; EC 1.14.19.70; Rv2276), functioning downstream of the cyclo(l-tyrosyl-l-tyrosyl) (cYY) synthase (EC 2.3.2.21; Rv2275), catalyzes the conversion in *Mtb* of the cyclic dipeptide cYY into the highly strained biaryl-containing natural product mycocyclosin (Fig. 1) [[4], [5], [6]]. Whilst the precise function of mycocyclosin remains unknown, ablation of CYP121A1 expression *via* genetic knockouts showed the enzyme to be essential for the viability of the bacterium *in vitro*; CYP121A1 is thus seen as an important target for anti-TB drug research [7]. Herein the use of a combination of X-ray crystallographic screening against CYP121A1 and phenotypic screening of whole-cell *Mtb* is described as a novel approach in hit discovery (XP Screen). This approach is used to highlight a series of compounds with molecular properties suitable for further elaboration into more potent anti-TB agents.

**Fig. 2:**
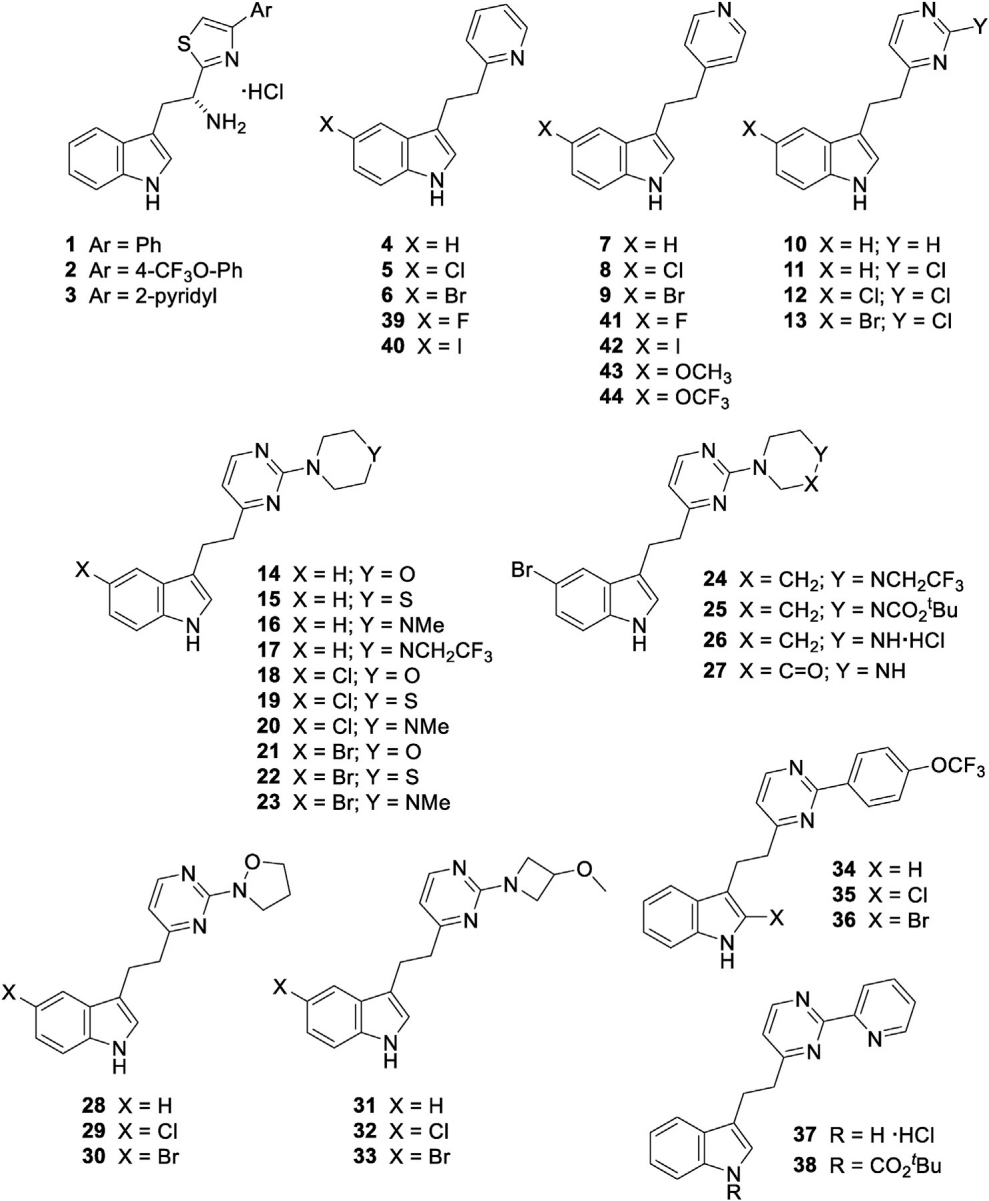
Initial hits (**1**–**3**), structural screening set (**4**–**37**) and follow-up analogues (**39**–**44**).

As part of continuing efforts aimed at the discovery of novel inhibitors of CYP121A1 [[8], [9], [10]], the binding mode of a novel class of inhibitors with moderate activity against *Mtb* strain H37Rv was reinvestigated. Previous work had shown that a series of d-tryptophan derived thiazoles exhibited moderate potency (*K*_d_ ∼ 30–55 μM) against CYP121A1 in a UV–Vis spectrophotometric assay [10]. Some members of the series (Fig. 2, compounds **1**–**3**) also showed moderate activities (MIC_90_ ∼ 40–100 μM) against *Mtb* in whole-cell assays. Unfortunately, X-ray crystallographic structures of CYP121A1 in complex with compounds of this series could not be generated, which hampered our understanding of their binding modes and the rational design of more potent derivatives.

With these factors in mind a more thorough X-ray crystallographic screen of compounds akin to **1**–**3** was performed, to discover novel and simplified analogues with binding modes that were well understood, and were suitable starting points for a structure-based design campaign. The aim was also to couple this approach to phenotypic screening against *Mtb*, in order to ensure that the previously noted *in cellulo* activity against the bacterium was maintained, despite the chemical changes being made to the series. CYP121A1 in complex with a wide range of fragments and inhibitors has been previously reported [8,9]. The CYP121A1 binding pocket has been shown by X-ray crystallography to accommodate ligands in three distinct regions (i) binding to the heme group, (ii) binding in the cYY region and (iii) binding at the top of the binding pocket. All the compounds shown binding in these regions have been observed using high resolution crystal structures and have been characterised using biophysical techniques such as DSF and ITC.

## 2 Results and discussion

A focused screening set of compounds was synthesized (Fig. 2, compounds **4**–**37**) containing functionalities common to a number of the previous key compounds [10]. The structures were simplified by removing the chiral amino functionality, as previous studies had shown the precise stereochemistry to be unimportant in terms of affinity. In addition, the thiazole ring was replaced with a pyridine (**4** and **7**) and pyrimidine (**10**) rings, both in order to improve solubility and to allow the more rapid generation of diverse analogues. Halogens were added at key positions within these compounds (**5**–**6**, **8**–**9** and **11**–**13**) in order to potentially aid crystallographic interpretation of ligand binding modes; a similar concept (FragLites) has been reported recently [11] in the area of fragment-based discovery. The aim was also to reduce compound lipophilicity whilst retaining (as much as possible) overall compound topology. This was achieved by replacing unsaturated aromatic sidechains with achiral saturated ring systems containing solubilizing or other heavier ‘X-ray friendly’ heteroatoms (such as sulfur) (**14**–**33**). A small number of comparable direct aromatic analogues (**34**–**37**) were also included.

**Scheme 1:**
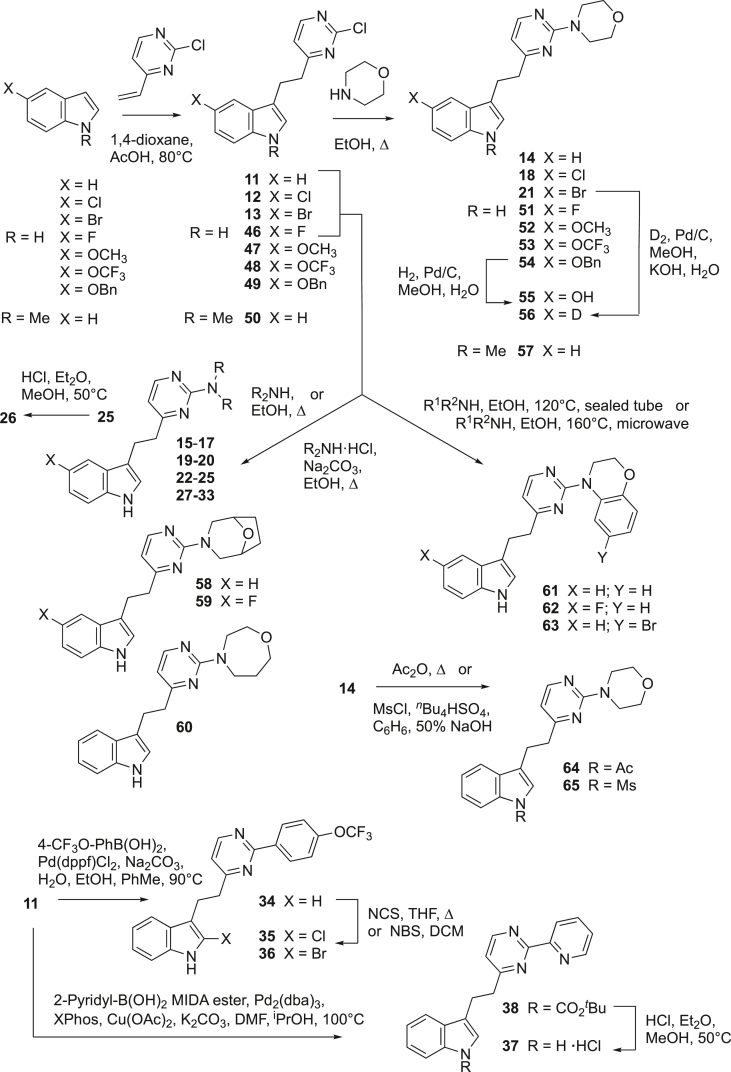
Synthesis of compounds **11**–**65**.

Compounds **4**–**9** were prepared by heating the appropriately substituted indole with either 2-vinylpyridine (for **4**–**6**) or 4-vinylpyridine (for **7**–**9**) in acetic acid as described previously [[12], [13], [14], [15]]. Similar reactions [13] between 2-chloro-4-vinylpyrimidine [16] in a mixture of acetic acid and 1,4-dioxane afforded **11**–**13**. Hydrogenation of **11** over palladium on carbon in ethanol in the presence of triethylamine yielded the known pyrimidine **10** [13]. Treatment of **11**–**13** with the appropriate secondary amine (or amine hydrochloride salt) in hot ethanol (with added anhydrous sodium carbonate when using an amine hydrochloride) gave **14**–**25** and **27**–**33** (Scheme 1). Heating *tert*-butyl carbamate **25** with hydrogen chloride in diethyl ether and methanol afforded the amine hydrochloride **26**. Treatment of chloride **11** with 4-trifluoromethoxyphenylboronic acid and catalytic palladium(0) afforded biaryl derivative **34**. Chlorination of **34** with *N*-chlorosuccinimide in hot tetrahydrofuran gave **35**. Bromination of **34** with *N*-bromosuccinimide to prepare **36** was more facile and proceeded at room temperature in dichloromethane. Palladium(0) catalyzed arylation of **11** with 2-pyridylboronic acid *N*-methyliminodiacetic acid (MIDA) ester according to published conditions [17] afforded the 2-pyridyl derivative **37** (isolated as the hydrochloride salt after purification *via* formation of the *tert*-butyl carbamate **38** and subsequent acid catalyzed deprotection).

**Fig. 3:**
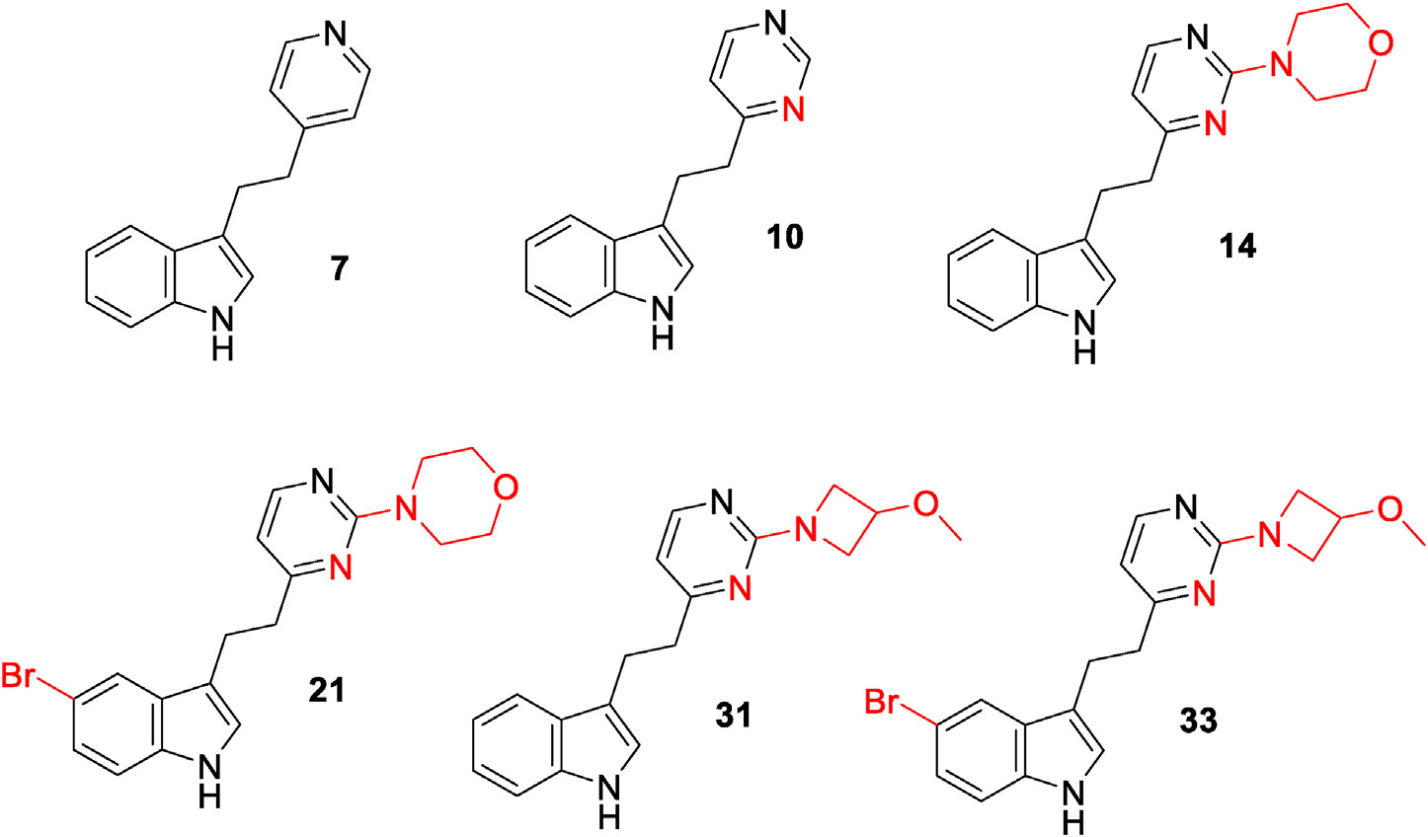
Hits from X-ray crystallographic screening (variances from the core scaffold **7** are highlighted in red).

Compounds **4**–**37** were screened crystallographically against CYP121A1 using soaking experiments. A number of hits (**7**, **10**, **14**, **21**, **31** and **33**) were identified, although the completeness of the electron density for the ligands was variable. All six of the X-ray crystallographic hits were ligands containing **7** as an integral subunit of molecular structure (Fig. 3). 4-Pyridyl derivative **7** has previously been used successfully [18] as a starting point for a structure-based design campaign against p38α MAP kinase, leading to inhibitors with low nanomolar affinity against the enzyme. It was noted that only certain substituents located at either end of the core structure (**7**) had proven successful crystallographically. In particular, substitution at C-2 of the pyrimidine ring appeared to favor both the morpholine and the constitutionally isomeric 3-methoxyazetidine groups. Three structures (with **7**, **31** and **33**) contained either incomplete density into which to place the ligand (indicating high mobility), or density suggestive that the ligand might bind in a variety of possible orientations; these three were therefore discarded as unsuitable as starting points from which to generate more potent compounds (although data obtained from them was utilized for guidance in subsequent structure-based design efforts).

**Fig. 4:**
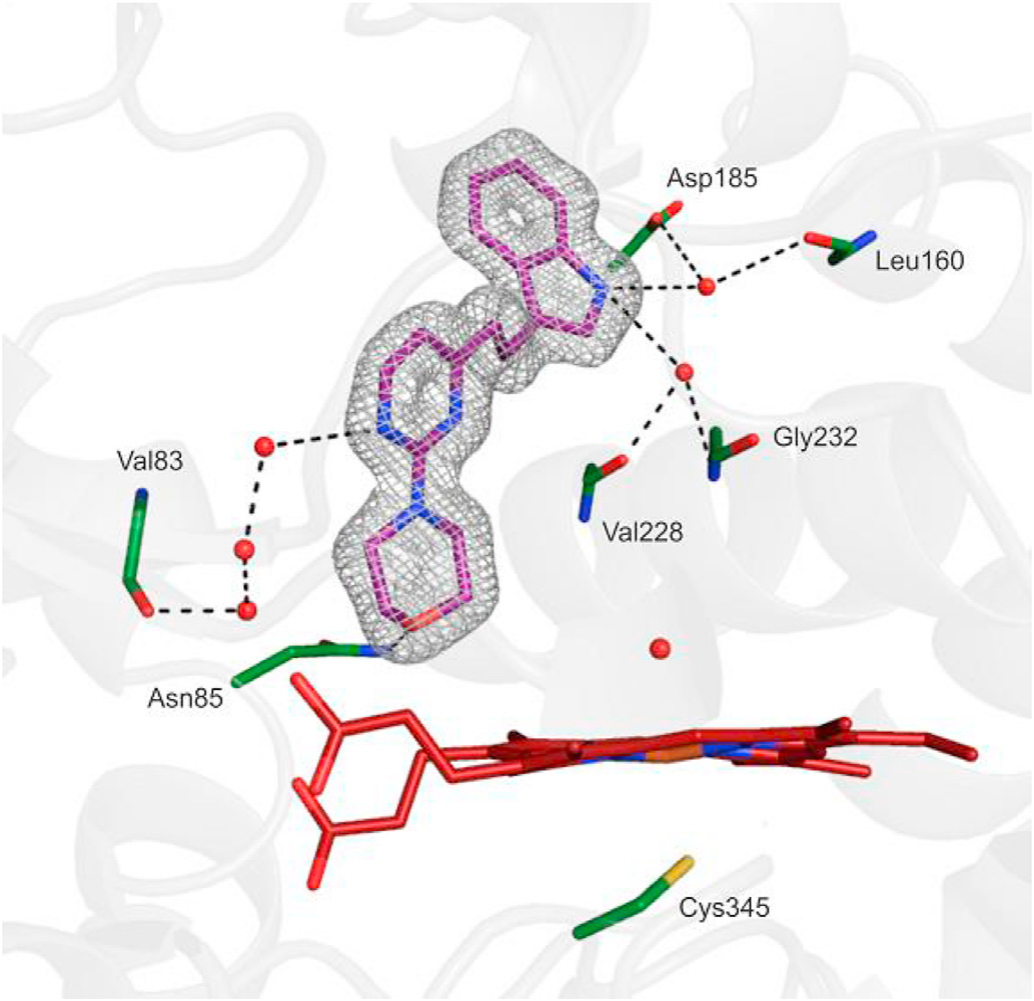
Morpholine **14** (in purple) fitted to the 2F_o_-F_c_ map (shown as a fine mesh contoured to one sigma). Possible hydrogen bonding network is shown as black dashes, all distances <3.2 Å. Protein residues shown in dark green and heme prosthetic group in red. Water molecules are shown as red spheres (pdb code: 7NQN).

X-ray crystal structures of compounds **10** and **21** with CYP121A1, whilst of better quality, still suffered from incomplete ligand electron density, either lacking complete density for the indole moiety (for **10**) or lacking density for the central linker (for **21**) (Fig. S1). In sharp contrast, the X-ray crystallographic structure for **14** included complete contiguous difference density into which the ligand could be fitted clearly and unequivocally (Fig. 4). Whilst morpholines were shown to bind to the enzyme, a number of morpholine replacements, varying by as little as a single heavy atom (piperazine and thiomorpholine), or with only one additional or deleted heavy atom (*N*-methylpiperazine and isoxazolidine), were all crystallographically ineffective. Similarly, none of the 2-aryl analogues **34**–**37** were successful structurally in this screen. This approach is thus an effective method by which to utilize the inherent subtlety of molecular binding events to effectively triage a range of extremely similar compounds within a moderately sized screening set.

Concurrent with the X-ray crystallographic screen, a limited number of the compounds (**15**, **23**, **30**, **32** and **34**) were also screened against whole-cell *Mtb* strain H37Rv in a panel of three different growth media, including one containing cholesterol as the sole carbon source (in an attempt to recapitulate more accurately the *in vivo* environment). Four of these compounds (**15**, **23**, **32** and **34**) showed promising *in cellulo* activity (MIC_90_ ∼12.5–50 μM) against the bacterium in one or more of the growth media tested (Table 1). The combined data suggested that it might be reasonably expect to find whole-cell activity in a variety of close analogues within the series, and that there was a possibility of generating high quality X-ray crystal hits during subsequent rounds of compound optimization.

**Table 1:** Whole-cell data against *Mtb* H37Rv. a) c7H9: Middlebrook 7H9 broth (Difco) supplemented with 10% (v/v) of Albumin, Dextrose, and Catalase (ADC) enrichment , 0.05% (v/v) tyloxapol and 0.02% (v/v) glycerol. b) 7H9-Low BSA: Middlebrook 7H9 broth with low BSA supplemented with 10% (v/v) of Albumin, Dextrose, and NaCl enrichment (ADN), 0.05% (v/v) tyloxapol and 0.02% (v/v) glycerol. This medium contains 0.05% instead of 0.5% (w/v) Bovine Albumin (Fraction V). c) MMM-Ch: Mycobacterial Minimal Medium with Cholesterol: 0.5 g/L l-asparagine, 1 g/L KH_2_PO_4_, 2.5 g/L Na_2_HPO_4_, 50 mg/L ferric ammonium citrate, 0.5 g/L MgSO_4_7H_2_O, 0.5 mg/L CaCl_2_, 0.1 mg/mL ZnSO_4_, 0.2% (v/v) tyloxapol, 0.2% (v/v) ethanol and 0.01% (v/v) cholesterol.

MIC_90_ (μM)			
Compound	c7H9^a^	7H9-Low BSA^b^	MMM-Ch^c^
**14**	25	25	12.5
**15**	200	100	25
**23**	100	25	12.5
**30**	200	200	100
**31**	50	25	25
**32**	200	100	50
**34**	100	100	12.5
**51**	25	50	25
**53**	25	25	50
**57**	50	25	25
**58**	200	50	25
**60**	25	50	25
**61**	12.5	25	6.25
**63**	25	50	50
**64**	>400	>400	>400
**Rifampicin (n** = **3)**	0.023	0.013	0.013

Compounds **4**–**37** were screened for their affinity against CYP121A1 using UV–Visible spectroscopy (UV–Vis) which involves monitoring the shift of the Soret band at 416.5 nm. Only the three 4-pyridyl compounds **7**–**9** showed any discernible effect, with all other derivatives showing no Soret band shift (Table 2). The lack of shift for **14** was expected given the fact that the compound had been shown to bind in a region of the protein that is distal to the heme. Presumably **7**–**9** interact with the heme iron *via* the pyridyl nitrogen atom, whilst steric encumbrance of the corresponding 2-pyridyl isomers **4**–**6** prevents them from binding in a similar fashion. Chloride **8** and bromide **9** show good levels of affinity (*K*_d_: 5.8 μM and 3.8 μM respectively) whereas the core structure **7** showed much lower potency (*K*_d_: 480 μM). The relatively moderate potency for **7** might explain our inability to obtain high quality X-ray structural data for this key compound, given its moderate lipophilicity (cLogP 2.12).

**Table 2:** UV–Visible spectrophotometric data. Compounds were screened at a concentration of 300 μM. All compounds listed gave Type II shift in the Soret band.

Compound	Structure	$K_{d}$ (μM)	Compound	Structure	$K_{d}$ (μM)
**7**		483 ± 76	**42**		3.2 ± 0.3
**8**		5.8 ± 0.7	**43**		105 ± 10
**9**		3.8 ± 0.5	**44**		12.7 ± 2.3
**41**		27.3 ± 4.8			

**Fig. 5:**
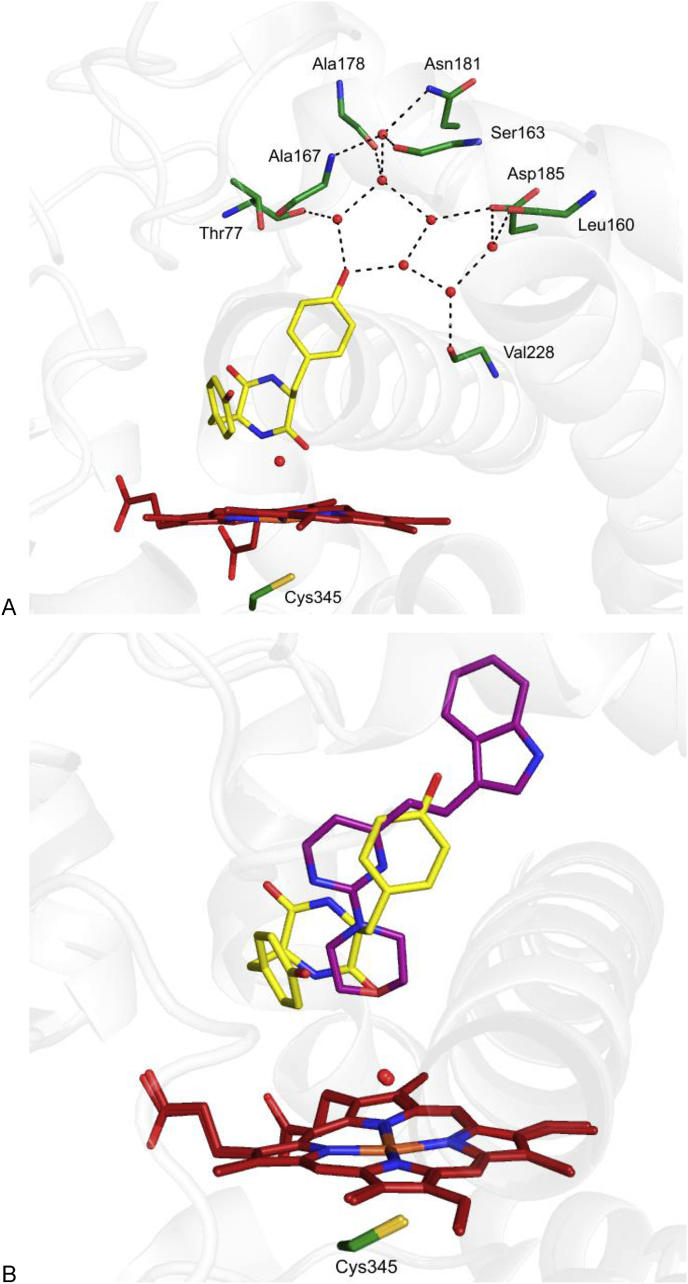
Differences between the binding modes of cYY and **14**. (a) Structure of cYY bound to CYP121A1 (PDB accession code: 3G5H). Waters in one of the two highly solvated pockets are shown as red spheres. A possible hydrogen bonding network is shown as black dashes, all distances are <3.2 Å. (b) Overlay of cYY and **14**-bound structures showing the position of the indolyl moiety in the highly solvated pocket. Water molecules are shown as red spheres.

The morpholine containing compound **14** is a ligand with a relatively low molecular mass and low cLogP (308 and 2.32 respectively) and as such represents a convenient starting point for elaboration. X-ray crystallography revealed that **14** binds to CYP121A1 with a number of interesting key features, chief amongst them being the fact that **14** does not engage with the iron (III) atom of the heme group, either directly or indirectly *via* a distally bound water molecule above the iron center. The X-ray structure of **14** indicated the presence of a hydrogen bond between the donor sidechain CONH_2_ of Asn85 and the morpholine oxygen atom, which acts as the acceptor. This hydrogen bond is observed in the cYY-bound structure (PDB accession code: 3G5H) [4], where the acceptor is instead one of the carbonyl oxygen atoms of the cyclic diketopiperizine core of cYY. In addition, cYY binds surrounded by two extensive water clusters [4] (Fig. 5a; water molecules in one of the two clusters are highlighted as red spheres). Overlaying of the structures containing cYY and **14** (Fig. 5b) shows that, upon binding, the indole moiety of **14** disrupts this hydrogen bonded network and ousts a large number of water molecules from this highly solvated pocket.

Numerous crystal structures of CYP121A1 have been deposited in the Protein Data Bank (PDB) containing ligands binding in differing locations within the enzyme active site. Morpholine **14** binds to CYP121A1 with a novel binding mode, in which the indolyl moiety is located high up in an otherwise highly solvated pocket that lies distal to the heme group. This novelty, and the fact that this pocket is a specific feature of CYP121A1, which might confer selectivity over other CYPs for compounds that bind at this site, cumulatively made it an extremely attractive start point for further development. Gratifyingly, **14** also showed appreciable *in cellulo* activity (MIC_90_ ∼12.5–25 μM) against *Mtb* in all three of the growth media tested, including complete 7H9, a medium highly enriched in Bovine Serum Albumin (BSA). This pan-assay activity welcomed, as it indicated that derivatives akin to our key X-ray hit might be expected to show relatively low levels of plasma protein binding (PPB), reinforcing that **14** would be a suitable starting point for further elaboration.

In order to more fully explore activity around core structure **7**, and to attempt to collect further structural data, a limited number of derivatives were synthesized (**41**–**44**). Identically substituted derivatives of the isomeric 2-pyridyl structure **4** were also prepared (**39**–**40**) by analogous means. Only the 4-pyridyl derivatives (**41**–**44**) showed any activity, with fluoride **41** [19], iodide **42** and trifluoromethyl ether **44** showing the greatest affinities (*K*_d_: 27.3 μM, 3.2 μM and 12.7 μM respectively) whereas methyl ether **43** was less active (*K*_d_: 105 μM), although still significantly more potent than **7**. Unfortunately, useable structural data for **39**–**44** could not be obtained (**44** gave a partial structure), and so further work on both the 4-pyridyl and 2-pyridyl compounds was discontinued.

**Fig. 6:**
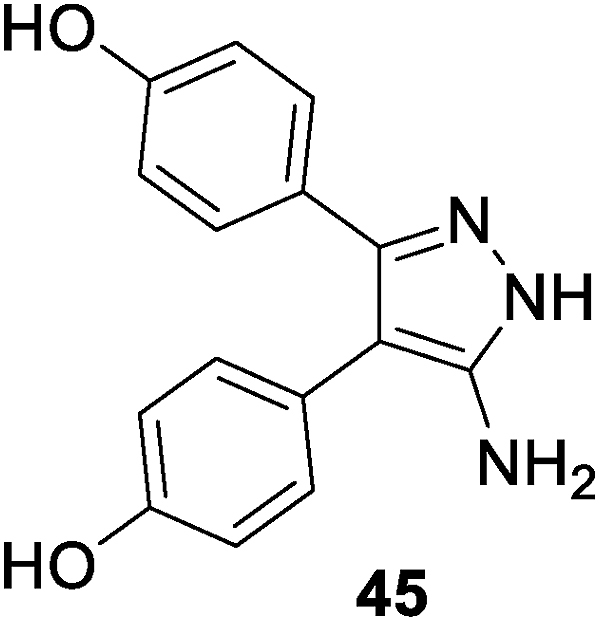
Assay standard used for ITC.

Despite the inability to determine affinity data for **14** against CYP121A1 by UV–Vis, we looked to develop **14** due to the quality of X-ray data and novelty of binding mode coupled with the promising *in cellulo* activity shown by the compound, and so investigated other biophysical methods to determine the *in vitro* potency. Isothermal titration calorimetry (ITC) was attempted with **14**, and the data compared to those obtained in a similar experiment using a known compound **45** (Fig. 6), for which affinity had been measured previously *via* this method (*K*_d_: 40 μM) [9]. In these experiments, **14** showed lower affinity (*K*_d_: ∼100 μM) than standard **45**, which showed a potency (*K*_d_: 20–25 μM) in line with that measured previously (for ITC thermograms, see SI). With **14**, exotherms measured by ITC were relatively small, perhaps as a result of energy being required to disrupt the highly ordered network of waters displaced by the indolyl moiety (greater entropic component to the binding event). Recent studies have highlighted that compound binding affinities measured in bioassays can be hugely influenced by the presence or lack of key water molecules in the active site [20].

Comparisons of affinities generated for compounds by UV–Vis and ITC in our laboratories against CYP121A1 have indicated that invariably those affinities from ITC are a factor of 10–50 lower than those measured by UV–Vis, a prime example being ITC assay standard **45** (as well as several other members of this 3-aminopyrazole based series). Additionally, ITC requires larger quantities of protein than UV–Vis, and as such is not always suitable for the generation of large quantities of affinity data from multiple analogous compounds. Nevertheless, the indicative value for the binding affinity of **14** against CYP121A1 from ITC proved useful, as both the X-ray data and *Mtb* strain H37Rv pan-assay activity for this compound were key to our strategy for compound elaboration.

**Fig. 7:**
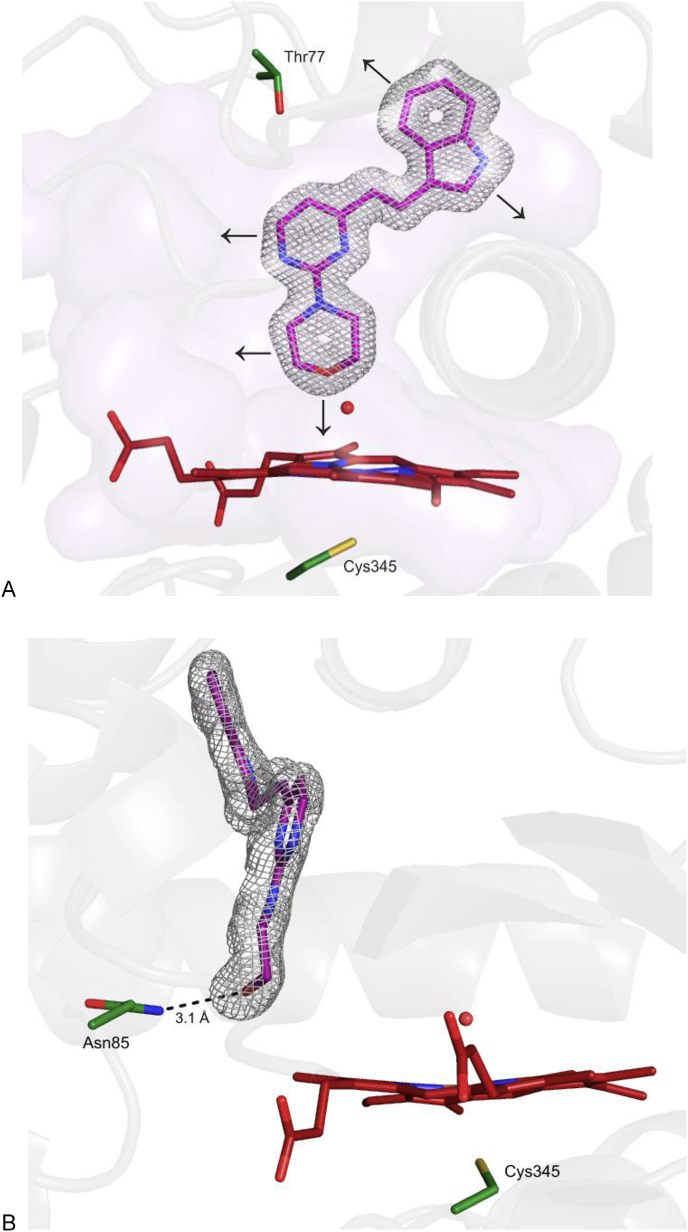
(A) Hit expansion of **14**. Arrows indicate areas targeted for compound elaboration (and specific substitution patterns chosen) in order to conserve binding mode. The sidechain of Thr77 is highlighted. (b) Specific conformation of the morpholine sidechain of **14** highlighting the hydrogen bond between the sidechain NH_2_ group of Asn85. 2F_o_-F_c_ map is shown as a fine mesh contoured to one sigma. A possible hydrogen bond is shown as a black dash. Water molecules are shown as red spheres.

The X-ray structure for **14** was utilized in a structure-based design campaign with a view to generate analogous derivatives with improved affinity against *Mtb*. In this hit expansion phase, a number of novel compounds were synthesized, each of which retained all 23 heavy atoms of the hit **14**. By retaining these key atoms, one could expect the resulting compounds to adopt a similar conformation upon binding to CYP121A1, with each derivative making comparable key contacts to the enzyme. Based upon the X-ray structure (Fig. 7, Panel A), **14** was elaborated in five key areas , by adding a limited number of heavy atoms to the hit structure (≤5). Additionally, the specific conformation of the morpholine ring upon engagement with the sidechain of Asn85 (Fig. 7, Panel B) was factored into the design strategy.

Compounds **51**–**65** were prepared using methods similar to those described above (for **11**–**33**). In view of the uncertainty of exactly how large a substituent at C-5 of the indole ring could be tolerated, a number of derivatives bearing a variety of functionalities of differing sizes were prepared. Reactions between 2-chloro-4-vinylpyrimidine [16] and the appropriately 5-substituted-1*H*-indole gave the fluoro, methoxy, trifluoromethoxy and benzyloxy intermediates (**46**–**49** respectively) which were reacted with morpholine in hot ethanol to afford **51**–**54**. Hydrogenation of **54** over palladium on activated carbon afforded the hydroxy derivative **55**. Reduction of bromide **21** under an atmosphere of deuterium with added base allowed for the preparation of the specifically deuterated derivative **56** (∼95% *d* incorporation). Substitution at N-1 on the indole ring with a methyl group was achieved by similar means affording **57** (*via* **50**). Direct derivatization at the indole nitrogen of **14** required more forceful conditions; acetyl **64** (refluxing acetic anhydride) and methanesulphonyl **65** (MsCl with concentrated base under phase transfer conditions) [21] derivatives were prepared thus. Bridged bicyclic derivatives **58** and **59** (aimed at locking in the morpholine conformation noted above) and the ring expanded homologue **60** were prepared from the appropriate 2-chloropyrimidine **(11** and **46**) and the corresponding secondary amine (or amine hydrochloride salt) in hot ethanol. Benzo[*b*][1,4]oxazines **61**–**63** were prepared from chlorides **11** and **46** by similar means, but required more vigorous conditions (120 °C in a sealed tube or 160 °C with microwave irradiation) due to the much reduced nucleophilicities of the aromatic amines.

Compounds **51**–**65** were screened crystallographically against CYP121A1, but unfortunately in each case no ligand density was observed, although it was seen that the lattice of resting state waters had been partially or completely disrupted. Compounds **51**–**65** were screened for *in vitro* activity against CYP121A1 using UV–Vis spectroscopy. As with the initial X-ray hit **14**, none of these direct analogues showed any discernible effect (no Soret band shift), suggesting that, like **14**, they bind to CYP121A1 without direct contact to the heme. Likewise, a number of the compounds were examined by ITC, but it proved difficult to generate affinity data for these molecules using ITC due to inadequate solubility in aqueous buffers.

Despite this, a number of analogues of **14** were screened against *Mtb* (Table 1). Several derivatives (**31**, **51**, **53**, **57**, **60**, **61** and **63**) were moderate inhibitors, and like **14**, showed activity in all three of the growth media tested. Bridged bicyclic derivative **58** was active, but showed much reduced potency in BSA rich medium. *N*-Acetyl derivative **64** had essentially lost all activity, whereas the corresponding *N*-methyl derivative **57** was one of the compounds that exhibited pan-assay activity. It was noted that the most potent derivative against *Mtb* was **61** (MIC_90_ 6.25 μM), a compound that had been designed based upon the overlap of X-ray crystal structures for **14** and **31**, where the ligand in **31** had been positioned in an alternative orientation, with the ligand rotated 180° in the active site, so suggesting the idea of appending an aromatic ring onto the morpholine moiety.

The difficulty of obtaining X-ray crystal structures for many of these *in cellulo* active compounds is perhaps not too surprising. Despite there being numerous CYP121A1 crystal structures deposited in the PDB, the majority are with ligands that either have low cLogP or make direct contact with the iron atom of the heme group. As the substrate for the enzyme (cYY) has a cLogP of 0.82, the enzyme is clearly designed to accommodate substrate and product that are highly hydrophilic in nature. Recent studies recording imidazolyl- and triazolyl-derived pyrazoles as inhibitors of CYP121A1 have shown that, whilst compounds with higher lipophilicity (cLogP >4) showed better activity *in cellulo*, only some of those with much lower cLogP (1.44 and 2.68) afforded useable crystal structures [22]. These opposing factors clearly constitute a significant challenge in this area, as compounds that deliver the greatest structural knowledge struggle to permeate the highly lipophilic mycolic acid derived cell wall of *Mtb* and so invariably show much lower *in cellulo* potencies.

In order to obtain a greater degree of structural confidence for this *in cellulo* active series of compounds, derivatives akin to **14** were prepared that might still be able to adopt similar binding modes but had significantly lower cLogP values. This was achieved by direct replacement of the pyrimidine core of **14** with either a suitably reduced pyrrolo[3,4-*d*]pyrimidine or pyrido[3,4-*d*]pyrimidine. This scaffold morphing approach entailed the relocation of a pyrimidine nitrogen to the alternative ring position where it was suitably positioned to be able to interact with the sidechain hydroxyl group of Thr77 of the protein *via* an additional hydrogen bond, thereby potentially gaining additional affinity (Fig. 7, Panel A). This nitrogen relocation had the added benefit of allowing the addition of another hydrophilic ring onto the structure, so greatly reducing the cLogP values of the resulting fused bicyclic compounds (to between 1.22 and 2.22).

**Scheme 2:**
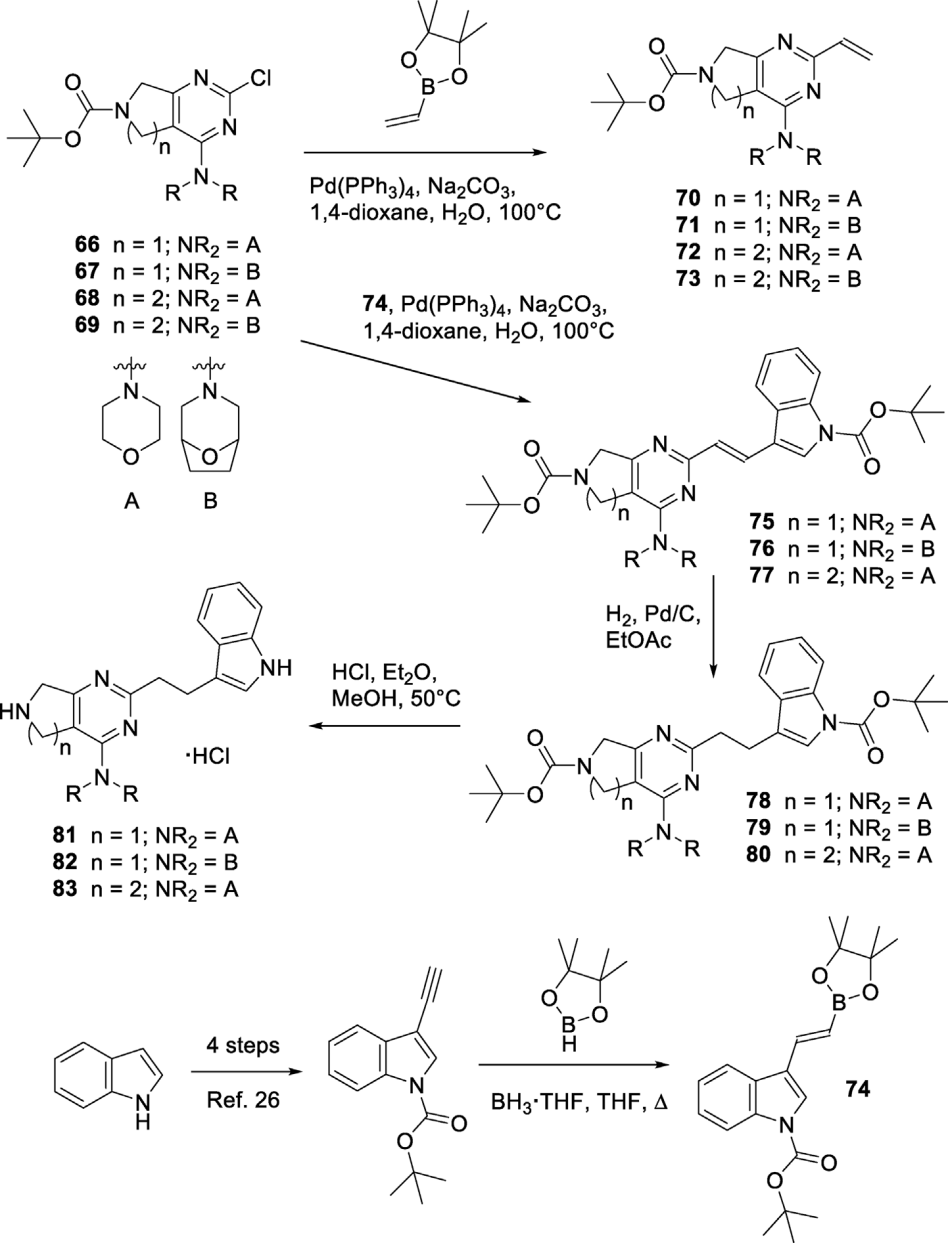
Synthesis of bicyclic analogues **81**–**83**.

Chlorides **66**–**69** were prepared from the corresponding di-chlorides as described previously [23,24]. Palladium(0) catalyzed vinylation was extremely facile, and gave **70**–**73** in high yield under mild conditions. Unfortunately, vinyl derivatives **70**–**73** failed to afford addition products when treated with indoles under conditions identical to those used for the synthesis of **14** and multiple close analogues; instead the alternative vinyl boronate **74** was utilized as a coupling partner. Vinyl boronate **74** is only very briefly described [25] within the patent literature as an (*E*/*Z*)-mixture. When prepared *via* an alternative route over five steps from indole using known chemistry (iodination, *N*-Boc protection, TMS-acetylene addition, fluoride induced TMS removal [26] and borane.THF catalyzed addition of pinacolborane in hot THF [27]), **74** was obtained as the (*E*)-isomer (olefinic coupling constant: *J* = 19 Hz). The added length of synthesis required to generate **74** resulted in only the three derivatives **81**–**83** with lowest cLogP values (1.22–1.77) being targeted. Thus chlorides **66**–**68** were reacted with **74** (under identical conditions to those used to prepare **70**–**73**) to afford **75**–**77** as the (*E*)-isomers (olefinic coupling constants: *J* = 16–17 Hz). Reduction of **75**–**77** with hydrogen over palladium on carbon in ethyl acetate yielded **78**–**80** (after chromatography on silica to remove over-reduced by-products) which, upon deprotection under acidic conditions, gave **81**–**83** (as the hydrochloride salts) (Scheme 2).

Fused bicyclic compounds **81**–**83** behaved, as expected, like all of the pyrimidine derivatives in this series in that we were unable to determine the affinities for these compounds against CYP121A1 by UV–Vis spectroscopy. This was reassuring, as it effectively ruled out the possibility of a change of binding mode in which interaction with the heme iron atom might conceivably occur *via* the cyclic secondary amine functionalities. Likewise, determination of potency by ITC proved difficult for these derivatives (in common with a number of the pyrimidines described above). Despite a significant reduction in cLogP values, it again proved impossible to collect satisfactory X-ray structures for **81**–**83**. Nevertheless, the much improved solubility of **81**–**83** in aqueous media allowed the use of differential scanning fluorimetry to probe the binding affinities of **81**–**83** (for protein melting curves, see SI). Addition of all three bicyclic derivatives to solutions of CYP121A1 in aqueous buffer (1 mM ligand with 5 μM protein) resulted in increases to the melting temperature (Tm) of the protein (Table 3). This suggests that **81**–**83** bound to, and stabilized, the protein. The effect was most pronounced in the two pyrrolo[3,4-*d*]pyrimidine derived compounds **81** (+3.5 °C) and **82** (+2.4 °C), with the ring expanded homologue **83** (+1.0 °C) showing a lesser effect (akin to that of aminopyrimidine **45** used as positive control). The original screening set, together with the set of advanced derivatives (as outlined above), were also subsequently screened by these means. Most compounds gave inconclusive results as a result of aggregation, due to limited ligand solubility in the aqueous buffer. Compounds with lower cLogP proved to be less problematic, with both **10** (an X-ray hit) and **55** (the 5-hydroxy derivative of the key X-ray hit **14**) also showing evidence of activity (protein melting temperature shifts of +3.5 °C and +0.5 °C respectively).

**Table 3:** Differential scanning fluorimetry (DSF) data. Right-hand column shows the increases in protein melting temperatures. Concentrations: CYP121A1 (5 μM), compounds (1 mM).

Compound	Structure	ΔTm (°C)	Compound	Structure	ΔTm (°C)
**10**		+3.5	**81**		+3.5
**45**		+1.0	**82**		+2.4
**55**		+0.5	**83**		+1.0

**Fig. 8:**
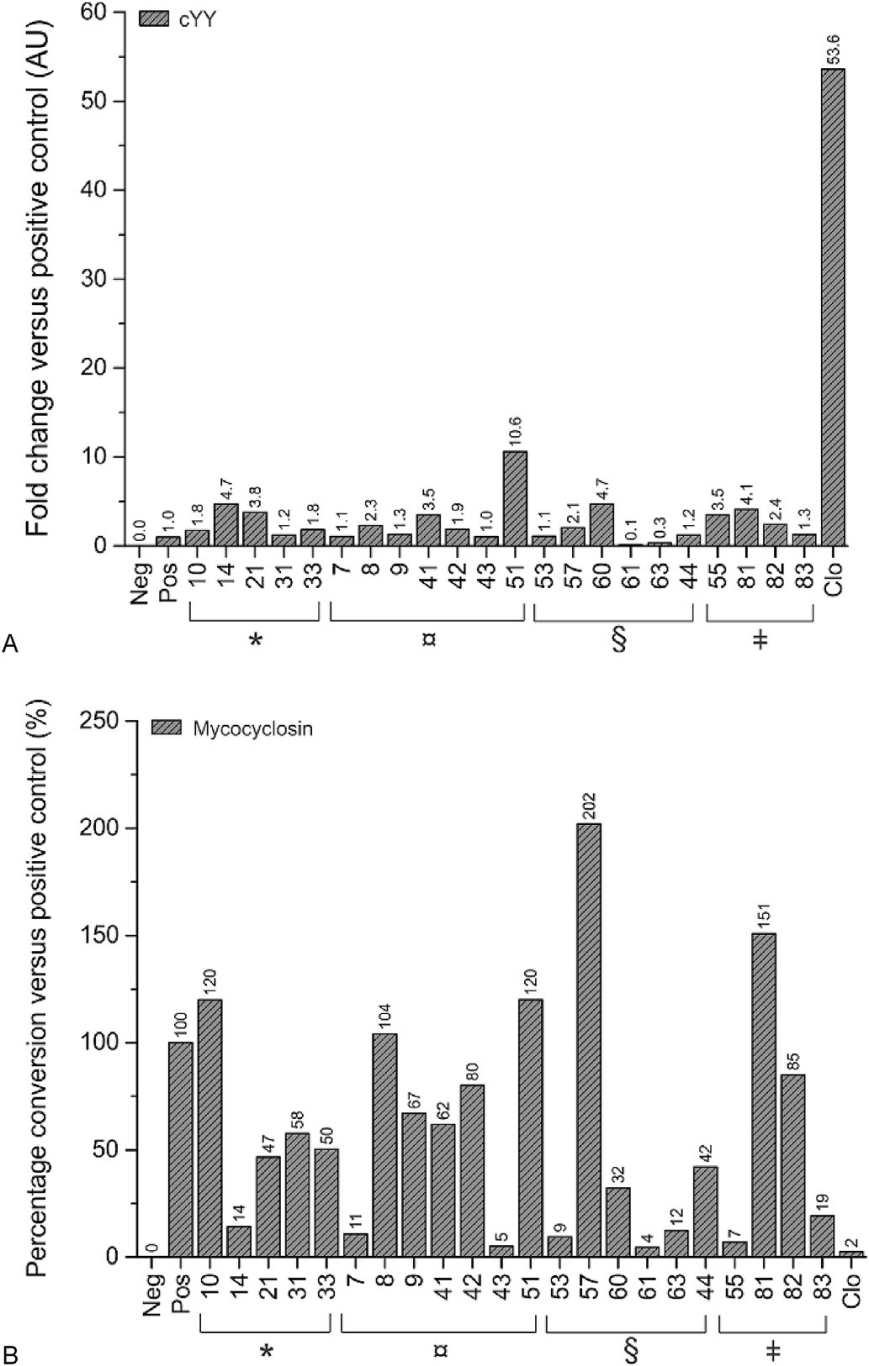
LC-MS activity assay. Columns show the amounts of cYY (a) and mycocyclosin (b) detected (normalized relative to the positive control). Large amounts of cYY and low amounts of mycocyclosin indicate that a compound efficiently inhibits the activity of purified CYP121A1 under the conditions used. Neg: negative control; Pos: positive control; ∗: X-ray crystallographic hits; ¤: UV–Visible spectroscopy hits; §: antimycobacterial activity assay hits; ǂ: differential scanning fluorimetry hits; Clo: clotrimazole. A total of one experiment was performed. The negative control consisted of sample without the introduction of reducing power. The positive control consisted of a sample without the introduction of any inhibitory/experimental compounds.

A panel of key compounds (**7**–**10**, **14**, **21**, **31**, **33**, **41**–**44**, **51**, **53**, **55**, **57**, **60**, **61**, **63**, **81**–**83**) were tested in an LC-MS based activity assay, to determine their ability to inhibit the turnover of cYY into mycocyclosin relative to clotrimazole, a known azole derived inhibitor of CYP121A1. All compounds tested were either X-ray crystallographic hits, or had yielded encouraging data in UV–Visible spectroscopy, differential scanning fluorimetry, isothermal titration calorimetry or antimycobacterial activity assays (as described above). Under the conditions used, no CYP121A1 mediated turnover of the experimental compounds was observed, indicating that none of the compounds were substrates for CYP121A1. As expected, in samples containing both cYY and clotrimazole, a large amount of cYY (>50-fold over the positive control) remained present following incubation (Fig. 8, Panel A), indicating a poor conversion to mycocyclosin (Fig. 8, Panel A). Gratifyingly, in the presence of compounds (**7**–**10**, **14**, **21**, **31**, **33**, **41**–**44**, **51**, **53**, **55**, **57**, **60**, **61**, **63**, **81**–**83**), a general trend towards inhibition of CYP121A1 was noted, thus confirming inhibitory activity of CYP121A1 as a general property of a number of key derivatives within the series of compounds. In particular, **14** (the original hit from the XP screen) and **61** (with pan-assay activity against whole cell *Mtb*) both markedly reduced the production of mycocyclosin (Fig. 8, Panel B), thus reinforcing the suitability of **61** as a starting point for further development.

Given their respective positions within the medicinal chemistry campaign, the propensity of compounds **14** and **61** to occlude the active site, and therefore prevent cYY from binding, was quantified using a UV–Visible spectrophotometric competition assay (Fig. S5). In the competition assays, compounds **14** and **61** were introduced prior to a titration with cYY; in the reverse competition assays, cYY was introduced prior to a titration with either **14** or **61**. A control titration with cYY produced a *K*_d_ of 17.2 ± 0.6 *μ*M, a value akin to that reported previously [4]. An increase in the *K*_d_ value for cYY in the presence of **14** or **61** would indicate that the compounds were occluding the active site of CYP121A1 to a significant enough degree, so as to preclude the simultaneous binding of cYY. Indeed, the presence of **14** appeared to perturb the binding of cYY, resulting in a *K*_d_ value of 25.2 ± 1.0 *μ*M, a 1.5-fold increase over the cYY control (Table 4). The presence of **61**, however, did not appear to greatly perturb the binding of cYY, an observation that is seemingly inconsistent with the LC-MS activity and antimycobacterial assays, but might be indicative of a more limited solubility of **61** (relative to **14**) under the assay conditions.

**Table 4:** UV–Visible spectrophotometric competition assay. Dissociation constants (*K*_d_) for cYY (screened at 15 mM) and competition assays with cYY (30 mM) in the presence of **14** (100 mM) or **61** (100 mM). Data were fitted using the Hill equation.

	Structure	*K*_d_ for cYY(μM)	Fold increase
**cYY**		17.2 ± 0.6	-
**14**		25.2 ± 1.0	1.5
**61**		17.5 ± 0.5	-

## 3 Conclusions

X-ray crystallographic screening is a frequently used technique for hit generation in the field of fragment-based drug discovery [11,28] with a noticeable trend towards the use of the technique to screen increasingly smaller compounds [29]. Recently, X-ray crystallography has been successfully explored in the absence of crystal cryo-protection [30] in order to further aid screening throughput. Such techniques, however, are unlikely to yield hit compounds showing appreciable levels of cellular activity, due to their small size. As such, a purely target-based screening approach suffers in that it is possible to undertake a substantial amount of discovery research with only a relatively limited knowledge of *in cellulo* or *in vivo* activity, with compounds being progressed that lack acceptable cell permeability, or poor ADMET properties.

Recent examples of a more typical phenotypic based screening approach against *Mtb* have involved open-source initiatives utilizing the diverse array of compounds residing within corporate collections to provide lead compounds. These efforts have identified a series of 2-thiophenyl morpholines [31] (from the Eli Lilly collection), novel spirocyclic derivatives [32] and pyrrolothiadiazoles [33] (both from the GSK collection). In addition, spirocycles containing 3-substituted indoles [34] (that possess high membrane permeability) have also recently been described as having excellent activity against *Mtb*. These approaches invariably require a more traditional medicinal chemistry approach to compound progression, from larger and more chemically advanced compounds, *via* the generation of multiple rounds of structure-activity relationship (SAR) data, often with very limited knowledge of precise target engagement.

Novel approaches towards new anti-TB agents in our laboratories have focused heavily on structure-based design against new targets, a recent case in point [35,36] being *Mtb* fumarate hydratase (fumarase) in which inhibitors with a dimeric binding mode at an allosteric site were utilized to affect *Mtb* viability. In the current paper we describe a novel screening approach (XP screen), that utilizes a combination of X-ray crystallographic screening of a focused compound set against a chosen target (illustrated here with CYP121A1) and phenotypic screening to ascertain *in cellulo* compound activity. This bidirectional approach, which addresses the problem of finding novel chemical hits from both ends of the traditional screening cascade at a very early stage, is an effective method by which to rapidly triage those compounds that are not observed crystallographically (those for which target engagement in unassured) together with those that possess only very limited *in cellulo* activity.

In summary, we have used an XP screen against CYP121A1, in combination with a variety of biophysical techniques, to identify novel *in cellulo* active inhibitors of this key target [37] in anti-TB research. Structure-based design around a key X-ray crystallographic hit **14** led to a number of close structural analogues that showed pan-assay activity against *Mtb*. One of these, the benzo[*b*][1,4]oxazine derivative **61**, is a novel lead compound of moderate molecular mass (*M*_r_ = 356) suitable as a starting point for a more thorough lead-to-clinical candidate phase, that inhibits the CYP121A1 mediated turnover of cYY to mycocyclosin, and shows favorable activity against *Mtb* strain H37Rv (MIC_90_ ∼6.25 μM, ∼2.2 μg/mL) in comparison with a number of azole anti-fungal drugs (clotrimazole, econazole, miconazole: MIC_90_ 11, 8, and 8 μg/mL respectively) [7,38] that also act *via* inhibition of CYP121A1 [7], but that suffer from poor oral bioavailability or show unacceptable toxicity profiles.

## 4 Experimental

### 4.1 Chemistry

Petroleum ether (b.p. 40–60 °C), ethyl acetate, *n*-hexane, dichloromethane and toluene were distilled prior to use. *N*-Bromosuccinimide was recrystallized from water and dried over potassium hydroxide pellets under reduced pressure in a vacuum desiccator prior to use. Column chromatography was performed on a Biotage Isolera™ Spektra One automated flash purification system using appropriately sized pre-packed silica cartridges. Nuclear magnetic resonance (NMR) spectra were recorded in DMSO‑*d*_6_ on a Bruker AVANCE III HD 400 spectrometer with a BBO SmartProbe™ operating at 399.6 MHz or 400.1 MHz (for ^1^H), at 100.5 or 100.6 MHz (for ^1^H decoupled ^13^C) and at 376.0 MHz (for ^1^H decoupled ^19^F) or on a Bruker AVANCE NEO 400 spectrometer with a Prodigy BBO CryoProbe™ operating at 128.4 MHz (for ^11^B) and at 100.6 MHz (for ^1^H decoupled ^13^C). Liquid chromatography-mass spectrometry (LC-MS) data were recorded on a Waters Acquity™ H-Class Ultra Performance Liquid Chromatography system with a tunable, dual wavelength UV–Visible detector coupled to a single quadrupole mass detector (analytical column: Waters Cortecs® UPLC® C18 + 90 Å, 1.6 μm, 2.1 mm × 50 mm; solvent gradient: 5–95% acetonitrile in 0.1% v/v aqueous formic acid; flow rate: 0.8 ml/min; elution run time: 4 min) and are reported as ions with mass(es) (*R*_t_, area %). All compounds (with the exception of compound **27**) are ≥95% pure by HPLC analysis. High resolution mass spectra (HRMS) were recorded on a Waters Vion™ Ion Mobility Q-TOF mass spectrometer using electrospray ionization or on a Waters Xevo® G2-S bench top Q-TOF mass spectrometer using electrospray ionization and are reported as ions with mass, calculated mass and relative error (in parts per million).

Compounds **4**, [12], **6**, [14] **7**, [12] **8**, [13] **9**, [15] **10**, [13] **11**, [13] **41**, [19] **43**, [15] **45**, [9] **66**, [23] **67**, [24] **68** [24] and **69** [24] were prepared as described previously.

*5-Chloro-3-(2-(pyridin-*2-yl*)ethyl)-1H-indole (****5****).* Prepared as per the method for **8** [13] from 2-vinylpyridine and 5-chloro-1*H*-indole. Yield 65%. Off-white solid. ^1^H NMR: δ 10.96 (br s, 1H), 8.51 (ddd, *J* = 5, 2, 1 Hz, 1H), 7.67 (td, *J* = 7.5, 2 Hz, 1H), 7.50 (d, *J* = 2 Hz, 1H), 7.33 (d, *J* = 9 Hz, 1H), 7.28 (dt, *J* = 7.5, 1 Hz, 1H), 7.20 (ddd, *J* = 7.5, 5, 1 Hz, 1H), 7.17 (d, *J* = 2 Hz, 1H), 7.05 (dd, *J* = 9, 2 Hz, 1H), 3.07 (s, 4H). ^13^C NMR: δ 161.7 (C), 149.4 (CH), 136.8 (CH), 135.1 (C), 128.8 (C), 124.7 (CH), 123.4 (C), 123.3 (CH), 121.7 (CH), 121.2 (CH), 118.1 (CH), 114.5 (C), 113.3 (CH), 38.7 (CH_2_), 25.1 (CH_2_). LC-MS: [M + H]^+^ 257, 259 (1.75, 100). HRMS: [M + H]^+^ 257.0837. Calcd for C_15_H_14_^35^ClN_2_^+^: 257.0840. Δ = −1.2.

*5-Chloro-3-(2-(2-chloropyrimidin-*4-yl*)ethyl)-1H-indole (****12****)*. Prepared as per the method for **11** [13] from 2-chloro-4-vinylpyrimidine [16] and 5-chloro-1*H*-indole. Yield 66%. Yellow solid. ^1^H NMR: δ 11.00 (br s, 1H), 8.62 (d, *J* = 5 Hz, 1H), 7.58 (d, *J* = 2 Hz, 1H), 7.48 (d, *J* = 5 Hz, 1H), 7.34 (d, *J* = 8.5 Hz, 1H), 7.21 (d, *J* = 2 Hz, 1H), 7.06 (dd, *J* = 8.5, 2 Hz, 1H), 3.10 (s, 4H). ^13^C NMR: δ 174.6 (C), 160.4 (CH), 160.4 (C), 135.1 (C), 128.6 (C), 125.0 (CH), 123.5 (C), 121.4 (CH), 120.4 (CH), 118.1 (CH), 113.5 (C), 113.4 (CH), 37.8 (CH_2_), 23.7 (CH_2_). LC-MS: [M + H]^+^ 292, 294, 296 (2.07, 100). HRMS: [M + H]^+^ 292.0403. Calcd for C_14_H_12_^35^Cl_2_N_3_^+^: 292.0403. Δ = 0.

*5-Bromo-3-(2-(2-chloropyrimidin-*4-yl*)ethyl)-1H-indole (****13****)*. Prepared as per the method for **11** [13] from 2-chloro-4-vinylpyrimidine [16] and 5-bromo-1*H*-indole. Yield 42%. Yellow solid. ^1^H NMR: δ 11.02 (br s, 1H), 8.62 (d, *J* = 5 Hz, 1H), 7.71 (d, *J* = 2 Hz, 1H), 7.48 (d, *J* = 5 Hz, 1H), 7.30 (d, *J* = 8.5 Hz, 1H), 7.19 (d, *J* = 2 Hz, 1H), 7.17 (dd, *J* = 8.5, 2 Hz, 1H), 3.10 (s, 4H). ^13^C NMR: δ 174.6 (C), 160.4 (CH), 160.4 (C), 135.3 (C), 129.3 (C), 124.9 (CH), 123.9 (CH), 121.1 (CH), 120.4 (CH), 113.9 (CH), 113.5 (C), 111.5 (C), 37.8 (CH_2_), 23.6 (CH_2_). LC-MS: [M + H]^+^ 336, 338, 340 (2.11, 100). HRMS: [M + Na]^+^ 357.9716. Calcd for C_14_H_11_^79^Br^35^ClN_3_Na^+^: 357.9717. Δ = −0.3.

*4-(4-(2-(1H-Indol-*3-yl*)ethyl)pyrimidin-*2-yl*)morpholine (****14****)*. A mixture of 3-(2-(2-chloropyrimidin-4-yl)ethyl)-1*H*-indole **11** (103 mg, 0.4 mmol) and morpholine (1 mL) in ethanol (6 mL) was stirred and held at reflux for 4 h and allowed to cool to room temperature. The solvent was removed *in vacuo* and the residues partitioned between dichloromethane and water. The organic layer was separated, the solvent removed *in vacuo* and the residues subjected to column chromatography on silica. Elution with 0–100% ethyl acetate in petroleum ether (b.p. 40–60 °C) afforded 4-(4-(2-(1*H*-indol-3-yl)ethyl)pyrimidin-2-yl)morpholine **14** (90 mg, 73%) as a pale yellow solid. ^1^H NMR: δ 10.75 (br s, 1H), 8.24 (d, *J* = 5 Hz, 1H), 7.53 (d, *J* = 8 Hz, 1H), 7.33 (d, *J* = 8 Hz, 1H), 7.11 (d, *J* = 2 Hz, 1H), 7.06 (t, *J* = 8 Hz, 1H), 6.97 (t, *J* = 8 Hz, 1H), 6.61 (d, *J* = 5 Hz, 1H), 3.70 (m, 4H), 3.66 (m, 4H), 3.08 (m, 2H), 2.93 (m, 2H). ^13^C NMR: δ 170.9 (C), 161.8 (C), 158.0 (CH), 136.7 (C), 127.5 (C), 122.8 (CH), 121.3 (CH), 118.7 (CH), 118.6 (CH), 114.2 (C), 111.8 (CH), 110.0 (CH), 66.5 (CH_2_), 44.3 (CH_2_), 38.2 (CH_2_), 23.9 (CH_2_). LC-MS: [M + H]^+^ 309 (1.96, 96). HRMS: [M + H]^+^ 309.1714. Calcd for C_18_H_21_N_4_O^+^: 309.1710. Δ = +1.2.

*4-(4-(2-(1H-Indol-*3-yl*)ethyl)pyrimidin-*2-yl*)thiomorpholine (****15****)*. Prepared as per the method for **14** from 3-(2-(2-chloropyrimidin-4-yl)ethyl)-1*H*-indole **11** and thiomorpholine. Yield 74%. Colorless solid. ^1^H NMR: δ 10.77 (br s, 1H), 8.23 (d, *J* = 5 Hz, 1H), 7.51 (d, *J* = 8 Hz, 1H), 7.32 (d, *J* = 8 Hz, 1H), 7.11 (d, *J* = 2 Hz, 1H), 7.06 (t, *J* = 8 Hz, 1H), 6.97 (t, *J* = 8 Hz, 1H), 6.58 (d, *J* = 5 Hz, 1H), 4.07 (m, 4H), 3.07 (t, *J* = 6.5 Hz, 2H), 2.94 (t, *J* = 6.5 Hz, 2H), 2.59 (m, 4H). ^13^C NMR: δ 171.0 (C), 161.2 (C), 158.1 (CH), 136.7 (C), 127.5 (C), 122.8 (CH), 121.3 (CH), 118.7 (CH), 118.6 (CH), 114.1 (C), 111.8 (CH), 109.6 (CH), 46.3 (CH_2_), 38.1 (CH_2_), 26.4 (CH_2_), 24.0 (CH_2_). LC-MS: [M + H]^+^ 325 (2.20, 100). HRMS: [M + H]^+^ 325.1478. Calcd for C_18_H_21_N_4_^32^S^+^: 325.1481. Δ = −1.0.

*3-(2-(2-(4-Methylpiperazin-*1-yl*)pyrimidin-*4-yl*)ethyl)-1H-indole (****16****)*. A mixture of 3-(2-(2-chloropyrimidin-4-yl)ethyl)-1*H*-indole **11** (129 mg, 0.5 mmol) and *N*-methylpiperazine (1 mL) in ethanol (6 mL) was stirred and held at reflux for 4 h and allowed to cool to room temperature. The solvent was removed *in vacuo* and the residues partitioned between dichloromethane and water. The organic layer was separated, the solvent removed *in vacuo* and the residues triturated with a mixture of *n*-hexane and dichloromethane. The solids were collected by suction filtration to afford 3-(2-(2-(4-methylpiperazin-1-yl)pyrimidin-4-yl)ethyl)-1*H*-indole **16** (150 mg, 93%) as a pale yellow solid. ^1^H NMR: δ 10.75 (br s, 1H), 8.20 (d, *J* = 5 Hz, 1H), 7.52 (d, *J* = 8 Hz, 1H), 7.32 (d, *J* = 8 Hz, 1H), 7.11 (d, *J* = 2 Hz, 1H), 7.06 (t, *J* = 8 Hz, 1H), 6.97 (t, *J* = 8 Hz, 1H), 6.56 (d, *J* = 5 Hz, 1H), 3.74 (m, 4H), 3.07 (m, 2H), 2.92 (m, 2H), 2.35 (m, 4H), 2.21 (s, 3H). ^13^C NMR: δ 170.8 (C), 161.7 (C), 158.0 (CH), 136.7 (C), 127.5 (C), 122.8 (CH), 121.3 (CH), 118.7 (CH), 118.6 (CH), 114.2 (C), 111.8 (CH), 109.6 (CH), 54.9 (CH_2_), 46.4 (CH_3_), 43.7 (CH_2_), 38.2 (CH_2_), 23.9 (CH_2_). LC-MS: [M + H]^+^ 322 (1.40, 100). HRMS: [M + H]^+^ 322.2023. Calcd for C_19_H_24_N_5_^+^: 322.2026. Δ = −1.0.

*3-(2-(2-(4-(2,2,2-Trifluoroethyl)piperazin-*1-yl*)pyrimidin-*4-yl*)ethyl)-1H-indole (****17****)*. Prepared as per the method for **31** from 3-(2-(2-chloropyrimidin-4-yl)ethyl)-1*H*-indole **11** and 1-(2,2,2-trifluoroethyl)piperazine dihydrochloride. Yield 67%. Colorless solid. ^1^H NMR: δ 10.75 (br s, 1H), 8.22 (d, *J* = 5 Hz, 1H), 7.53 (d, *J* = 8 Hz, 1H), 7.33 (d, *J* = 8 Hz, 1H), 7.12 (d, *J* = 2 Hz, 1H), 7.06 (t, *J* = 8 Hz, 1H), 6.97 (t, *J* = 8 Hz, 1H), 6.58 (d, *J* = 5 Hz, 1H), 3.75 (m, 4H), 3.22 (q, *J* = 10 Hz, 2H), 3.07 (t, *J* = 7.5 Hz, 2H), 2.92 (t, *J* = 7.5 Hz, 2H), 2.67 (m, 4H). ^13^C NMR: δ 170.9 (C), 161.6 (C), 158.0 (CH), 136.7 (C), 127.5 (C), 126.5 (q, *J* = 280 Hz, CF_3_), 122.8 (CH), 121.3 (CH), 118.7 (CH), 118.6 (CH), 114.2 (C), 111.8 (CH), 109.7 (CH), 57.4 (q, *J* = 29 Hz, CH_2_CF_3_), 53.4 (CH_2_), 43.8 (CH_2_), 38.2 (CH_2_), 24.0 (CH_2_). ^19^F NMR: δ −67.8. LC-MS: [M + H]^+^ 390 (2.29, 100). HRMS: [M + H]^+^ 390.1902. Calcd for C_20_H_23_F_3_N_5_^+^: 390.1900. Δ = +0.5.

*4-(4-(2-(5-Chloro-1H-indol-*3-yl*)ethyl)pyrimidin-*2-yl*)morpholine (****18****)*. Prepared as per the method for **14** from 5-chloro-3-(2-(2-chloropyrimidin-4-yl)ethyl)-1*H*-indole **12** and morpholine. Yield 44%. Pale yellow solid. ^1^H NMR: δ 10.97 (br s, 1H), 8.22 (d, *J* = 5 Hz, 1H), 7.52 (d, *J* = 2 Hz, 1H), 7.33 (d, *J* = 8.5 Hz, 1H), 7.20 (d, *J* = 2.5 Hz, 1H), 7.04 (dd, *J* = 8.5, 2.5 Hz, 1H), 6.59 (d, *J* = 5 Hz, 1H), 3.70 (m, 4H), 3.65 (m, 4H), 3.06 (t, *J* = 8 Hz, 2H), 2.91 (t, *J* = 8 Hz, 2H). ^13^C NMR: δ 170.7 (C), 161.8 (C), 158.0 (CH), 135.1 (C), 128.7 (C), 124.8 (CH), 123.4 (C), 121.2 (CH), 118.1 (CH), 114.2 (C), 113.3 (CH), 110.1 (CH), 66.5 (CH_2_), 44.3 (CH_2_), 38.2 (CH_2_), 23.6 (CH_2_). LC-MS: [M + H]^+^ 343, 345 (2.11, 100). HRMS: [M + H]^+^ 343.1318. Calcd for C_18_H_20_^35^ClN_4_O^+^: 343.1320. Δ = −0.2.

*4-(4-(2-(5-Chloro-1H-indol-*3-yl*)ethyl)pyrimidin-*2-yl*)thiomorpholine (****19****)*. Prepared as per the method for **14** from 5-chloro-3-(2-(2-chloropyrimidin-4-yl)ethyl)-1*H*-indole **12** and thiomorpholine. Yield 66%. Pale orange solid. ^1^H NMR: δ 10.97 (br s, 1H), 8.21 (d, *J* = 5 Hz, 1H), 7.50 (d, *J* = 2 Hz, 1H), 7.33 (d, *J* = 8.5 Hz, 1H), 7.20 (d, *J* = 2.5 Hz, 1H), 7.04 (dd, *J* = 8.5, 5 Hz, 1H), 6.55 (d, *J* = 5 Hz, 1H), 4.07 (m, 4H), 3.06 (m, 2H), 2.90 (m, 2H), 2.58 (m, 4H). ^13^C NMR: δ 170.9 (C), 161.2 (C), 158.1 (CH), 135.1 (C), 128.7 (C), 124.8 (CH), 123.4 (C), 121.2 (CH), 118.0 (CH), 114.2 (C), 113.3 (CH), 109.7 (CH), 46.3 (CH_2_), 38.2 (CH_2_), 26.4 (CH_2_), 23.6 (CH_2_). LC-MS: [M + H]^+^ 359, 361 (2.33, 100). HRMS: [M + H]^+^ 359.1088. Calcd for C_18_H_20_^35^ClN_4_^32^S^+^: 359.1092. Δ = −1.2.

*5-Chloro-3-(2-(2-(4-methylpiperazin-*1-yl*)pyrimidin-*4-yl*)ethyl)-1H-indole (****20****)*. Prepared as per the method for **16** from 5-chloro-3-(2-(2-chloropyrimidin-4-yl)ethyl)-1*H*-indole **12** and *N*-methylpiperazine. Yield 70%. Pale yellow solid. ^1^H NMR: δ 10.97 (br s, 1H), 8.19 (d, *J* = 5 Hz, 1H), 7.52 (d, *J* = 2 Hz, 1H), 7.33 (d, *J* = 8.5 Hz, 1H), 7.20 (d, *J* = 2.5 Hz, 1H), 7.04 (dd, *J* = 8.5, 2.5 Hz, 1H), 6.54 (d, *J* = 5 Hz, 1H), 3.73 (m, 4H), 3.03 (t, *J* = 7 Hz, 2H), 2.89 (t, *J* = 7 Hz, 2H), 2.34 (m, 4H), 2.21 (s, 3H). ^13^C NMR: δ 170.6 (C), 161.7 (C), 158.0 (CH), 135.1 (C), 128.7 (C), 124.8 (CH), 123.4 (C), 121.2 (CH), 118.1 (CH), 114.3 (C), 113.3 (CH), 109.7 (CH), 54.9 (CH_2_), 46.3 (CH_3_), 43.7 (CH_2_), 38.2 (CH_2_), 23.6 (CH_2_). LC-MS: [M + H]^+^ 356, 358 (1.50, 98). HRMS: [M + H]^+^ 356.1631. Calcd for C_19_H_23_^35^ClN_5_^+^: 356.1636. Δ = −1.5.

*4-(4-(2-(5-Bromo-1H-indol-*3-yl*)ethyl)pyrimidin-*2-yl*)morpholine (****21****)*. Prepared as per the method for **14** from 5-bromo-3-(2-(2-chloropyrimidin-4-yl)ethyl)-1*H*-indole **13** and morpholine. Yield 65%. Colorless solid. ^1^H NMR: δ 10.98 (br s, 1H), 8.22 (d, *J* = 5 Hz, 1H), 7.66 (d, *J* = 2 Hz, 1H), 7.29 (d, *J* = 8.5 Hz, 1H), 7.19 (d, *J* = 2.5 Hz, 1H), 7.15 (dd, *J* = 8.5, 2.5 Hz, 1H), 6.59 (d, *J* = 5 Hz, 1H), 3.71 (m, 4H), 3.65 (m, 4H), 3.06 (t, *J* = 7 Hz, 2H), 2.90 (t, *J* = 7 Hz, 2H). ^13^C NMR: δ 170.7 (C), 161.8 (C), 158.0 (CH), 135.3 (C), 129.4 (C), 124.6 (CH),. 123.8 (CH), 121.1 (CH), 114.2 (C), 113.8 (CH), 111.3 (C), 110.1 (CH), 66.5 (CH_2_), 44.3 (CH_2_), 38.2 (CH_2_), 23.5 (CH_2_). LC-MS: [M + H]^+^ 387, 389 (2.14, 100). HRMS: [M + H]^+^ 387.0818. Calcd for C_18_H_20_^79^BrN_4_O^+^: 387.0815. Δ = +0.7.

*4-(4-(2-(5-Bromo-1H-indol-*3-yl*)ethyl)pyrimidin-*2-yl*)thiomorpholine (****22****)*. Prepared as per the method for **14** from 5-bromo-3-(2-(2-chloropyrimidin-4-yl)ethyl)-1*H*-indole **13** and thiomorpholine. Yield 47%. Off-white solid. ^1^H NMR: δ 10.98 (br s, 1H), 8.21 (d, *J* = 5 Hz, 1H), 7.63 (d, *J* = 2 Hz, 1H), 7.29 (d, *J* = 8.5 Hz, 1H), 7.18 (d, *J* = 2.5 Hz, 1H), 7.15 (dd, *J* = 8.5, 2.5 Hz, 1H), 6.55 (d, *J* = 5 Hz, 1H), 4.07 (m, 4H), 2.58 (m, 4H), 3.05 (t, *J* = 7 Hz, 2H), 2.90 (t, *J* = 7 Hz, 2H). ^13^C NMR: δ 170.8 (C), 161.2 (C), 158.1 (CH), 135.3 (C), 129.5 (C), 124.6 (CH), 123.8 (CH), 121.1 (CH), 114.2 (C), 113.8 (CH), 111.3 (C), 109.7 (CH), 46.2 (CH_2_), 38.2 (CH_2_), 26.4 (CH_2_), 23.6 (CH_2_). LC-MS: [M + H]^+^ 403, 405 (2.36, 100). HRMS: [M + H]^+^ 403.0581. Calcd for C_18_H_20_^79^BrN_4_^32^S^+^: 403.0586. Δ = −1.3.

*5-Bromo-3-(2-(2-(4-methylpiperazin-*1-yl*)pyrimidin-*4-yl*)ethyl)-1H-indole (****23****)*. Prepared as per the method for **16** from 5-bromo-3-(2-(2-chloropyrimidin-4-yl)ethyl)-1*H*-indole **13** and *N*-methylpiperazine. Yield 63%. Orange oil. ^1^H NMR: δ 10.98 (br s, 1H), 8.19 (d, *J* = 5 Hz, 1H), 7.65 (d, *J* = 2 Hz, 1H), 7.29 (d, *J* = 8.5 Hz, 1H), 7.18 (d, *J* = 2.5 Hz, 1H), 7.15 (dd, *J* = 8.5, 2.5 Hz, 1H), 6.54 (d, *J* = 5 Hz, 1H), 3.73 (m, 4H), 3.05 (t, *J* = 7 Hz, 2H), 2.89 (t, *J* = 7 Hz, 2H), 2.34 (m, 4H), 2.21 (s, 3H). ^13^C NMR: δ 170.6 (C), 161.7 (C), 158.0 (CH), 135.3 (C), 129.5 (C), 124.6 (CH), 123.8 (CH), 121.1 (CH), 114.2 (C), 113.8 (CH), 111.3 (C), 109.7 (CH), 54.9 (CH_2_), 46.4 (CH_3_), 43.7 (CH_2_), 38.2 (CH_2_), 23.6 (CH_2_). LC-MS: [M + H]^+^ 400, 402 (1.50, 100). HRMS: [M + H]^+^ 400.1123. Calcd for C_19_H_23_^79^BrN_5_^+^: 400.1132. Δ = +2.2.

*5-Bromo-3-(2-(2-(4-(2,2,2-trifluoroethyl)piperazin-*1-yl*)pyrimidin-*4-yl*)ethyl)-1H-indole (****24****)*. Prepared as per the method for **31** from 5-bromo-3-(2-(2-chloropyrimidin-4-yl)ethyl)-1*H*-indole **13** and 1-(2,2,2-trifluoroethyl)piperazine dihydrochloride. Yield 75%. Pale yellow solid. ^1^H NMR: δ 10.98 (br s, 1H), 8.20 (d, *J* = 5 Hz, 1H), 7.66 (d, *J* = 2 Hz, 1H), 7.29 (d, *J* = 8.5 Hz, 1H), 7.19 (d, *J* = 2 Hz, 1H), 7.16 (dd, *J* = 8.5, 2 Hz, 1H), 6.56 (d, *J* = 5 Hz, 1H), 3.75 (m, 4H), 3.25 (q, *J* = 10 Hz, 2H), 3.06 (t, *J* = 7.5 Hz, 2H), 2.90 (t, *J* = 7.5 Hz, 2H), 2.67 (m, 4H). ^13^C NMR: δ 170.8 (C), 161.6 (C), 158.0 (CH), 135.3 (C), 129.5 (C), 126.5 (q, *J* = 280 Hz, CF_3_), 124.6 (CH), 123.8 (CH), 121.1 (CH), 114.2 (C), 113.8 (CH), 111.3 (C), 109.8 (CH), 57.4 (q, *J* = 29.5 Hz, CH_2_CF_3_), 53.4 (CH_2_), 43.8 (CH_2_), 38.2 (CH_2_), 23.6 (CH_2_). ^19^F NMR: δ −67.8. LC-MS: [M + H]^+^ 468, 470 (2.44, 100). HRMS: [M + H]^+^ 468.0996. Calcd for C_20_H_22_^79^BrF_3_N_5_^+^: 468.1005. Δ = −2.0.

*tert-Butyl 4-(4-(2-(5-bromo-1H-indol-*3-yl*)ethyl)pyrimidin-*2-yl*)piperazine-1-carboxylate (****25****)*. Prepared as per the method for **16** from 5-bromo-3-(2-(2-chloropyrimidin-4-yl)ethyl)-1*H*-indole **13** and *tert*-butyl piperazine-1-carboxylate. Yield 77%. Pale yellow solid. ^1^H NMR: δ 10.98 (br s, 1H), 8.22 (d, *J* = 5 Hz, 1H), 7.66 (d, *J* = 2 Hz, 1H), 7.29 (d, *J* = 8.5 Hz, 1H), 7.19 (d, *J* = 2 Hz, 1H), 7.15 (dd, *J* = 8.5, 2 Hz, 1H), 6.58 (d, *J* = 5 Hz, 1H), 3.72 (m, 4H), 3.39 (m, 4H), 3.06 (t, *J* = 7.5 Hz, 2H), 2.91 (t, *J* = 7.5 Hz, 2H), 1.43 (s, 9H). ^13^C NMR: δ 170.8 (C), 161.6 (C), 158.0 (CH), 154.4 (C=O), 135.3 (C), 129.5 (C), 124.7 (CH), 123.8 (CH), 121.1 (CH), 114.2 (C), 113.8 (CH), 111.3 (C), 110.1 (CH), 79.5 (C), 43.6 (CH_2_), 40.7 (CH_2_), 38.2 (CH_2_), 28.6 (CH_3_), 23.6 (CH_2_). LC-MS: [M + H]^+^ 486, 488 (2.47, 100). HRMS: [M + Na]^+^ 508.1318. Calcd for C_23_H_28_^79^BrN_5_O_2_Na^+^: 508.1310. Δ = 0.

*5-Bromo-3-(2-(2-(piperazin-*1-yl*)pyrimidin-*4-yl*)ethyl)-1H-indole hydrochloride (****26****)*. Prepared as per the method for **37** from *tert*-butyl 4-(4-(2-(5-bromo-1*H*-indol-3-yl)ethyl)pyrimidin-2-yl)piperazine-1-carboxylate **25**. Yield 70%. Pale orange solid. ^1^H NMR: δ 11.07 (br s, 1H), 9.39 (br s, 2H), 8.28 (d, *J* = 5 Hz, 1H), 7.66 (d, *J* = 2 Hz, 1H), 7.30 (d, *J* = 8.5 Hz, 1H), 7.19 (d, *J* = 2 Hz, 1H), 7.16 (dd, *J* = 8.5, 2 Hz, 1H), 6.70 (d, *J* = 5 Hz, 1H), 3.99 (m, 4H), 3.15 (br m, 4H), 3.07 (t, *J* = 8 Hz, 2H), 2.95 (t, *J* = 8 Hz, 2H). ^13^C NMR: δ 171.6 (C), 160.7 (C), 157.5 (CH), 135.4 (C), 129.5 (C), 124.7 (CH), 123.8 (CH), 121.1 (CH), 114.0 (C), 113.8 (CH), 111.3 (C), 110.8 (CH), 42.8 (CH_2_), 40.8 (CH_2_), 38.2 (CH_2_), 23.5 (CH_2_). LC-MS: [M + H]^+^ 386, 388 (1.51, 100). HRMS: [M + H]^+^ 386.0979. Calcd for C_18_H_21_^79^BrN_5_^+^: 386.0975. Δ = +1.0.

*4-(4-(2-(5-Bromo-1H-indol-*3-yl*)ethyl)pyrimidin-*2-yl*)piperazin-2-one (****27****)*. Prepared as per the method for **16** from 5-bromo-3-(2-(2-chloropyrimidin-4-yl)ethyl)-1*H*-indole **13** and piperazin-2-one. Yield 50%. Colorless solid. ^1^H NMR: δ 10.99 (br s, 1H), 8.25 (d, *J* = 5 Hz, 1H), 8.08 (br s, 1H), 7.66 (d, *J* = 2 Hz, 1H), 7.29 (d, *J* = 8.5 Hz, 1H), 7.19 (d, *J* = 2 Hz, 1H), 7.16 (dd, *J* = 8.5, 2 Hz, 1H), 6.62 (d, *J* = 5 Hz, 1H), 4.19 (s, 2H), 3.92 (m, 2H), 3.27 (m, 2H), 3.07 (t, *J* = 7.5 Hz, 2H), 2.93 (t, *J* = 7.5 Hz, 2H). ^13^C NMR: δ 170.9 (C), 167.6 (C=O), 160.9 (C), 158.1 (CH), 135.3 (C), 129.4 (C), 124.6 (CH), 123.8 (CH), 121.1 (CH), 114.1 (C), 113.8 (CH), 111.3 (C), 110.3 (CH), 48.2 (CH_2_), 40.7 (CH_2_), 40.6 (CH_2_), 38.2 (CH_2_), 23.6 (CH_2_). LC-MS: [M + H]^+^ 400, 402 (1.89, 89). HRMS: [M + H]^+^ 400.0757. Calcd for C_18_H_19_^79^BrN_5_O^+^: 400.0768. Δ = −2.8.

*2-(4-(2-(1H-Indol-*3-yl*)ethyl)pyrimidin-*2-yl*)isoxazolidine (****28****)*. Prepared as per the method for **31** from 3-(2-(2-chloropyrimidin-4-yl)ethyl)-1*H*-indole **11** and isoxazolidine hydrochloride. Yield 82%. Off-white solid. ^1^H NMR: δ 10.76 (br s, 1H), 8.37 (d, *J* = 5 Hz, 1H), 7.50 (d, *J* = 8 Hz, 1H), 7.32 (d, *J* = 8 Hz, 1H), 7.11 (d, *J* = 2 Hz, 1H), 7.06 (t, *J* = 8 Hz, 1H), 6.96 (t, *J* = 8 Hz, 1H), 6.89 (d, *J* = 5 Hz, 1H), 3.81 (m, 4H), 3.09 (t, *J* = 8 Hz, 2H), 3.00 (t, *J* = 8 Hz, 2H), 2.14 (m, 2H). ^13^C NMR: δ 171.6 (C), 165.9 (C), 158.3 (CH), 136.7 (C), 127.5 (C), 122.9 (CH), 121.4 (CH), 118.7 (CH), 118.6 (CH), 113.9 (C), 113.8 (CH), 111.8 (CH), 67.2 (CH_2_), 49.6 (CH_2_), 38.2 (CH_2_), 27.8 (CH_2_), 24.2 (CH_2_). LC-MS: [M + H]^+^ 295 (1.70, 100). HRMS: [M + H]^+^ 295.1553. Calcd for C_17_H_19_N_4_O^+^: 295.153. Δ = 0.

*2-(4-(2-(5-Chloro-1H-indol-*3-yl*)ethyl)pyrimidin-*2-yl*)isoxazolidine (****29****)*. Prepared as per the method for **31** from 5-chloro-3-(2-(2-chloropyrimidin-4-yl)ethyl)-1*H*-indole **12** and isoxazolidine hydrochloride. Yield 76%. Colorless solid. ^1^H NMR: δ 10.98 (br s, 1H), 8.37 (d, *J* = 5 Hz, 1H), 7.49 (d, *J* = 2 Hz, 1H), 7.33 (d, *J* = 8.5 Hz, 1H), 7.20 (d, *J* = 2 Hz, 1H), 7.05 (dd, *J* = 8.5, 2 Hz, 1H), 6.89 (d, *J* = 5 Hz, 1H), 3.81 (m, 4H), 3.07 (t, *J* = 7.5 Hz, 2H), 2.98 (t, *J* = 7.5 Hz, 2H), 2.14 (m, 2H). ^13^C NMR: δ 171.4 (C), 165.9 (C), 158.2 (CH), 135.1 (C), 128.7 (C), 124.9 (CH), 123.4 (C), 121.3 (CH), 118.0 (CH), 114.0 (C), 113.9 (CH), 113.3 (CH), 67.2 (CH_2_), 49.6 (CH_2_), 38.2 (CH_2_), 27.8 (CH_2_), 23.9 (CH_2_). LC-MS: [M + H]^+^ 329, 331 (1.85, 100). HRMS: [M + H]^+^ 329.1159. Calcd for C_17_H_18_^35^ClN_4_O^+^: 329.1163. Δ = −1.3.

*2-(4-(2-(5-Bromo-1H-indol-*3-yl*)ethyl)pyrimidin-*2-yl*)isoxazolidine (****30****)*. Prepared as per the method for **31** from 5-bromo-3-(2-(2-chloropyrimidin-4-yl)ethyl)-1*H*-indole **13** and isoxazolidine hydrochloride. Yield 73%. Off-white solid. ^1^H NMR: δ 10.99 (br s, 1H), 8.37 (d, *J* = 5 Hz, 1H), 7.63 (d, *J* = 2 Hz, 1H), 7.29 (d, *J* = 8.5 Hz, 1H), 7.18 (d, *J* = 2 Hz, 1H), 7.15 (dd, *J* = 8.5, 2 Hz, 1H), 6.89 (d, *J* = 5 Hz, 1H), 3.81 (m, 4H), 3.07 (t, *J* = 7 Hz, 2H), 2.98 (t, *J* = 7 Hz, 2H), 2.14 (m, 2H). ^13^C NMR: δ 171.4 (C), 165.9 (C), 158.2 (CH), 135.3 (C), 129.4 (C), 124.7 (CH), 123.8 (CH), 121.0 (CH), 113.9 (CH), 113.8 (CH), 111.4 (C), 67.2 (CH_2_), 49.6 (CH_2_), 38.2 (CH_2_), 27.8 (CH_2_), 23.9 (CH_2_). LC-MS: [M + H]^+^ 373, 375 (1.88, 100). HRMS: [M + Na]^+^ 395.0473. Calcd for C_17_H_17_^79^BrN_4_ONa^+^: 395.0478. Δ = −1.3.

*3-(2-(2-(3-Methoxyazetidin-*1-yl*)pyrimidin-*4-yl*)ethyl)-1H-indole (****31****)*. A mixture of 3-(2-(2-chloropyrimidin-4-yl)ethyl)-1*H*-indole **11** (103 mg, 0.4 mmol), 3-methoxyazetidine hydrochloride (123 mg, 1.0 mmol) and anhydrous sodium carbonate (318 mg, 3.0 mmol) in ethanol (3 mL) was stirred and held at reflux for 8 h and allowed to cool to room temperature. The solvent was removed *in vacuo* and the residues partitioned between dichloromethane and water. The organic layer was separated, the solvent removed *in vacuo* and the residues subjected to column chromatography on silica. Elution with 0–100% ethyl acetate in petroleum ether (b.p. 40–60 °C) afforded 3-(2-(2-(3-methoxyazetidin-1-yl)pyrimidin-4-yl)ethyl)-1*H*-indole **31** (85 mg, 69%) as an off-white solid. ^1^H NMR: δ 10.76 (br s, 1H), 8.20 (d, *J* = 5 Hz, 1H), 7.52 (d, *J* = 8 Hz, 1H), 7.32 (d, *J* = 8 Hz, 1H), 7.12 (d, *J* = 2 Hz, 1H), 7.06 (t, *J* = 8 Hz, 1H), 6.97 (t, *J* = 8 Hz, 1H), 6.62 (d, *J* = 5 Hz, 1H), 4.30 (m, 1H), 4.21 (dd, *J* = 9.5, 6 Hz, 2H), 3.83 (dd, *J* = 9.5, 4 Hz, 2H), 3.25 (s, 3H), 3.05 (t, *J* = 8 Hz, 2H), 2.91 (t, *J* = 8 Hz, 2H). ^13^C NMR: δ 171.0 (C), 163.3 (C), 158.0 (CH), 136.7 (C), 127.5 (C), 122.8 (CH), 121.4 (CH), 18.7 (CH), 118.6 (CH), 114.1 (C), 111.8 (CH), 110.1 (CH), 69.9 (CH), 57.3 (CH_2_), 55.8 (CH_3_), 38.1 (CH_2_), 24.1 (CH_2_). LC-MS: [M + H]^+^ 309 (1.71, 100). HRMS: [M + H]^+^ 309.1720. Calcd for C_18_H_21_N_4_O^+^: 309.1710. Δ = +3.2.

*5-Chloro-3-(2-(2-(3-methoxyazetidin-*1-yl*)pyrimidin-*4-yl*)ethyl)-1H-indole (****32****)*. Prepared as per the method for **31** from 5-chloro-3-(2-(2-chloropyrimidin-4-yl)ethyl)-1*H*-indole **12** and 3-methoxyazetidine hydrochloride. Yield 72%. Pale yellow solid. ^1^H NMR: δ 10.97 (br s, 1H), 8.19 (d, *J* = 5 Hz, 1H), 7.53 (d, *J* = 2 Hz, 1H), 7.33 (d, *J* = 8.5 Hz, 1H), 7.20 (d, *J* = 2 Hz, 1H), 7.05 (dd, *J* = 8.5, 2 Hz, 1H), 6.61 (d, *J* = 5 Hz, 1H), 4.29 (m, 1H), 4.20 (dd, *J* = 9.5, 6.5 Hz, 2H), 3.83 (dd, *J* = 9.5, 3.5 Hz, 2H), 3.26 (s, 3H), 3.03 (t, *J* = 8 Hz, 2H), 2.89 (t, *J* = 8 Hz, 2H). ^13^C NMR: δ 170.9 (C), 163.3 (C), 158.0 (CH), 135.1 (C), 128.7 (C), 124.8 (CH), 123.4 (C), 121.3 (CH), 118.1 (CH), 114.2 (C), 113.3 (CH), 110.2 (CH), 69.9 (CH), 57.3 (CH_2_), 55.8 (CH_3_), 38.0 (CH_2_), 23.7 (CH_2_). LC-MS: [M + H]^+^ 343, 345 (1.90, 100). HRMS: [M + H]^+^ 343.1312. Calcd for C_18_H_20_^35^ClN_4_O^+^: 343.1320. Δ = −2.4.

*5-Bromo-3-(2-(2-(3-methoxyazetidin-*1-yl*)pyrimidin-*4-yl*)ethyl)-1H-indole (****33****)*. Prepared as per the method for **31** from 5-bromo-3-(2-(2-chloropyrimidin-4-yl)ethyl)-1*H*-indole **13** and 3-methoxyazetidine hydrochloride. Yield 82%. Off-white solid. ^1^H NMR: δ 10.99 (br s, 1H), 8.19 (d, *J* = 5 Hz, 1H), 7.66 (d, *J* = 2 Hz, 1H), 7.29 (d, *J* = 8.5 Hz, 1H), 7.19 (d, *J* = 2 Hz, 1H), 7.16 (dd, *J* = 8.5, 2 Hz, 1H), 6.61 (d, *J* = 5 Hz, 1H), 4.30 (m, 1H), 4.20 (dd, *J* = 9, 6.5 Hz, 2H), 3.83 (dd, *J* = 9, 4 Hz, 2H), 3.25 (s, 3H), 3.03 (t, *J* = 7 Hz, 2H), 2.89 (t, *J* = 8 Hz, 2H). ^13^C NMR: δ 170.9 (C), 163.3 (C), 157.9 (CH), 135.3 (C), 129.5 (C), 124.6 (CH), 123.8 (CH), 121.1 (CH), 114.1 (C), 113.8 (CH), 111.3 (C), 110.2 (CH), 69.9 (CH), 57.3 (CH_2_), 55.8 (CH_3_), 38.1 (CH_2_), 23.7 (CH_2_). LC-MS: [M + H]^+^ 387, 389 (1.94, 100). HRMS: [M + H]^+^ 387.0802. Calcd for C_18_H_20_^79^BrN_4_O^+^: 387.0815. Δ = −3.4.

*3-(2-(2-(4-(Trifluoromethoxy)phenyl)pyrimidin-*4-yl*)ethyl)-1H-indole (****34****)*. A mixture of 3-(2-(2-chloropyrimidin-4-yl)ethyl)-1*H*-indole **11** (103 mg, 0.4 mmol), (4-(trifluoromethoxy)phenyl)-boronic acid (124 mg, 0.6 mmol), [1,1′-bis(diphenylphosphino)ferrocene]-dichloropalladium(II) (59 mg, 0.08 mmol) and anhydrous sodium carbonate (212 mg, 2.0 mmol) in water (0.5 mL), ethanol (1 mL) and toluene (2 mL) was degassed with nitrogen for 5 min and stirred and held at 90 °C in a sealed tube for 6 h and allowed to cool to room temperature. The mixture was diluted with ethyl acetate and water, the organic layer was separated, the solvent removed *in vacuo* and the residues subjected to column chromatography on silica. Elution with 0–50% ethyl acetate in petroleum ether (b.p. 40–60 °C) afforded 3-(2-(2-(4-(trifluoromethoxy)phenyl)pyrimidin-4-yl)ethyl)-1*H*-indole **34** (145 mg, 95%) as a pale yellow solid. ^1^H NMR: δ 10.77 (br s, 1H), 8.76 (d, *J* = 5 Hz, 1H), 8.53 (d, *J* = 9 Hz, 2H), 7.57 (d, *J* = 8 Hz, 1H), 7.52 (dq, *J* = 9, 1 Hz, 2H), 7.37 (d, *J* = 5 Hz, 1H), 7.33 (d, *J* = 8 Hz, 1H), 7.14 (d, *J* = 2 Hz, 1H), 7.07 (t, *J* = 8 Hz, 1H), 6.97 (t, *J* = 8 Hz, 1H), 3.21 (s, 4H). ^13^C NMR: δ 171.0 (C), 162.2 (C), 157.9 (CH), 150.6 (q, *J* = 2 Hz, COCF_3_), 137.0 (C), 136.7 (C), 130.2 (CH), 127.5 (C), 122.9 (CH), 121.5 (CH), 121.4 (CH), 120.5 (q, *J* = 257 Hz, OCF_3_), 119.6 (CH), 118.8 (CH), 118.7 (CH), 113.9 (C), 111.8 (CH), 38.2 (CH_2_), 24.1 (CH_2_). ^19^F NMR: δ −56.6 (OCF_3_). LC-MS: [M + H]^+^ 384 (2.53, 100). HRMS: [M − H]^-^ 382.1164. Calcd for C_21_H_15_F_3_N_3_O^−^: 382.1172. Δ = −2.1.

*2-Chloro-3-(2-(2-(4-(trifluoromethoxy)phenyl)pyrimidin-*4-yl*)ethyl)-1H-indole (****35****)*. A stirred solution of 3-(2-(2-(4-(trifluoromethoxy)phenyl)pyrimidin-4-yl)ethyl)-1*H*-indole **34** (38.3 mg, 0.1 mmol) in tetrahydrofuran (2 mL) was treated with *N*-chlorosuccinimide (13.4 mg, 0.1 mmol) and the resulting mixture was stirred and held at reflux for 5 h and allowed to cool to room temperature. The solvent was removed *in vacuo* and the residues partitioned between dichloromethane and water. The organic layer was separated, the solvent removed *in vacuo* and the residues subjected to column chromatography on silica. Elution with 0–30% ethyl acetate in petroleum ether (b.p. 40–60 °C) afforded 2-chloro-3-(2-(2-(4-(trifluoromethoxy)phenyl)-pyrimidin-4-yl)ethyl)-1*H*-indole **35** (24 mg, 57%) as a pale orange solid. ^1^H NMR: δ 11.59 (br s, 1H), 8.73 (d, *J* = 5 Hz, 1H), 8.49 (d, *J* = 9 Hz, 2H), 7.52 (dq, *J* = 9, 1 Hz, 2H), 7.51 (d, *J* = 8 Hz, 1H), 7.26 (d, *J* = 8 Hz, 1H), 7.25 (d, *J* = 5 Hz, 1H), 7.10 (t, *J* = 8 Hz, 1H), 7.00 (t, *J* = 8 Hz, 1H), 3.19 (t, *J* = 7 Hz, 2H), 3.12 (t, *J* = 7 Hz, 2H). ^13^C NMR: δ 170.3 (C), 162.2 (C), 157.9 (CH), 150.6 (q, *J* = 2 Hz, COCF_3_), 137.0 (C), 135.0 (C), 130.2 (CH), 127.2 (C), 122.0 (CH), 121.4 (CH), 121.1 (C), 120.5 (q, *J* = 256 Hz, OCF_3_), 119.7 (CH), 119.6 (CH), 118.4 (CH), 111.3 (CH), 109.6 (C), 37.6 (CH_2_), 23.0 (CH_2_). ^19^F NMR: δ −56.6 (OCF_3_). LC-MS: [M + H]^+^ 418, 420 (2.60, 100). HRMS: [M − H]^-^ 416.0781. Calcd for C_21_H_14_^35^ClF_3_N_3_O^−^: 416.0783. Δ = −0.5.

*2-Bromo-3-(2-(2-(4-(trifluoromethoxy)phenyl)pyrimidin-*4-yl*)ethyl)-1H-indole (****36****)*. A stirred solution of 3-(2-(2-(4-(trifluoromethoxy)phenyl)pyrimidin-4-yl)ethyl)-1*H*-indole **34** (38.3 mg, 0.1 mmol) in dichloromethane (2 mL) was treated with *N*-bromosuccinimide (17.8 mg, 0.1 mmol) and the resulting mixture was stirred and held at room temperature for 4 h. The mixture was diluted with dichloromethane and water, the organic layer was separated, the solvent removed *in vacuo* and the residues subjected to column chromatography on silica. Elution with 0–30% ethyl acetate in petroleum ether (b.p. 40–60 °C) afforded 2-bromo-3-(2-(2-(4-(trifluoromethoxy)phenyl)pyrimidin-4-yl)ethyl)-1*H*-indole **36** (28 mg, 61%) as a pale pink solid. ^1^H NMR: δ 11.59 (br s, 1H), 8.73 (d, *J* = 5 Hz, 1H), 8.50 (d, *J* = 9 Hz, 2H), 7.52 (dq, *J* = 9, 1 Hz, 2H), 7.51 (d, *J* = 8 Hz, 1H), 7.27 (d, *J* = 8 Hz, 1H), 7.24 (d, *J* = 5 Hz, 1H), 7.08 (t, *J* = 8 Hz, 1H), 6.98 (t, *J* = 8 Hz, 1H), 3.18 (t, *J* = 7 Hz, 2H), 3.11 (t, *J* = 7 Hz, 2H). ^13^C NMR: δ 170.3 (C), 162.2 (C), 157.9 (CH), 150.6 (q, *J* = 1.5 Hz, COCF_3_), 137.0 (C), 136.6 (C), 130.3 (CH), 127.4 (C), 122.0 (CH), 121.4 (CH), 120.5 (q, *J* = 257 Hz, OCF_3_), 119.7 (CH), 119.6 (CH), 118.3 (CH), 112.8 (C), 111.2 (CH), 109.1 (C), 37.8 (CH_2_), 24.0 (CH_2_). ^19^F NMR: δ −56.6 (OCF_3_). LC-MS: [M + H]^+^ 462, 464 (2.61, 100). HRMS: [M − H]^-^ 460.0270. Calcd for C_21_H_14_^79^BrF_3_N_3_O^−^: 460.0278. Δ = −1.8.

*3-(2-(2-(Pyridin-*2-yl*)pyrimidin-*4-yl*)ethyl)-1H-indole hydrochloride (****37****)*. A mixture of 3-(2-(2-chloropyrimidin-4-yl)ethyl)-1*H*-indole **11** (117 mg, 0.45 mmol), 4-methyl-8-(pyridin-2-yl)dihydro-4λ [4],8λ [4]-[1,3,2]oxazaborolo[2,3-*b*][1,3,2]oxazaborole-2,6(3*H*,5*H*)-dione (176 mg, 0.75 mmol), tris(dibenzylideneacetone)dipalladium(0) (13.8 mg, 0.015 mmol), 2-dicyclohexylphosphino-2′,4′,6′-triisopropylbiphenyl (XPhos) (35.7 mg, 0.075 mmol), copper(II) acetate (40 mg, 0.22 mmol) and anhydrous potassium carbonate (414 mg, 3.0 mmol) in *N*,*N*-dimethylformamide (3 mL) and isopropanol (0.8 mL) [17] was degassed with nitrogen for 5 min and stirred and held at 100 °C in a sealed tube for 6 h and allowed to cool to room temperature. The mixture was diluted with ethyl acetate and 1 M aqueous sodium hydroxide, the organic layer was separated and the solvent removed *in vacuo*. The crude material was suspended in ethyl acetate (2 mL) and treated with di-*tert*-butyl dicarbonate (0.2 mL), triethylamine (0.2 mL) and *N*,*N*-dimethylpyridin-4-amine (a few crystals) and the mixture was stirred and held at room temperature for 2 h. The solvent was removed *in vacuo* and the residues partitioned between dichloromethane and water. The organic layer was separated, the solvent removed *in vacuo* and the residues subjected to column chromatography on silica. Elution with 0–100% ethyl acetate in petroleum ether (b.p. 40–60 °C) afforded *tert*-butyl 3-(2-(2-(pyridin-2-yl)pyrimidin-4-yl)ethyl)-1*H*-indole-1-carboxylate **38** (42 mg, 23%) as a pale tan foam. ^1^H NMR: δ 8.84 (d, *J* = 5 Hz, 1H), 8.77 (ddd, *J* = 4.5, 2, 1 Hz, 1H), 8.38 (d, *J* = 8 Hz, 1H), 8.03 (d, *J* = 8 Hz, 1H), 7.98 (td, *J* = 7.5, 2 Hz, 1H), 7.68 (dt, *J* = 7.5, 1 Hz, 1H), 7.54 (ddd, *J* = 7.5, 4.5, 1 Hz, 1H), 7.51 (d, *J* = 5 Hz, 1H), 7.49 (s, 1H), 7.33 (t, *J* = 8 Hz, 1H), 7.24 (t, *J* = 8 Hz, 1H), 3.23 (s, 4H), 1.59 (s, 9H). ^13^C NMR: δ 170.5 (C), 163.4 (C), 158.0 (CH), 155.3 (C), 150.1 (CH), 149.5 (C=O), 137.5 (CH), 135.3 (C), 130.6 (C), 125.4 (CH), 124.9 (CH), 123.9 (CH), 123.3 (CH), 123.0 (CH), 120.3 (CH), 119.8 (CH), 115.2 (CH), 104.0 (C), 83.9 (C), 37.0 (CH_2_), 28.1 (CH_3_), 23.4 (CH_2_). LC-MS: [M + H]^+^ 401 (2.24, 97). HRMS: [M + H]^+^ 401.1972. Calcd for C_24_H_25_N_4_O_2_^+^: 401.1972. Δ = 0.

Hydrogen chloride (2 M in diethyl ether) (0.5 mL) was added to a stirred solution of *tert*-butyl 3-(2-(2-(pyridin-2-yl)pyrimidin-4-yl)ethyl)-1*H*-indole-1-carboxylate **38** (24 mg, 0.06 mmol) in diethyl ether (2 mL) and methanol (1 mL) and the mixture was stirred and held at 45 °C for 3 h and allowed to cool to room temperature. The solvent was removed *in vacuo* and the residues triturated with diethyl ether to afford 3-(2-(2-(pyridin-2-yl)pyrimidin-4-yl)ethyl)-1*H*-indole hydrochloride **37** (20 mg, 99%) as a brown solid. ^1^H NMR: δ 10.82 (br s, 1H), 8.95 (d, *J* = 5 Hz, 1H), 8.92 (ddd, *J* = 4.5, 2, 1 Hz, 1H), 8.77 (d, *J* = 8 Hz, 1H), 8.54 (td, *J* = 7.5, 2 Hz, 1H), 8.01 (ddd, *J* = 7.5, 4.5, 1 Hz, 1H), 7.64 (d, *J* = 5 Hz, 1H), 7.58 (d, *J* = 8 Hz, 1H), 7.34 (dt, *J* = 7.5, 1 Hz, 1H), 7.16 (d, *J* = 2 Hz, 1H), 7.07 (t, *J* = 8 Hz, 1H), 6.98 (t, *J* = 8 Hz, 1H), 3.28 (m, 4H). ^13^C NMR: δ 172.3 (C), 158.2 (CH), 157.7 (C), 148.9 (C), 145.7 (CH), 145.2 (CH), 136.7 (C), 128.4 (CH), 127.5 (C), 125.7 (CH), 123.1 (CH), 122.4 (CH), 121.4 (CH), 118.8 (CH), 118.7 (CH), 113.6 (C), 111.9 (CH), 38.1 (CH_2_), 24.1 (CH_2_). LC-MS: [M + H]^+^ 301 (1.56, 100). HRMS: [M + Na]^+^ 323.1271. Calcd for C_19_H_16_N_4_Na^+^: 323.1267. Δ = +1.2.

*5-Fluoro-3-(2-(pyridin-*2-yl*)ethyl)-1H-indole (****39****)*. Prepared as per the method for **8** [13] from 2-vinylpyridine and 5-fluoro-1*H*-indole. Yield 23%. Colorless solid. ^1^H NMR: δ 10.85 (br s, 1H), 8.51 (ddd, *J* = 5, 2, 1 Hz, 1H), 7.67 (td, *J* = 7.5, 2 Hz, 1H), 7.31 (dd, *J* = 9, 4.5 Hz, 1H), 7.28 (dt, *J* = 7.5, 1 Hz, 1H), 7.23 (dd, *J* = 10, 2.5 Hz, 1H), 7.20 (ddd, *J* = 7.5, 5, 1 Hz, 1H), 7.17 (d, *J* = 2 Hz, 1H), 6.89 (td, *J* = 9, 2.5 Hz, 1H), 3.07 (m, 4H). ^13^C NMR: δ 161.8 (C), 157.1 (d, *J* = 230.5 Hz, CF), 149.4 (CH), 136.8 (CH), 133.3 (C), 127.8 (d, *J* = 10 Hz, CCHCF), 124.9 (CH), 123.3 (CH), 121.7 (CH), 114.8 (d, *J* = 5 Hz, CCHCHCF), 112.7 (d, *J* = 9.5 Hz, CHCHCF), 109.4 (d, *J* = 26 Hz, CHCF), 103.4 (d, *J* = 23 Hz, CHCF), 38.6 (CH_2_), 25.2 (CH_2_). ^19^F NMR: δ −125.7. LC-MS: [M + H]^+^ 241 (1.25 100). HRMS: [M + Na]^+^ 263.0953. Calcd for C_15_H_13_FN_2_Na^+^: 263.0955. Δ = −0.8.

*5-Iodo-3-(2-(pyridin-*2-yl*)ethyl)-1H-indole (****40****)*. Prepared as per the method for **8** [13] from 2-vinylpyridine and 5-iodo-1*H*-indole. Yield 22%. Tan solid. ^1^H NMR: δ 10.95 (br s, 1H), 8.51 (ddd, *J* = 5, 2, 1 Hz, 1H), 7.80 (d, *J* = 1.5 Hz, 1H), 7.67 (td, *J* = 7.5, 2 Hz, 1H), 7.30 (dd, *J* = 8.5, 1.5 Hz, 1H), 7.27 (dt, *J* = 7.5, 1 Hz, 1H), 7.20 (ddd, *J* = 7.5, 5, 1 Hz, 1H), 7.19 (d, *J* = 8.5 Hz, 1H), 7.11 (d, *J* = 2 Hz, 1H), 3.06 (s, 4H). ^13^C NMR: δ 161.7 (C), 149.4 (CH), 136.8 (CH), 135.7 (C), 130.4 (C), 129.2 (CH), 127.3 (CH), 124.0 (CH), 123.3 (CH), 122.2 (C), 121.7 (CH), 114.3 (CH), 114.1 (C), 38.8 (CH_2_), 25.1 (CH_2_). LC-MS: [M + H]^+^ 349 (1.48, 100). HRMS: [M + H]^+^ 349.0183. Calcd for C_15_H_14_IN_2_^+^: 349.0196. Δ = −3.8.

*5-Iodo-3-(2-(pyridin-*4-yl*)ethyl)-1H-indole (****42****)*. Prepared as per the method for **8** [13] from 4-vinylpyridine and 5-iodo-1*H*-indole. Yield 40%. Pale yellow solid. ^1^H NMR: δ 10.98 (br s, 1H), 8.44 (d, *J* = 6 Hz, 2H), 7.90 (d, *J* = 1.5, 1H), 7.32 (dd, *J* = 8.5, 1.5 Hz, 1H), 7.28 (d, *J* = 6 Hz, 2H), 7.20 (d, *J* = 8.5 Hz, 1H), 7.11 (d, *J* = 2 Hz, 1H), 2.97 (m, 4H). ^13^C NMR: δ 151.2 (C), 149.8 (CH), 135.7 (C), 130.3 (C), 129.3 (CH), 127.3 (CH), 124.5 (CH), 124.2 (CH), 114.3 (CH), 113.6 (C), 82.5 (C), 35.5 (CH_2_), 25.6 (CH_2_). LC-MS: [M + H]^+^ 349 (1.48, 100). HRMS: [M + H]^+^ 349.0207. Calcd for C_15_H_14_IN_2_^+^: 349.0196. Δ = +3.1.

*3-(2-(Pyridin-*4-yl*)ethyl)-5-(trifluoromethoxy)-1H-indole (****44****)*. Prepared as per the method for **8** [13] from 4-vinylpyridine and 5-(trifluoromethoxy)-1*H*-indole. Yield 52%. Colorless solid. ^1^H NMR: δ 11.08 (br s, 1H), 8.44 (d, *J* = 5 Hz, 2H), 7.51 (br s, 1H), 7.41 (d, *J* = 8.5 Hz, 1H), 7.28 (d, *J* = 5 Hz, 2H), 7.26 (d, *J* = 2 Hz, 1H), 7.04 (br d, *J* = 8.5 Hz, 1H), 3.02 (m, 2H), 2.98 (m, 2H). ^13^C NMR: δ 151.2 (C), 149.8 (CH), 141.8 (q, *J* = 2 Hz, COCF_3_), 135.1 (C), 127.6 (C), 125.5 (CH), 124.5 (CH), 115.0 (CH), 112.8 (CH), 114.7 (C), 111.3 (CH), 120.9 (q, *J* = 253 Hz, OCF_3_), 35.5 (CH_2_), 25.7 (CH_2_). ^19^F NMR: δ −56.8. LC-MS: [M + H]^+^ 307 (1.55, 100). HRMS: [M + H]^+^ 307.1061. Calcd for C_16_H_14_F_3_N_2_O^+^: 307.1053. Δ = +2.6.

*3-(2-(2-Chloropyrimidin-*4-yl*)ethyl)-5-fluoro-1H-indole: (****46****)*. Prepared as per the method for **11** [13] from 2-chloro-4-vinylpyrimidine [16] and 5-fluoro-1*H*-indole. Yield 32%. Yellow solid. ^1^H NMR: δ 10.90 (br s, 1H), 8.62 (d, *J* = 5 Hz, 1H), 7.48 (d, *J* = 5 Hz, 1H), 7.32 (dd, *J* = 9, 4.5 Hz, 1H), 7.30 (dd, *J* = 10, 2.5 Hz, 1H), 7.20 (d, *J* = 1.5 Hz, 1H), 6.90 (td, *J* = 9, 2.5 Hz, 1H), 3.10 (m, 4H). ^13^C NMR: δ 174.6 (C), 160.4 (CH), 160.3 (C), 157.1 (d, *J* = 231 Hz, CF), 133.3 (C), 127.6 (d, *J* = 10 Hz, CCHCF), 125.2 (CH), 120.4 (CH), 113.8 (d, *J* = 5 Hz, CCHCHCF), 112.7 (d, *J* = 10 Hz, CHCHCF), 109.5 (d, *J* = 26 Hz, CHCF), 103.5 (d, *J* = 23 Hz, CHCF), 37.7 (CH_2_), 23.8 (CH_2_). ^19^F NMR: δ −125.5. LC-MS: [M − H]^-^ 274, 276 (1.94, 100). HRMS: [M − H]^-^ 274.0544. Calcd for C_14_H_10_^35^ClFN_3_^−^: 274.0553. Δ = −3.3.

*3-(2-(2-Chloropyrimidin-*4-yl*)ethyl)-5-methoxy-1H-indole (****47****)*. Prepared as per the method for **11** [13] from 2-chloro-4-vinylpyrimidine [16] and 5-methoxy-1*H*-indole. Yield 32%. Pale yellow solid. ^1^H NMR: δ 10.63 (br s, 1H), 8.61 (d, *J* = 5 Hz, 1H), 7.47 (d, *J* = 5 Hz, 1H), 7.21 (d, *J* = 8.5 Hz, 1H), 7.08 (d, *J* = 2.5 Hz, 1H), 6.96 (d, *J* = 2.5 Hz, 1H), 6.71 (dd, *J* = 8.5, 2.5 Hz, 1H), 3.76 (s, 3H), 3.10 (s, 4H). ^13^C NMR: δ 174.8 (C), 160.4 (C), 160.4 (CH), 153.5 (C), 131.8 (C), 127.7 (C), 123.7 (CH), 120.4 (CH), 113.2 (C), 112.5 (CH), 111.6 (CH), 100.4 (CH), 55.8 (CH_3_), 37.8 (CH_2_), 24.0 (CH_2_). LC-MS: [M − H]^-^ 286, 288 (1.83, 100). HRMS: [M + Na]^+^ 310.0718. Calcd for C_15_H_14_^35^ClN_3_ONa^+^: 310.0717. Δ = +0.3.

*3-(2-(2-Chloropyrimidin-*4-yl*)ethyl)-5-(trifluoromethoxy)-1H-indole (****48****)*. Prepared as per the method for **11** [13] from 2-chloro-4-vinylpyrimidine [16] and 5-(trifluoromethoxy)-1*H*-indole. Yield 51%. Yellow solid. ^1^H NMR: δ 11.10 (br s, 1H), 8.62 (d, *J* = 5 Hz, 1H), 7.49 (d, *J* = 5 Hz, 1H), 7.48 (d, *J* = 2.5 Hz, 1H), 7.41 (d, *J* = 8.5 Hz, 1H), 7.28 (d, *J* = 2.5 Hz, 1H), 7.04 (dd, *J* = 8.5, 2.5 Hz, 1H), 3.12 (s, 4H). ^13^C NMR: δ 174.5 (C), 160.4 (CH), 160.4 (C), 141.9 (q, *J* = 1.5 Hz, COCF_3_), 135.1 (C), 127.5 (C), 125.6 (CH), 120.9 (q, *J* = 254 Hz, OCF_3_), 120.4 (CH), 115.0 (CH), 114.3 (C), 112.9 (CH), 111.2 (CH), 37.8 (CH_2_), 23.7 (CH_2_). ^19^F NMR: δ −56.8. LC-MS: [M − H]^-^ 340, 342 (2.14, 97). HRMS: [M − H]^-^ 340.0457. Calcd for C_15_H_10_^35^ClF_3_N_3_O^−^: 340.0470. Δ = −3.9.

*5-(Benzyloxy)-3-(2-(2-chloropyrimidin-*4-yl*)ethyl)-1H-indole (****49****)*. Prepared as per the method for **11** [13] from 2-chloro-4-vinylpyrimidine [16] and 5-(benzyloxy)-1*H*-indole. Yield 50%. Pale yellow solid. ^1^H NMR: δ 10.66 (br s, 1H), 8.62 (d, *J* = 5 Hz, 1H), 7.49 (d, *J* = 7 Hz, 2H), 7.46 (d, *J* = 5 Hz, 1H), 7.40 (t, *J* = 7 Hz, 2H), 7.33 (t, *J* = 7 Hz, 1H), 7.22 (d, *J* = 8.5 Hz, 1H), 7.10 (d, *J* = 2.5 Hz, 1H), 7.09 (d, *J* = 2.5 Hz, 1H), 6.79 (dd, *J* = 8.5, 2.5 Hz, 1H), 5.09 (s, 2H), 3.10 (m, 4H). ^13^C NMR: δ 174.9 (C), 160.4 (CH), 160.4 (C), 152.5 (C), 138.3 (C), 132.0 (C), 128.8 (CH), 128.1 (CH), 128.0 (CH), 127.7 (C), 123.8 (CH), 120.4 (CH), 113.2 (C), 112.5 (CH), 112.2 (CH), 102.1 (CH), 70.3 (CH_2_), 37.8 (CH_2_), 24.1 (CH_2_). LC-MS: [M + H]^+^ 364, 366 (2.20, 100). HRMS: [M + Na]^+^ 386.1031. Calcd for C_21_H_18_^35^ClN_3_ONa^+^: 386.1031. Δ = 0.

*3-(2-(2-Chloropyrimidin-*4-yl*)ethyl)-1-methyl-1H-indole (****50****)*. Prepared as per the method for **11** [13] from 2-chloro-4-vinylpyrimidine [16] and 1-methyl-1*H*-indole. Yield 48%. Orange oil. ^1^H NMR: δ 8.63 (d, *J* = 5 Hz, 1H), 7.55 (d, *J* = 8, 1H), 7.49 (d, *J* = 5 Hz, 1H), 7.37 (d, *J* = 8 Hz, 1H), 7.14 (t, *J* = 8 Hz, 1H), 7.11 (s, 1H), 7.01 (t, *J* = 8 Hz, 1H), 3.72 (s, 3H), 3.12 (s, 4H). ^13^C NMR: δ 174.6 (C), 160.4 (CH), 160.4 (C), 137.1 (C), 127.7 (C), 127.4 (CH), 121.6 (CH), 120.3 (CH), 118.9 (CH), 118.8 (CH), 112.8 (C), 110.1 (CH), 37.9 (CH_2_), 32.7 (CH_3_), 23.8 (CH_2_). LC-MS: [M + H]^+^ 272, 274 (2.11, 97). HRMS: [M + Na]^+^ 294.0767. Calcd for C_15_H_14_^35^ClN_3_Na^+^: 294.0769. Δ = −0.7.

*4-(4-(2-(5-Fluoro-1H-indol-*3-yl*)ethyl)pyrimidin-*2-yl*)morpholine (****51****)*. Prepared as per the method for **14** from 3-(2-(2-chloropyrimidin-4-yl)ethyl)-5-fluoro-1*H*-indole **46** and morpholine. Yield 83%. Pale yellow solid. ^1^H NMR: δ 10.86 (br s, 1H), 8.23 (d, *J* = 5 Hz, 1H), 7.31 (dd, *J* = 9, 4.5 Hz, 1H), 7.26 (dd, *J* = 10, 2.5 Hz, 1H), 7.20 (d, *J* = 2 Hz, 1H), 6.89 (td, *J* = 9, 2.5 Hz, 1H), 6.60 (d, *J* = 5 Hz, 1H), 3.70 (m, 4H), 3.65 (m, 4H), 3.05 (t, *J* = 7.5 Hz, 2H), 3.05 (t, *J* = 7.5 Hz, 2H). ^13^C NMR: δ 170.8 (C), 161.8 (C), 158.0 (CH), 157.1 (d, *J* = 230.5 Hz, CF), 133.3 (C), 127.8 (d, *J* = 9.5 Hz, CCHCF), 125.0 (CH), 114.5 (d, *J* = 5 Hz, CCHCHCF), 112.7 (d, *J* = 9.5 Hz, CHCHCF), 110.1 (CH), 109.4 (d, *J* = 26 Hz, CHCF), 103.4 (d, *J* = 23 Hz, CHCF), 66.5 (CH_2_), 44.3 (CH_2_), 38.1 (CH_2_), 23.8 (CH_2_). ^19^F NMR: δ −125.7. LC-MS: [M + H]^+^ 327 (1.96, 100). HRMS: [M + H]^+^ 327.1618. Calcd for C_18_H_20_FN_4_O^+^: 327.1616. Δ = +0.6.

*4-(4-(2-(5-Methoxy-1H-indol-*3-yl*)ethyl)pyrimidin-*2-yl*)morpholine (****52****)*. Prepared as per the method for **14** from 3-(2-(2-chloropyrimidin-4-yl)ethyl)-5-methoxy-1*H*-indole **47** and morpholine. Yield 81%. Yellow solid. ^1^H NMR: δ 10.59 (br s, 1H), 8.23 (d, *J* = 5 Hz, 1H), 7.21 (d, *J* = 8.5 Hz, 1H), 7.07 (d, *J* = 2 Hz, 1H), 6.94 (d, *J* = 2 Hz, 1H), 6.70 (dd, *J* = 8.5, 2 Hz, 1H), 6.60 (d, *J* = 5 Hz, 1H), 3.75 (s, 3H), 3.70 (m, 4H), 3.65 (m, 4H), 3.05 (t, *J* = 7.5 Hz, 2H), 2.92 (t, *J* = 7.5 Hz, 2H). ^13^C NMR: δ 171.1 (C), 161.9 (C), 158.0 (CH), 153.4 (C), 131.9 (C), 127.9 (C), 123.5 (CH), 114.0 (C), 112.4 (CH), 111.5 (CH), 110.1 (CH), 100.6 (CH), 66.5 (CH_2_), 55.8 (CH_3_), 44.3 (CH_2_), 38.2 (CH_2_), 24.0 (CH_2_). LC-MS: [M + H]^+^ 339 (1.84, 100). HRMS: [M + H]^+^ 339.1810. Calcd for C_19_H_23_N_4_O_2_^+^: 339.1815. Δ = −1.5.

*4-(4-(2-(5-(Trifluoromethoxy)-1H-indol-*3-yl*)ethyl)pyrimidin-*2-yl*)morpholine (****53****)*. Prepared as per the method for **14** from 3-(2-(2-chloropyrimidin-4-yl)ethyl)-5-(trifluoromethoxy)-1*H*-indole **48** and morpholine. Yield 77%. Pale yellow solid. ^1^H NMR: δ 11.06 (br s, 1H), 8.22 (d, *J* = 5 Hz, 1H), 7.44 (d, *J* = 2 Hz, 1H), 7.40 (d, *J* = 9 Hz, 1H), 7.27 (d, *J* = 2 Hz, 1H), 7.02 (dd, *J* = 9, 2 Hz, 1H), 6.59 (d, *J* = 5 Hz, 1H), 3.69 (m, 4H), 3.65 (m, 4H), 3.08 (t, *J* = 7.5 Hz, 2H), 2.92 (t, *J* = 7.5 Hz, 2H). ^13^C NMR: δ 170.7 (C), 161.8 (C), 158.0 (CH), 141.8 (q, *J* = 2 Hz, COCF_3_), 135.1 (C), 127.7 (C), 125.4 (CH), 120.9 (q, *J* = 254 Hz, OCF_3_), 115.0 (C), 114.9 (CH), 112.8 (CH), 111.2 (CH), 110.1 (CH), 66.5 (CH_2_), 44.3 (CH_2_), 38.2 (CH_2_), 23.6 (CH_2_). ^19^F NMR: δ −56.8. LC-MS: [M + H]^+^ 393 (2.19, 100). HRMS: [M + H]^+^ 393.1534. Calcd for C_19_H_20_F_3_N_4_O_2_^+^: 393.1533. Δ = +0.2.

*4-(4-(2-(5-(Benzyloxy)-1H-indol-*3-yl*)ethyl)pyrimidin-*2-yl*)morpholine (****54****)*. Prepared as per the method for **14** from 5-(benzyloxy)-3-(2-(2-chloropyrimidin-4-yl)ethyl)-1*H*-indole **44** and morpholine. Yield 72%. Off-white solid. ^1^H NMR: δ 10.62 (br s, 1H), 8.24 (d, *J* = 5 Hz, 1H), 7.48 (d, *J* = 7 Hz, 2H), 7.40 (t, *J* = 7 Hz, 2H), 7.32 (d, *J* = 7 Hz, 1H), 7.22 (d, *J* = 8 Hz, 1H), 7.08 (d, *J* = 2 Hz, 1H), 7.06 (d, *J* = 2.5 Hz, 1H), 6.78 (dd, *J* = 8.5, 2.5 Hz, 1H), 6.59 (d, *J* = 5 Hz, 1H), 5.07 (s, 2H), 3.70 (m, 4H), 3.64 (m, 4H), 3.03 (t, *J* = 8 Hz, 2H), 2.90 (t, *J* = 8 Hz, 2H). ^13^C NMR: δ 171.0 (C), 161.8 (C), 158.0 (CH), 152.4 (C), 138.3 (C), 132.0 (C), 128.8 (CH), 128.1 (CH), 128.0 (CH), 127.8 (C), 123.6 (CH), 114.0 (C), 112.4 (CH), 112.1 (CH), 110.1 (CH), 102.2 (CH), 70.3 (CH_2_), 66.5 (CH_2_), 44.3 (CH_2_), 38.3 (CH_2_), 24.0 (CH_2_). LC-MS: [M + H]^+^ 415 (2.24, 100). HRMS: [M + H]^+^ 415.2133. Calcd for C_25_H_27_N_4_O_2_^+^: 415.2129. Δ = +0.9.

*3-(2-(2-Morpholinopyrimidin-*4-yl*)ethyl)-1H-indol-5-ol (****55****)*. A mixture of 4-(4-(2-(5-(benzyloxy)-1*H*-indol-3-yl)ethyl)pyrimidin-2-yl)morpholine **54** (207 mg, 0.5 mmol), 10% palladium on carbon (50 mg) in methanol (5 mL) and water (0.5 mL) was degassed with nitrogen for 5 min and then stirred and held at room temperature under an atmosphere of hydrogen gas for 2 h. The mixture was filtered, the catalyst rinsed with methanol (5 mL) and the organic solvent removed *in vacuo*. The residues were partitioned between dichloromethane and water, the organic layer separated, and the solvent removed *in vacuo* to afford 3-(2-(2-morpholinopyrimidin-4-yl)ethyl)-1*H*-indol-5-ol **55** (140 mg, 86%) as a colorless foam. ^1^H NMR: δ 10.44 (br s, 1H), 8.59 (br s, 1H), 8.25 (d, *J* = 5 Hz, 1H), 7.11 (d, *J* = 8.5 Hz, 1H), 7.01 (d, *J* = 2 Hz, 1H), 6.83 (d, *J* = 2 Hz, 1H), 6.60 (d, *J* = 5 Hz, 1H), 6.59 (dd, *J* = 8.5, 2 Hz, 1H), 3.71 (m, 4H), 3.66 (m, 4H), 2.98 (t, *J* = 7.5 Hz, 2H), 2.89 (t, *J* = 7.5 Hz, 2H). ^13^C NMR: δ 171.0 (C), 161.8 (C), 158.0 (CH), 150.6 (C), 131.3 (C), 128.2 (C), 123.3 (CH), 113.2 (C), 112.2 (CH), 111.7 (CH), 110.0 (CH), 102.7 (CH), 66.5 (CH_2_), 44.3 (CH_2_), 38.1 (CH_2_), 24.1 (CH_2_). LC-MS: [M + H]^+^ 325 (1.53, 97). HRMS: [M + H]^+^ 325.1658. Calcd for C_18_H_21_N_4_O_2_^+^: 325.1659. Δ = −0.4.

*4-(4-(2-(1H-Indol-3-yl-5-d)ethyl)pyrimidin-*2-yl*)morpholine (****56****)*. A mixture of 4-(4-(2-(5-bromo-1*H*-indol-3-yl)ethyl)pyrimidin-2-yl)morpholine **21** (117 mg, 0.3 mmol), 10% palladium on carbon (50 mg) and 3 M aqueous potassium hydroxide (0.5 mL, 1.5 mmol) in methanol (5 mL) was degassed with nitrogen for 5 min and then stirred and held at room temperature under an atmosphere of deuterium gas for 1 h. The mixture was filtered, the catalyst rinsed with methanol (10 mL) and the organic solvent removed *in vacuo*. The residues were partitioned between dichloromethane and water, the organic layer separated, the solvent removed *in vacuo* and the residues subjected to column chromatography on silica. Elution with 0–100% ethyl acetate in petroleum ether (b.p. 40–60 °C) afforded 4-(4-(2-(1*H*-indol-3-yl-5-*d*)ethyl)pyrimidin-2-yl)morpholine **56** (78 mg, 84%, ∼95% *d* incorporation) as a colorless solid. ^1^H NMR: δ 10.75 (br s, 1H), 8.24 (d, *J* = 5 Hz, 1H), 7.53 (s, 1H), 7.33 (d, *J* = 8 Hz, 1H), 7.11 (d, *J* = 2 Hz, 1H), 7.06 (d, *J* = 8 Hz, 1H), 6.61 (d, *J* = 5 Hz, 1H), 3.70 (m, 4H), 3.66 (m, 4H), 3.08 (m, 2H), 2.93 (m, 2H). ^13^C NMR: δ 170.9 (C), 161.8 (C), 158.0 (CH), 136.7 (C), 127.5 (C), 122.8 (CH), 121.3 (CH), 118.6 (CH), 114.2 (C), 111.8 (CH), 110.0 (CH), 66.5 (CH_2_), 44.3 (CH_2_), 38.2 (CH_2_), 23.9 (CH_2_) [one signal missing]. LC-MS: [M + H]^+^ 310 (1.96, 100). HRMS: [M + H]^+^ 310.1774. Calcd for C_18_H_20_DN_4_O^+^: 310.1772. Δ = +0.6.

*4-(4-(2-(1-Methyl-1H-indol-*3-yl*)ethyl)pyrimidin-*2-yl*)morpholine (****57****)*. Prepared as per the method for **14** from 3-(2-(2-chloropyrimidin-4-yl)ethyl)-1-methyl-1*H*-indole **50** and morpholine. Yield 84%. Off-white solid. ^1^H NMR: δ 8.24 (d, *J* = 5 Hz, 1H), 7.54 (d, *J* = 8 Hz, 1H), 7.37 (d, *J* = 8 Hz, 1H), 7.13 (t, *J* = 8 Hz, 1H), 7.11 (s, 1H), 7.01 (t, *J* = 8 Hz, 1H), 6.61 (d, *J* = 5 Hz, 1H), 3.72 (s, 3H), 3.70 (m, 4H), 3.65 (m, 4H), 3.07 (t, *J* = 7 Hz, 2H), 2.92 (t, *J* = 7 Hz, 2H). ^13^C NMR: δ 170.8 (C), 161.8 (C), 158.0 (CH), 137.1 (C), 127.9 (C), 127.3 (CH), 121.5 (CH), 119.0 (CH), 118.7 (CH), 113.6 (C), 110.0 (CH), 110.0 (CH), 66.5 (CH_2_), 44.4 (CH_2_), 38.3 (CH_2_), 32.7 (CH_3_), 23.8 (CH_2_). LC-MS: [M + H]^+^ 323 (2.18, 96). HRMS: [M + H]^+^ 323.1864. Calcd for C_19_H_23_N_4_O^+^: 323.1866. Δ = −0.7.

*3-(4-(2-(1H-Indol-*3-yl*)ethyl)pyrimidin-*2-yl*)-8-oxa-3-azabicyclo[3.2.1]octane (****58****)*. Prepared as per the method for **31** from 3-(2-(2-chloropyrimidin-4-yl)ethyl)-1*H*-indole **11** and 8-oxa-3-azabicyclo[3.2.1]octane hydrochloride. Yield 60%. Off-white solid. ^1^H NMR: δ 10.75 (br s, 1H), 8.20 (d, *J* = 5 Hz, 1H), 7.52 (d, *J* = 8 Hz, 1H), 7.32 (d, *J* = 8 Hz, 1H), 7.11 (d, *J* = 2 Hz, 1H), 7.06 (t, *J* = 8 Hz, 1H), 6.96 (t, *J* = 8 Hz, 1H), 6.58 (d, *J* = 5 Hz, 1H), 4.40 (br m, 2H), 4.22 (d, *J* = 13 Hz, 2H), 3.07 (t, *J* = 7 Hz, 2H), 3.03 (dd, *J* = 13, 2.5 Hz, 2H), 2.92 (t, *J* = 7 Hz, 2H), 1.81 (m, 2H), 1.63 (m, 2H). ^13^C NMR: δ 170.7 (C), 162.6 (C), 157.8 (CH), 136.7 (C), 127.6 (C), 122.8 (CH), 121.3 (CH), 118.7 (CH), 118.6 (CH), 114.2 (C), 111.8 (CH), 109.9 (CH), 73.4 (CH), 49.9 (CH_2_), 38.2 (CH_2_), 28.0 (CH_2_), 23.9 (CH_2_). LC-MS: [M + H]^+^ 335 (2.02, 95). HRMS: [M + H]^+^ 335.1873. Calcd for C_20_H_23_N_4_O^+^: 335.1867. Δ = +1.8.

*3-(4-(2-(5-Fluoro-1H-indol-*3-yl*)ethyl)pyrimidin-*2-yl*)-8-oxa-3-azabicyclo[3.2.1]octane (****59****)*. Prepared as per the method for **31** from 3-(2-(2-chloropyrimidin-4-yl)ethyl)-5-fluoro-1*H*-indole **46** and 8-oxa-3-azabicyclo[3.2.1]octane hydrochloride. Yield 92%. Yellow oil. ^1^H NMR: δ 10.86 (br s, 1H), 8.19 (d, *J* = 5 Hz, 1H), 7.30 (dd, *J* = 9, 4.5 Hz, 1H), 7.26 (dd, *J* = 10, 2.5 Hz, 1H), 7.20 (d, *J* = 1.5 Hz, 1H), 6.89 (td, *J* = 9, 2.5 Hz, 1H), 6.58 (d, *J* = 5 Hz, 1H), 4.40 (br m, 2H), 4.22 (d, *J* = 13 Hz, 2H), 3.04 (t, *J* = 7 Hz, 2H), 3.02 (dd, *J* = 13, 2 Hz, 2H), 2.90 (t, *J* = 7 Hz, 2H), 1.80 (m, 2H), 1.63 (m, 2H). ^13^C NMR: δ 170.6 (C), 162.6 (C), 157.8 (CH), 157.0 (d, *J* = 232 Hz, CF), 133.3 (C), 127.8 (d, *J* = 9.5 Hz, CCHCF), 125.0 (CH), 114.5 (d, *J* = 5 Hz, CCHCHCF), 112.6 (d, *J* = 9.5 Hz, CHCHCF), 109.9 (CH), 109.4 (d, *J* = 26 Hz, CHCF), 103.4 (d, *J* = 23 Hz, CHCF), 73.4 (CH), 49.9 (CH_2_), 38.1 (CH_2_), 28.0 (CH_2_), 23.8 (CH_2_). ^19^F NMR: δ −125.7. LC-MS: [M + H]^+^ 353 (2.08, 100). HRMS: [M + H]^+^ 353.1775. Calcd for C_20_H_22_FN_4_O^+^: 353.1772. Δ = +0.8.

*4-(4-(2-(1H-Indol-*3-yl*)ethyl)pyrimidin-*2-yl*)-1,4-oxazepane (****60****)*. Prepared as per the method for **31** from 3-(2-(2-chloropyrimidin-4-yl)ethyl)-1*H*-indole **11** and 1,4-oxazepane. Yield 88%. Colorless solid. ^1^H NMR: δ 10.75 (br s, 1H), 8.19 (d, *J* = 5 Hz, 1H), 7.51 (d, *J* = 8 Hz, 1H), 7.32 (d, *J* = 8 Hz, 1H), 7.10 (d, *J* = 2 Hz, 1H), 7.06 (t, *J* = 8 Hz, 1H), 6.96 (t, *J* = 8 Hz, 1H), 6.53 (d, *J* = 5 Hz, 1H), 3.85 (m, 4H), 3.70 (m, 2H), 3.59 (m, 2H), 3.07 (t, *J* = 7.5 Hz, 2H), 2.92 (t, *J* = 7.5 Hz, 2H, 2H), 1.85 (m, 2H). ^13^C NMR: δ 170.8 (C), 161.5 (C), 158.0 (CH), 136.7 (C), 127.6 (C), 122.7 (CH), 121.3 (CH), 118.7 (CH), 118.6 (CH), 114.2 (C), 111.8 (CH), 109.1 (CH), 69.6 (CH_2_), 69.5 (CH_2_), 49.3 (CH_2_), 45.6 (CH_2_), 38.3 (CH_2_), 29.6 (CH_2_), 24.0 (CH_2_). LC-MS: [M + H]^+^ 323 (1.88, 100). HRMS: [M + H]^+^ 323.1869. Calcd for C_19_H_23_N_4_O^+^: 323.1866. Δ = +0.9.

*4-(4-(2-(1H-Indol-*3-yl*)ethyl)pyrimidin-*2-yl*)-3,4-dihydro-2H-benzo[b][1,4]oxazine (****61****)*. A mixture of 3-(2-(2-chloropyrimidin-4-yl)ethyl)-1*H*-indole **11** (77 mg, 0.3 mmol) and 3,4-dihydro-2*H*-benzo[*b*][1,4]oxazine (0.1 mL) in ethanol (0.5 mL) was stirred and held at 120 °C in a sealed tube for 3 h and allowed to cool to room temperature. The solvent was removed *in vacuo* and the residues partitioned between dichloromethane and water. The organic layer was separated, the solvent removed *in vacuo* and the residues subjected to column chromatography on silica. Elution with 0–100% ethyl acetate in petroleum ether (b.p. 40–60 °C) afforded 4-(4-(2-(1*H*-Indol-3-yl)ethyl)pyrimidin-2-yl)-3,4-dihydro-2*H*-benzo[*b*][1,4]oxazine **61** (100 mg, 93%) as a colourless solid. ^1^H NMR: δ 10.76 (br s, 1H), 8.38 (d, *J* = 5 Hz, 1H), 7.97 (dd, *J* = 8, 1.5 Hz, 1H), 7.53 (dd, *J* = 8, 1 Hz, 1H), 7.33 (dd, *J* = 8, 1 Hz, 1H), 7.10 (d, *J* = 2 Hz, 1H), 7.07 (td, *J* = 8, 1 Hz, 1H), 6.98 (td, *J* = 8, 1 Hz, 1H), 6.94 (td, *J* = 8, 1.5 Hz, 1H), 6.87 (dd, *J* = 8, 1.5 Hz, 1H), 6.85 (d, *J* = 5 Hz, 1H), 6.81 (td, *J* = 8, 1.5 Hz, 1H), 4.24 (m, 2H), 4.19 (m, 2H), 3.13 (t, *J* = 8 Hz, 2H), 3.02 (t, *J* = 8 Hz, 2H). ^13^C NMR: δ 171.2 (C), 159.6 (C), 158.0 (CH), 146.4 (C), 136.8 (C), 127.9 (C), 127.5 (C), 124.3 (CH), 123.9 (CH), 122.8 (CH), 121.4 (CH), 119.8 (CH), 118.7 (CH), 118.6 (CH), 117.0 (CH), 114.0 (C), 112.7 (CH), 111.8 (CH), 65.9 (CH_2_), 42.4 (CH_2_), 38.1 (CH_2_), 24.0 (CH_2_). LC-MS: [M + H]^+^ 357 (2.34, 100). HRMS: [M + H]^+^ 357.1528. Calcd for C_22_H_21_N_4_O^+^: 357.1529. Δ = −0.3.

*4-(4-(2-(5-Fluoro-1H-indol-*3-yl*)ethyl)pyrimidin-*2-yl*)-3,4-dihydro-2H-benzo[b][1,4]oxazine (****62****)*. Prepared as per the method for **61** from 3-(2-(2-chloropyrimidin-4-yl)ethyl)-5-fluoro-1*H*-indole **62** and 3,4-dihydro-2*H*-benzo[*b*][1,4]oxazine. Yield 58%. Yellow oil. ^1^H NMR: δ 10.87 (br s, 1H), 8.38 (d, *J* = 5 Hz, 1H), 7.95 (dd, *J* = 8.5, 1.5 Hz, 1H), 7.32 (dd, *J* = 9, 4.5 Hz, 1H), 7.26 (dd, *J* = 10, 2.5 Hz, 1H), 7.18 (d, *J* = 2 Hz, 1H), 6.94 (td, *J* = 8.5, 1.5 Hz, 1H), 6.90 (td, *J* = 9, 2.5 Hz, 1H), 6.87 (dd, *J* = 8.5, 1.5 Hz, 1H), 6.85 (d, *J* = 5 Hz, 1H), 6.81 (td, *J* = 8.5, 1.5 Hz, 1H), 4.24 (m, 2H), 4.19 (m, 2H), 3.09 (t, *J* = 7.5 Hz, 2H), 3.00 (t, *J* = 7.5 Hz, 2H). ^13^C NMR: δ 171.1 (C), 159.5 (C), 158.0 (CH), 157.1 (d, *J* = 231 Hz, CF), 146.3 (C), 133.4 (C), 127.9 (C), 127.8 (d, *J* = 9.5 Hz, CCHCF), 125.1 (CH), 124.3 (CH), 123.9 (CH), 119.7 (CH), 117.0 (CH), 114.3 (d, *J* = 5 Hz, CCHCHCF), 112.7 (d, *J* = 9.5 Hz, CHCHCF), 112.6 (CH), 109.4 (d, *J* = 25.5 Hz, CHCF), 103.4 (d, *J* = 22.5 Hz, CHCF), 65.9 (CH_2_), 42.4 (CH_2_), 38.0 (CH_2_), 23.8 (CH_2_). ^19^F NMR: δ −125.6. LC-MS: [M + H]^+^ 375 (2.39, 100). HRMS: [M + H]^+^ 375.1616. Calcd for C_22_H_20_FN_4_O^+^: 375.1616. Δ = 0.

*4-(4-(2-(1H-Indol-*3-yl*)ethyl)pyrimidin-*2-yl*)-6-bromo-3,4-dihydro-2H-benzo[b][1,4]oxazine (****63****)*. A mixture of 3-(2-(2-chloropyrimidin-4-yl)ethyl)-1*H*-indole **11** (77 mg, 0.3 mmol) and 6-bromo-3,4-dihydro-2*H*-benzo[*b*][1,4]oxazine (214 mg, 1.0 mmol) in ethanol (0.5 mL) was stirred and held at 160 °C with microwave irradiation for 1 h and allowed to cool to room temperature. The solvent was removed *in vacuo* and the residues partitioned between dichloromethane and water. The organic layer was separated, the solvent removed *in vacuo* and the residues subjected to column chromatography on silica. Elution with 0–60% ethyl acetate in petroleum ether (b.p. 40–60 °C) afforded 4-(4-(2-(1*H*-indol-3-yl)ethyl)pyrimidin-2-yl)-6-bromo-3,4-dihydro-2*H*-benzo[*b*][1,4]oxazine **63** (120 mg, 92%) as a pale orange solid. ^1^H NMR: δ 10.77 (br s, 1H), 8.45 (d, *J* = 2 Hz, 1H), 8.44 (d, *J* = 5 Hz, 1H), 7.57 (d, *J* = 8 Hz, 1H), 7.33 (d, *J* = 8 Hz, 1H), 7.13 (d, *J* = 2 Hz, 1H), 7.11 (dd, *J* = 8, 2 Hz, 1H), 7.06 (t, *J* = 8 Hz, 1H), 6.97 (t, *J* = 8 Hz, 1H), 6.91 (d, *J* = 5 Hz, 1H), 6.87 (d, *J* = 8 Hz, 1H), 4.27 (m, 2H), 4.21 (m, 2H), 3.16 (t, *J* = 8 Hz, 2H), 3.05 (t, *J* = 8 Hz, 2H). ^13^C NMR: δ 171.2 (C), 159.2 (C), 158.2 (CH), 145.6 (C), 136.7 (C), 129.4 (C), 127.5 (C), 126.1 (CH), 126.0 (CH), 122.8 (CH), 121.4 (CH), 118.9 (CH), 118.8 (CH), 118.7 (CH), 114.0 (C), 113.3 (CH), 111.8 (CH), 111.0 (C), 65.8 (CH_2_), 42.2 (CH_2_), 38.1 (CH_2_), 23.9 (CH_2_). LC-MS: [M + H]^+^ 435, 437 (2.50, 96). HRMS: [M + H]^+^ 435.0802. Calcd for C_22_H_20_^79^BrN_4_O^+^:435.0815. Δ = −3.0.

*1-(3-(2-(2-Morpholinopyrimidin-*4-yl*)ethyl)-1H-indol-*1-yl*)ethan-1-one (****64****)*. A solution of 4-(4-(2-(1*H*-indol-3-yl)ethyl)pyrimidin-2-yl)morpholine **14** (61.6 mg, 0.2 mmol) in acetic anhydride (2 mL) was stirred and held at reflux for 32 h and allowed to cool to room temperature. The solvent was removed *in vacuo* and the residues partitioned between dichloromethane and an aqueous solution of sodium carbonate. The organic layer was separated, the solvent removed *in vacuo* and the residues subjected to column chromatography on silica. Elution with 0–100% ethyl acetate in petroleum ether (b.p. 40–60 °C) followed by rinsing of the solids with 25% diethyl ether in petroleum ether (b.p. 40–60 °C) afforded 1-(3-(2-(2-morpholinopyrimidin-4-yl)ethyl)-1*H*-indol-1-yl)ethan-1-one **64** (40 mg, 57%) as a pale yellow solid. ^1^H NMR: δ 8.31 (dt, *J* = 8, 1 Hz, 1H), 8.27 (d, *J* = 5 Hz, 1H), 7.68 (s, 1H), 7.63 (dt, *J* = 8, 1 Hz, 1H), 7.33 (td, *J* = 8, 1 Hz, 1H), 7.28 (td, *J* = 8, 1 Hz, 1H), 6.66 (d, *J* = 5 Hz, 1H), 3.70 (m, 4H), 3.65 (m, 4H), 3.07 (m, 2H), 2.99 (m, 2H), 2.61 (s, 3H). ^13^C NMR: δ 170.3 (C), 169.6 (C=O), 161.8 (C), 158.2 (CH), 135.6 (C), 130.8 (C), 125.2 (CH), 124.1 (CH), 123.6 (CH), 121.4 (C), 119.5 (CH), 116.4 (CH), 109.9 (CH), 66.5 (CH_2_), 44.3 (CH_2_), 36.9 (CH_2_), 24.3 (CH_3_), 23.4 (CH_2_). LC-MS: [M + H]^+^ 351 (2.07, 100). HRMS: [M + H]^+^ 351.1816. Calcd for C_20_H_23_N_4_O_2_^+^: 351.1816. Δ = 0.

*4-(4-(2-(1-(Methylsulphonyl)-1H-indol-*3-yl*)ethyl)pyrimidin-*2-yl*)morpholine (****65****)*. Methanesulphonyl chloride (84 μL, 0.6 mmol) in benzene (0.6 mL) was added dropwise to a rapidly stirred mixture of 4-(4-(2-(1*H*-indol-3-yl)ethyl)pyrimidin-2-yl)morpholine **14** (62 mg, 0.2 mmol) and tetra-*n*-butylammonium hydrogen sulphate (20 mg, 0.06 mmol) in benzene (0.6 mL) and 50% w/w aqueous sodium hydroxide solution (0.6 mL) [21] and the mixture was stirred and held at room temperature for 3 h. The mixture was diluted with dichloromethane and water, the organic layer was separated, the solvent removed *in vacuo* and the residues subjected to column chromatography on silica. Elution with 0–100% ethyl acetate in petroleum ether (b.p. 40–60 °C) afforded 4-(4-(2-(1-(methylsulphonyl)-1*H*-indol-3-yl)ethyl)pyrimidin-2-yl)morpholine **65** (52 mg, 67%) as a colorless solid. ^1^H NMR: δ 8.25 (d, *J* = 5 Hz, 1H), 7.81 (d, *J* = 8 Hz, 1H), 7.68 (d, *J* = 8 Hz, 1H), 7.38 (t, *J* = 8 Hz, 1H), 7.37 (s, 1H), 7.32 (t, *J* = 8 Hz, 1H), 6.62 (d, *J* = 5 Hz, 1H), 3.68 (m, 4H), 3.63 (m, 4H), 3.30 (s, 3H), 3.09 (t, *J* = 7.5 Hz, 2H), 2.98 (t, *J* = 7.5 Hz, 2H). ^13^C NMR: δ 170.3 (C), 161.8 (C), 158.2 (CH), 135.2 (C), 130.8 (C), 125.1 (CH), 123.7 (CH), 123.4 (CH), 121.5 (C), 120.2 (CH), 113.4 (CH), 110.0 (CH), 66.5 (CH_2_), 44.3 (CH_2_), 41.0 (CH_3_), 36.7 (CH_2_), 23.3 (CH_2_). LC-MS: [M + H]^+^ 387 (2.06, 100). HRMS: [M + H]^+^ 387.1481. Calcd for C_19_H_23_N_4_O_3_^32^S^+^: 387.1486. Δ = −1.3.

*tert-Butyl 4-morpholino-2-vinyl-5,7-dihydro-6H-pyrrolo[3,4-d]pyrimidine-6-carboxylate (****70****)*. A mixture of *tert*-butyl 2-chloro-4-morpholino-5,7-dihydro-6*H*-pyrrolo[3,4-*d*]pyrimidine-6-carboxylate **66** [23] (170 mg, 0.5 mmol), 4,4,5,5-tetramethyl-2-vinyl-1,3,2-dioxaborolane (123 mg, 0.8 mmol), tetrakis(triphenylphosphine)palladium(0) (57.8 mg, 0.05 mmol) and anhydrous sodium carbonate (318 mg, 3.0 mmol) in 1,4-dioxane (4 mL) and water (1 mL) was degassed with nitrogen for 5 min and stirred and held at 100 °C in a sealed tube for 3 h and allowed to cool to room temperature. Ethyl acetate and water were added, the organic layer was separated, the solvent removed *in vacuo* and the residues subjected to column chromatography on silica. Elution with 0–50% ethyl acetate in petroleum ether (b.p. 40–60 °C) afforded *tert*-butyl 4-morpholino-2-vinyl-5,7-dihydro-6*H*-pyrrolo[3,4-*d*]pyrimidine-6-carboxylate **70** (145 mg, 87%) as a colorless solid. ^1^H NMR: δ 6.60 (dd, *J* = 17, 10 Hz, 1H), 6.41 (dd, *J* = 17, 2 Hz, 1H), 5.62 (dd, *J* = 10, 2 Hz, 1H), 4.73 (br s, 2H), 4.39 (br s, 1H), 4.37 (br s, 1H), 3.67 (m, 8H), 1.46 (s, 9H). ^13^C NMR: δ 166.6 (C), 166.1 (C), 162.5 (C), 158.1 (C=O), 137.3 (CH), 123.0 (CH_2_), 108.9 (C), 79.8 (C), 66.5 (CH_2_), 45.4 (CH_2_), 28.6 (CH_3_) [two signals missing]. LC-MS: [M + H]^+^ 333 (1.73, 98). HRMS: [M + Na]^+^ 355.1741. Calcd for C_17_H_24_N_4_O_3_Na^+^: 355.1740. Δ = +0.2.

*tert-Butyl 4-(8-oxa-3-azabicyclo[3.2.1]octan-*3-yl*)-2-vinyl-5,7-dihydro-6H-pyrrolo[3,4-d]pyrimidine-6-carboxylate (****71****)*. Prepared as per the method for **70** from *tert*-butyl 4-(8-oxa-3-azabicyclo[3.2.1]octan-3-yl)-2-chloro-5,7-dihydro-6*H*-pyrrolo[3,4-*d*]pyrimidine-6-carboxylate **67** [24] and 4,4,5,5-tetramethyl-2-vinyl-1,3,2-dioxaborolane. Yield 59%. Colorless solid. ^1^H NMR: δ 6.60 (dd, *J* = 17.5, 10.5 Hz, 1H), 6.40 (dd, *J* = 17.5, 2 Hz, 1H), 5.61 (dd, *J* = 10.5, 2 Hz, 1H), 4.74 (br s, 2H), 4.42 (br s, 2H), 4.37 (d, *J* = 12.5 Hz, 2H), 3.97 (m, 2H), 3.20 (dm, *J* = 12.5 Hz, 2H), 1.81 (m, 2H), 1.75 (m, 2H), 1.46 (s, 9H). ^13^C NMR: δ 166.3 (C), 162.5 (C), 159.3 (C), 154.0 (C=O), 137.4 (CH), 123.0 (CH_2_), 108.5 (C), 79.7 (C), 73.4 (CH), 50.7 (CH_2_), 28.6 (CH_3_), 27.8 (CH_2_) [two signals missing]. LC-MS: [M + H]^+^ 359 (1.83, 100). HRMS: [M + H]^+^ 359.2077. Calcd for C_19_H_27_N_4_O_3_^+^: 359.2078. Δ = −0.3.

*tert-Butyl 4-morpholino-2-vinyl-5,8-dihydropyrido[3,4-d]pyrimidine-7(6H)-carboxylate (****72****)*. Prepared as per the method for **70** from *tert*-butyl 2-chloro-4-morpholino-5,8-dihydropyrido[3,4-*d*]pyrimidine-7(6*H*)-carboxylate **68** [24] and 4,4,5,5-tetramethyl-2-vinyl-1,3,2-dioxaborolane. Yield 84%. Off-white solid. ^1^H NMR: δ 6.61 (dd, *J* = 17.5, 10.5 Hz, 1H), 6.41 (dd, *J* = 17.5, 2.5 Hz, 1H), 5.62 (dd, *J* = 10.5, 2.5 Hz, 1H), 4.40 (br s, 2H), 3.70 (m, 4H), 3.50 (m, 2H), 3.40 (m, 4H), 2.65 (t, *J* = 5 Hz, 2H), 1.45 (s, 9H). ^13^C NMR: δ 164.5 (C), 164.4 (C), 160.2 (C), 154.1 (C=O), 137.3 (CH), 122.8 (CH_2_), 114.4 (C), 79.8 (C), 66.5 (CH_2_), 48.3 (CH_2_), 28.5 (CH_3_) [three signals missing]. LC-MS: [M + H]^+^ 347 (1.61, 100). HRMS: [M + H]^+^ 347.2069. Calcd for C_18_H_27_N_4_O_3_^+^: 347.2078. Δ = −2.6.

*tert-Butyl 4-(8-oxa-3-azabicyclo[3.2.1]octan-*3-yl*)-2-vinyl-5,8-dihydropyrido[3,4-d]pyrimidine-7(6H)-carboxylate (****73****)*. Prepared as per the method for **70** from *tert*-butyl 4-(8-oxa-3-azabicyclo[3.2.1]octan-3-yl)-2-chloro-5,8-dihydropyrido[3,4-*d*]pyrimidine-7(6*H*)-carboxylate **69** [24] and 4,4,5,5-tetramethyl-2-vinyl-1,3,2-dioxaborolane. Yield 87%. Off-white solid. ^1^H NMR: δ 6.59 (dd, *J* = 17.5, 10 Hz, 1H), 6.39 (dd, *J* = 17.5, 2 Hz, 1H), 5.60 (dd, *J* = 10, 2 Hz, 1H), 4.37 (br d, *J* = 11.5 Hz, 4H), 3.75 (br d, *J* = 13 Hz, 2H), 3.48 (m, 2H), 3.13 (dm, *J* = 13 Hz, 2H), 2.65 (t, *J* = 5.5 Hz, 2H), 1.82 (m, 4H), 1.44 (s, 9H). ^13^C NMR: δ 164.8 (C), 164.7 (C), 159.9 (C), 154.1 (C=O), 137.3 (CH), 122.6 (CH_2_), 113.4 (C), 79.8 (C), 73.9 (CH), 52.7 (CH_2_), 28.5 (CH_3_), 27.7 (CH_2_) [three signals missing]. LC-MS: [M + H]^+^ 373 (1.64, 97). HRMS: [M + H]^+^ 373.2231. Calcd for C_20_H_29_N_4_O_3_^+^: 373.2234. Δ = −0.9.

*tert-Butyl (E)-3-(2-(4,4,5,5-tetramethyl-1,3,2-dioxaborolan-*2-yl*)vinyl)-1H-indole-1-carboxylate (****74****)* [25]. Prepared as per the method for (*E*)-4,4,5,5-tetramethyl-2-styryl-1,3,2-dioxaborolane [27] from *tert*-butyl 3-ethynyl-1*H*-indole-1-carboxylate [26] and 4,4,5,5-tetramethyl-1,3,2-dioxaborolane. Yield 75%. Pale yellow oil. ^1^H NMR: δ 8.11 (d, *J* = 8.5 Hz, 1H), 8.06 (s, 1H), 7.97 (d, *J* = 8.5 Hz, 1H), 7.48 (d, *J* = 19 Hz, 1H), 7.39 (t, *J* = 8.5 Hz, 1H), 7.32 (t, *J* = 8.5 Hz, 1H), 6.17 (d, *J* = 19 Hz, 1H), 1.64 (s, 9H), 1.27 (s, 12H). ^13^C NMR: δ 149.3 (C=O), 141.5 (CH), 135.9 (C), 128.2 (C), 127.8 (CH), 125.4 (CH), 123.9 (CH), 120.8 (CH), 119.4 (C), 116.9 (br CHB), 115.4 (CH), 84.8 (C), 83.4 (C), 28.1 (CH_3_), 25.1 (CH_3_). ^11^B NMR: δ 29.1. LC-MS: [M + H]^+^ 370 (2.76, 100). HRMS: [M + H–C_4_H_8_]^+^ 314.1552. Calcd for C_17_H_21_BNO_4_^+^: 314.1564. Δ = −3.9.

*tert-Butyl (E)-2-(2-(1-(tert-butoxycarbonyl)-1H-indol-*3-yl*)vinyl)-4-morpholino-5,7-dihydro-6H-pyrrolo[3,4-d]pyrimidine-6-carboxylate (****75****)*. Prepared as per the method for **70** from *tert*-butyl 2-chloro-4-morpholino-5,7-dihydro-6*H*-pyrrolo[3,4-*d*]pyrimidine-6-carboxylate **66** and *tert*-butyl (*E*)-3-(2-(4,4,5,5-tetramethyl-1,3,2-dioxaborolan-2-yl)vinyl)-1*H*-indole-1-carboxylate **74**. Yield 73%. Colorless solid. ^1^H NMR: δ 8.17 (s, 1H), 8.15 (d, *J* = 7 Hz, 1H), 8.01 (d, *J* = 7 Hz, 1H), 8.00 (d, *J* = 16 Hz, 1H), 7.42 (t, *J* = 7 Hz, 1H), 7.37 (t, *J* = 7 Hz, 1H), 7.12 (d, *J* = 16 Hz, 1H), 4.75 (br s, 2H), 4.42 (br s, 1H), 4.40 (br s, 1H), 3.72 (s, 8H), 1.66 (s, 9H), 1.48 (s, 9H). ^13^C NMR: δ 166.6 (C), 166.1 (C), 163.3 (C), 158.2 (C=O), 149.3 (C=O), 136.0 (C), 128.6 (CH), 128.3 (C), 128.0 (CH), 127.8 (CH), 125.5 (CH), 124.0 (CH), 120.8 (CH), 117.9 (C), 117.8 (C), 115.5 (CH), 84.8 (C), 74.0 (C), 66.6 (CH_2_), 45.6 (CH_2_), 28.2 (CH_3_), 25.4 (CH_3_) [two signals missing]. LC-MS: [M + H]^+^ 548 (2.54, 100). HRMS: [M + H]^+^ 548.2870. Calcd for C_30_H_38_N_5_O_5_^+^: 548.2873. Δ = −0.6.

*tert-Butyl (E)-4-(8-oxa-3-azabicyclo[3.2.1]octan-*3-yl*)-2-(2-(1-(tert-butoxycarbonyl)-1H-indol-*3-yl*)vinyl)-5,7-dihydro-6H-pyrrolo[3,4-d]pyrimidine-6-carboxylate (****76****)*. Prepared as per the method for **70** from *tert*-butyl 4-(8-oxa-3-azabicyclo[3.2.1]octan-3-yl)-2-chloro-5,7-dihydro-6*H*-pyrrolo[3,4-*d*]pyrimidine-6-carboxylate **67** and *tert*-butyl (*E*)-3-(2-(4,4,5,5-tetramethyl-1,3,2-dioxaborolan-2-yl)vinyl)-1*H*-indole-1-carboxylate **74**. Yield 35%. Pale yellow solid. ^1^H NMR: δ 8.17 (s, 1H), 8.15 (d, *J* = 7 Hz, 1H), 8.00 (d, *J* = 7 Hz, 1H), 7.99 (d, *J* = 17 Hz, 1H), 7.43 (t, *J* = 7 Hz, 1H), 7.38 (t, *J* = 7 Hz, 1H), 7.12 (d, *J* = 17 Hz, 1H), 4.76 (br s, 2H), 4.45 (br s, 2H), 4.40 (d, *J* = 10 Hz, 2H), 4.05 (m, 2H), 3.24 (dm, *J* = 12.5 Hz, 2H), 1.82 (m, 4H), 1.66 (s, 9H), 1.48 (s, 9H). ^13^C NMR: δ 167.5 (C), 166.2 (C), 163.5 (C), 159.4 (C=O), 149.3 (C=O), 136.1 (C), 128.7 (CH), 128.6 (CH), 128.0 (C), 127.8 (CH), 125.5 (CH), 124.0 (CH), 120.8 (CH), 117.9 (C), 115.5 (CH), 107.8 (C), 84.8 (C), 79.7 (C), 73.5 (CH), 50.8 (CH_2_), 28.6 (CH_3_), 28.2 (CH_3_) 27.9 (CH_2_) [two signals missing]. LC-MS: [M + H]^+^ 574 (2.61, 100). HRMS: [M + H]^+^ 574.3029. Calcd for C_32_H_40_N_5_O_5_^+^: 574.3029. Δ = 0.

*tert-Butyl (E)-2-(2-(1-(tert-butoxycarbonyl)-1H-indol-*3-yl*)vinyl)-4-morpholino-5,8-dihydropyrido[3,4-d]pyrimidine-7(6H)-carboxylate (****77****)*. Prepared as per the method for **70** from *tert*-butyl 2-chloro-4-morpholino-5,8-dihydropyrido[3,4-*d*]pyrimidine-7(6*H*)-carboxylate **68** and *tert*-butyl (*E*)-3-(2-(4,4,5,5-tetramethyl-1,3,2-dioxaborolan-2-yl)vinyl)-1*H*-indole-1-carboxylate **74**. Yield 35%. Tan solid. ^1^H NMR: δ 8.18 (s, 1H), 8.16 (d, *J* = 8 Hz, 1H), 8.00 (d, *J* = 8 Hz, 1H), 7.99 (d, *J* = 16 Hz, 1H), 7.43 (t, *J* = 8 Hz, 1H), 7.38 (t, *J* = 8 Hz, 1H), 7.14 (d, *J* = 16 Hz, 1H), 4.45 (br s, 2H), 3.75 (m, 4H), 3.53 (m, 2H), 3.47 (m, 4H), 2.67 (t, *J* = 5 Hz, 2H), 1.66 (s, 9H), 1.46 (s, 9H). ^13^C NMR: δ 164.6 (C), 161.4 (C), 161.0 (C), 154.2 (C=O), 149.3 (C=O), 136.0 (C), 128.4 (CH), 128.3 (C), 128.0 (CH), 127.8 (CH), 125.5 (CH), 124.1 (CH), 120.8 (CH), 117.8 (C), 115.5 (CH), 110.1 (C), 84.9 (C), 79.8 (C), 66.6 (CH_2_), 48.4 (CH_2_), 28.5 (CH_3_), 28.2 (CH_3_) [three signals missing]. LC-MS: [M + H]^+^ 562 (2.40, 100). HRMS: [M + H]^+^ 562.3024. Calcd for C_31_H_40_N_5_O_5_^+^: 562.3029. Δ = −0.9.

*tert-Butyl 2-(2-(1-(tert-butoxycarbonyl)-1H-indol-*3-yl*)ethyl)-4-morpholino-5,7-dihydro-6H-pyrrolo[3,4-d]pyrimidine-6-carboxylate (****78****)*. A mixture of *tert*-butyl (*E*)-2-(2-(1-(*tert*-butoxycarbonyl)-1*H*-indol-3-yl)vinyl)-4-morpholino-5,7-dihydro-6*H*-pyrrolo[3,4-*d*]pyrimidine-6-carboxylate **75** (66 mg, 0.12 mmol), 10% palladium on carbon (30 mg) in ethyl acetate (5 mL) was degassed with nitrogen for 2 min and then stirred and held at room temperature under an atmosphere of hydrogen gas for 4 h. The mixture was filtered, the catalyst rinsed with ethyl acetate (5 mL) and the solvent removed *in vacuo*. The residues were partitioned between dichloromethane and water, the organic layer separated, the solvent removed *in vacuo* and the residues subjected to column chromatography on silica. Elution with 0–80% ethyl acetate in petroleum ether (b.p. 40–60 °C) afforded *tert*-butyl 2-(2-(1-(*tert*-butoxycarbonyl)-1*H*-indol-3-yl)ethyl)-4-morpholino-5,7-dihydro-6*H*-pyrrolo[3,4-*d*]pyrimidine-6-carboxylate **78** (48 mg, 73%) as a colorless solid. ^1^H NMR: δ 8.03 (d, *J* = 8 Hz, 1H), 7.60 (d, *J* = 8 Hz, 1H), 7.43 (s, 1H), 7.32 (t, *J* = 8.5 Hz, 1H), 7.25 (t, *J* = 8.5 Hz, 1H), 4.70 (br s, 2H), 4.37 (br s, 1H), 4.34 (br s, 1H), 3.64 (m, 4H), 3.61 (m, 4H), 3.11 (m, 2H), 3.06 (m, 2H), 1.62 (s, 9H), 1.46 (s, 9H). ^13^C NMR: δ 168.5 (C), 166.5 (C), 166.1 (C), 158.2 (C=O), 149.6 (C=O), 135.2 (C), 130.7 (C), 124.8 (CH), 123.0 (CH), 122.9 (CH), 120.8 (C), 120.7 (C), 119.6 (CH), 115.1 (CH), 83.8 (C), 79.8 (C), 66.5 (CH_2_), 45.4 (CH_2_), 38.1 (CH_2_), 23.0 (CH_2_), 28.6 (CH_3_), 28.2 (CH_3_) [two signals missing]. LC-MS: [M + H]^+^ 550 (2.33, 100). HRMS: [M + H]^+^ 550.3026. Calcd for C_30_H_40_N_5_O_5_^+^: 550.3029. Δ = −0.6.

*tert-Butyl 4-(8-oxa-3-azabicyclo[3.2.1]octan-*3-yl*)-2-(2-(1-(tert-butoxycarbonyl)-1H-indol-*3-yl*)ethyl)-5,7-dihydro-6H-pyrrolo[3,4-d]pyrimidine-6-carboxylate (****79****)*. Prepared as per the method for **78** from *tert*-butyl (*E*)-4-(8-oxa-3-azabicyclo[3.2.1]octan-3-yl)-2-(2-(1-(*tert*-butoxycarbonyl)-1*H*-indol-3-yl)vinyl)-5,7-dihydro-6*H*-pyrrolo[3,4-*d*]pyrimidine-6-carboxylate **76**. Yield 52%. Off-white solid. ^1^H NMR: δ 8.02 (d, *J* = 8 Hz, 1H), 7.59 (d, *J* = 8 Hz, 1H), 7.43 (s, 1H), 7.32 (t, *J* = 8 Hz, 1H), 7.24 (t, *J* = 8 Hz, 1H), 4.70 (br s, 2H), 4.37 (br s, 2H), 4.33 (d, *J* = 12.5 Hz, 2H), 3.92 (m, 2H), 3.12 (m, 4H), 3.04 (m, 2H), 1.78 (m, 2H), 1.66 (m, 2H), 1.62 (s, 9H), 1.46 (s, 9H). ^13^C NMR: δ 168.3 (C), 168.1 (C), 166.1 (C), 159.3 (C=O), 149.6 (C=O), 135.2 (C), 130.7 (C), 124.8 (CH), 123.1 (CH), 122.9 (CH), 119.7 (CH), 115.1 (CH), 107.7 (C), 107.3 (C), 83.9 (C), 79.7 (C), 73.3 (CH), 50.7 (CH_2_), 38.1 (CH_2_), 28.6 (CH_3_), 28.2 (CH_3_), 27.7 (CH_2_), 23.0 (CH_2_) [two signals missing]. LC-MS: [M + H]^+^ 576 (2.38, 100). HRMS: [M + H]^+^ 576.3192. Calcd for C_32_H_42_N_5_O_5_^+^: 576.3186. Δ = +1.0.

*tert-Butyl 2-(2-(1-(tert-butoxycarbonyl)-1H-indol-*3-yl*)ethyl)-4-morpholino-5,8-dihydropyrido[3,4-d]pyrimidine-7(6H)-carboxylate****80****)*. Prepared as per the method for **78** from *tert*-butyl (*E*)-2-(2-(1-(*tert*-butoxycarbonyl)-1*H*-indol-3-yl)vinyl)-4-morpholino-5,8-dihydropyrido[3,4-*d*]pyrimidine-7(6*H*)-carboxylate **77**. Yield 43%. Colorless solid. ^1^H NMR: δ 8.02 (d, *J* = 8 Hz, 1H), 7.58 (d, *J* = 8 Hz, 1H), 7.44 (s, 1H), 7.31 (t, *J* = 8 Hz, 1H), 7.24 (t, *J* = 8 Hz, 1H), 4.37 (br s, 2H), 3.66 (m, 4H), 3.48 (m, 2H), 3.36 (m, 4H), 3.12 (m, 2H), 3.06 (m, 2H), 2.61 (t, *J* = 5 Hz, 2H), 1.62 (s, 9H), 1.44 (s, 9H). ^13^C NMR: δ 166.1 (C), 166.0 (C), 164.4 (C), 154.1 (C=O), 149.5 (C=O), 135.2 (C), 130.8 (C), 124.8 (CH), 123.0 (CH), 122.9 (CH), 120.8 (C), 119.7 (CH), 115.2 (CH), 113.1 (C), 83.9 (C), 79.8 (C), 66.6 (CH_2_), 48.3 (CH_2_), 38.1 (CH_2_), 28.5 (CH_3_), 28.2 (CH_3_), 23.1 (CH_2_) [three signals missing]. LC-MS: [M + H]^+^ 564 (2.15, 100). HRMS: [M + H]^+^ 564.3181. Calcd for C_31_H_42_N_5_O_5_^+^: 564.3186. Δ = −0.9.

*4-(2-(2-(1H-Indol-*3-yl*)ethyl)-6,7-dihydro-5H-pyrrolo[3,4-d]pyrimidin-*4-yl*)morpholine hydrochloride (****81****)*. Prepared as per the method for **37** from *tert*-butyl 2-(2-(1-(*tert*-butoxycarbonyl)-1*H*-indol-3-yl)ethyl)-4-morpholino-5,7-dihydro-6*H*-pyrrolo[3,4-*d*]pyrimidine-6-carboxylate **78**. Yield 78%. Pale tan solid. ^1^H NMR: δ 10.84 (br s, 1H), 10.57 (br s, 2H), 7.55 (d, *J* = 8 Hz, 1H), 7.34 (d, *J* = 8 Hz, 1H), 7.15 (d, *J* = 2 Hz, 1H), 7.07 (t, *J* = 8 Hz, 1H), 6.98 (t, *J* = 8 Hz, 1H), 4.70 (br s, 2H), 4.44 (br s, 2H), 3.74 (m, 4H), 3.68 (m, 4H), 3.18 (m, 4H). ^13^C NMR: δ 160.8 (C), 157.7 (C), 146.9 (C), 136.8 (C), 127.5 (C), 123.0 (CH), 121.4 (CH), 118.7 (CH), 118.7 (CH), 113.5 (C), 111.9 (CH), 106.8 (C), 66.4 (CH_2_), 49.4 (CH_2_), 48.4 (CH_2_), 46.2 (CH_2_), 37.1 (CH_2_), 23.2 (CH_2_). LC-MS: [M + H]^+^ 350 (1.24, 100). HRMS: [M + H]^+^ 350.1980. Calcd for C_20_H_24_N_5_O^+^: 350.1981. Δ = −0.3.

*3-(2-(2-(1H-Indol-*3-yl*)ethyl)-6,7-dihydro-5H-pyrrolo[3,4-d]pyrimidin-*4-yl*)-8-oxa-3-azabicyclo[3.2.1]octane hydrochloride (****82****)*. Prepared as per the method for **37** from *tert*-butyl 4-(8-oxa-3-azabicyclo[3.2.1]octan-3-yl)-2-(2-(1-(*tert*-butoxycarbonyl)-1*H*-indol-3-yl)ethyl)-5,7-dihydro-6*H*-pyrrolo[3,4-*d*]pyrimidine-6-carboxylate **79**. Yield 70%. Brown solid. ^1^H NMR: δ 10.83 (br s, 1H), 10.47 (br s, 2H), 7.54 (d, *J* = 7.5 Hz, 1H), 7.33 (d, *J* = 7.5 Hz, 1H), 7.14 (s, 1H), 7.07 (t, *J* = 7.5 Hz, 1H), 6.98 (t, *J* = 7.5 Hz, 1H), 4.70 (br s, 2H), 4.41 (br s, 2H), 4.03 (m, 4H), 3.29 (dm, *J* = 12.5 Hz, 2H), 3.16 (m, 4H), 1.80 (m, 2H), 1.62 (m, 2H). ^13^C NMR: δ 159.2 (C), 159.1 (C), 159.1 (C), 136.7 (C), 127.5 (C), 122.9 (CH), 121.4 (CH), 118.7 (CH), 118.7 (CH), 113.5 (C), 111.9 (CH), 106.8 (C), 73.4 (CH), 51.4 (CH_2_), 49.5 (CH_2_), 48.4 (CH_2_), 27.5 (CH_2_), 23.2 (CH_2_) [one signal missing]. LC-MS: [M + H]^+^ 376 (1.30, 98). HRMS: [M + H]^+^ 376.2137. Calcd for C_22_H_26_N_5_O^+^: 376.2137. Δ = 0.

*4-(2-(2-(1H-Indol-*3-yl*)ethyl)-5,6,7,8-tetrahydropyrido[3,4-d]pyrimidin-*4-yl*)morpholine hydrochloride (****83****)*. Prepared as per the method for **37** from *tert*-butyl 2-(2-(1-(*tert*-butoxycarbonyl)-1*H*-indol-3-yl)ethyl)-4-morpholino-5,8-dihydropyrido[3,4-*d*]pyrimidine-7(6*H*)-carboxylate **80**. Yield 75%. Tan solid. ^1^H NMR: δ 10.87 (br s, 1H), 10.07 (br s, 2H), 7.54 (d, *J* = 8 Hz, 1H), 7.34 (d, *J* = 8 Hz, 1H), 7.18 (s, 1H), 7.08 (t, *J* = 8 Hz, 1H), 6.99 (t, *J* = 8 Hz, 1H), 4.32 (br s, 2H), 3.74 (m, 4H), 3.69 (m, 4H), 3.27 (br s, 2H), 3.18 (s, 4H), 2.94 (br m, 2H). ^13^C NMR: δ 159.1 (C), 155.6 (C), 153.1 (C), 136.7 (C), 127.4 (C), 123.1 (CH), 121.5 (CH), 118.9 (C), 118.8 (CH), 118.7 (CH), 111.9 (CH), 109.9 (C), 66.5 (CH_2_), 48.4 (CH_2_), 40.2 (CH_2_), 23.1 (CH_2_), 23.0 (CH_2_) [two signals missing]. LC-MS: [M + H]^+^ 364 (1.18, 100). HRMS: [M + H]^+^ 364.2138. Calcd for C_21_H_26_N_5_O^+^: 364.2137. Δ = +0.2.

### 4.2 Expression and purification of CYP121A1

Untagged CYP121A1 was expressed and purified to homogeneity as reported previously [7,38]. A His_6_-tagged construct of CYP121A1 was also expressed and purified to homogeneity as reported previously [9] with minor modifications (as below). Purification was effected by immobilized metal ion affinity chromatography at 18 °C using an AKTA purifier (GE Healthcare, UK). The supernatant from ruptured cells was loaded *via* a superloop onto a HisTrap FF affinity column (GE Healthcare, UK) that had been equilibrated in 50 mM potassium phosphate, 50 mM KCl, 10% (v/v) glycerol, pH 8. After washing the column with twenty column volumes of equilibration buffer, CYP121A1 was eluted at 5 mL/min with equilibration buffer containing imidazole (using a stepped gradient of 0–200 mM imidazole in 40 mM steps) and collected as 4 mL fractions with monitoring for both protein and heme (respective absorbances at A_280_ and A_417_). The purity of fractions collected was assessed by SDS-PAGE gels. Pure fractions were combined and dialyzed overnight at 4 °C into fresh buffer (50 mM Tris-HCl, 1 mM EDTA, pH 7.2) using a Slide-A-Lyser™ dialysis cassette (ThermoScientific, UK), concentrated to around 500 μM in a bench top centrifuge (6000×*g*, 4 °C) using a Vivaspin (10,000 MWCO) ultrafiltration column (Sartorius, UK), apportioned into small aliquots, flash frozen with liquid nitrogen and stored at −80 °C until required. Final protein concentrations were determined using a NanoDrop 2000c spectrophotometer (Thermo Fisher Scientific, USA) by monitoring for heme absorbance at 417 nm (*ε* = 110,000 M^−1^ cm^−1^).

### 4.3 X-ray crystallography

Untagged CYP121A1 was expressed and purified to homogeneity as described above with an additional final polishing step utilizing a HiLoad Superdex 75 preparative grade 16/600 (GE Healthcare, UK). The column was equilibrated with 10 mM Tris, 100 mM KCl, pH 7.8 at 4 °C (adjusted with KOH/HCl) and CYP121A1 was eluted isocratically according to the manufacturer's instructions. Fractions with a calculated Reinheitszahl (Rz) ratio of ∼2 were pooled and concentrated using a Vivaspin 20 10,000 MWCO centrifugal concentrator (Sartorius, UK). A modified form of the Rz ratio can be derived as follows:

$$Rz=\;\frac{Absorbance\;maximum\;at\;∼416\;nm}{Absorbance\;maximum\;at\;280\;nm}$$

The crystallization conditions identified previously [39] were used as a starting point for crystallization trials. Optimization screens were set up with a Dragonfly Crystal (SPT Labtech, UK) using 3.5 M (NH_4_)_2_SO_4_ (Molecular Dimensions, UK) as the precipitant and 1 M MES pH 5–6.5 (Molecular Dimensions, UK) as the buffer system in three lens microplates (SwissSci, Switzerland). Precipitant concentration and pH ranged between 1.5 and 2.5 M and 5–6.5 respectively. Sitting drops were set up with a Mosquito Crystal (SPT Labtech, UK) using 20 mg/mL CYP121A1 in 1 μL drops with a 1:1 ratio of protein to mother liquor. Plates were incubated at 4 °C and crystallogenesis took between 3 and 7 days to occur. Mature crystals were approximately 1 mm in length with an arrowhead morphology. Crystals were either soaked with saturated compound solutions made up in fresh DMSO or solid compound was added directly to the drops. In both cases, 1 μL of mother liquor was removed from the reservoir and transferred to the drop containing crystals to prevent desiccation. During soaks with DMSO-solubilized compound, 1 μL of compound solution was introduced to the reservoir and thoroughly mixed before 0.5 μL was transferred to the drop, mixed and removed. The transfer process was performed a total of three times. Soaked crystals were periodically removed (up to ∼ 6 months), preserved in Parabar 10312 (Hampton Research, US) and flash-cooled in liquid nitrogen. Crystals were irradiated at the Diamond Light Source (Didcot, UK) using the i03, i04 or i04-1 beamlines using standard collection parameters. Data were automatically indexed, integrated, scaled and merged using the xia2 dials [40] and xia2 3dii pipelines [41,42]. Data analysis was performed with Xtriage [43], molecular replacement with Phaser-MR [44] (using PDB structure 1N40 [39] as the starting model for molecular replacement) and initial refinement with Phenix refine [45]. Ligand restraints were generated with AceDRG [46]. Feature-enhanced maps [47] and POLDER maps [48] were calculated to aid in model building, which was performed using WinCoot [49]. Figures for publication were produced using PyMOL 1.3 (Schrödinger Inc). For data collection and refinement statistics, see SI.

Accession codes and atomic coordinates for the X-ray structures of complexes of CYP121A1 with compounds **10** (7NQM), **14** (7NQN) and **21** (7NQO) have been deposited with the RCSB Protein Data Bank (www.rcsb.org) and will be released upon publication.

### 4.4 Antimycobacterial activity assays

*Mycobacterium tuberculosis* laboratory strain H37Rv was routinely cultured in Middlebrook 7H9 broth (BD, USA) supplemented with 10% (v/v) Albumin Dextrose Catalase (ADC) enrichment (BD, USA), 0.05% (v/v) tyloxapol and 0.02% (v/v) glycerol (c7H9). Liquid cultures were grown at 37 °C in 50 mL centrifugation tubes with rotation at 40 rpm until mid-exponential phase (OD_600_ ∼1). For drug susceptibility testing, two additional broth-based media were used: Middlebrook 7H9 broth with low BSA supplemented with 10% (v/v) of ADN enrichment (0.05% (w/v) Albumin, 2% (w/v) Dextrose, and 0.85% (w/v) NaCl), 0.05% (v/v) tyloxapol and 0.02% (v/v) glycerol (7H9-Low BSA) and Mycobacterial Minimal Medium with cholesterol consisting of 0.5 g/L l-asparagine, 1 g/L KH_2_PO_4_, 2.5 g/L Na_2_HPO_4_, 50 mg/L ferric ammonium citrate, 0.5 g/L MgSO_4_7H_2_O, 0.5 mg/L CaCl_2_, 0.1 mg/mL ZnSO_4_, 0.2% (v/v) tyloxapol, 0.2% (v/v) ethanol and 0.01% (v/v) cholesterol (MMM-Ch). Prior to the antimycobacterial activity testing, *Mtb* cultures were pre-adapted in the different test conditions by washing 1 mL of culture suspension twice with c7H9, 7H9-Low BSA or MMM-Ch and resuspended in 10 mL of the same medium. Bacterial cultures were then incubated at 37 °C in 30 mL square bottles (Nalgene, USA) and allowed to stand for eight days. Compounds in powder form were dissolved in DMSO at 50 mM prior to assay. A modified resazurin based colorimetric assay was performed in a 96 well plate format in the three different media (c7H9, 7H9-Low BSA and MMM-Ch). Compounds were serially diluted column-wise to give a range of testing concentrations from 400 μM to 0.78 μM. Quality controls of drug only and bacteria only wells were also included with every plate. Rifampicin (1st line anti-TB drug) and DMSO controls were also included. Plates were inoculated with about 10^5^ CFUs of bacteria, sealed and incubated at 37 °C for one week (for c7H9 and 7H9-Low BSA media) or two weeks (for MMM-Ch medium). Following incubation, a solution containing 0.02% (w/v) resazurin dye (Sigma, USA) was added to all the wells and incubated for a further day. Minimum inhibition concentrations (MIC_90_) were calculated using visual determination of the lowest concentration of drug at which there was no change in the resazurin colour.

### 4.5 UV–visible spectroscopy

Interactions of compounds with untagged CYP121A1 were analyzed by UV–Visible spectroscopy. Protein fractions with Rz ratio of ∼2 were used for titrations. Compound stock solutions were made up to 30 mM in fresh DMSO. Titrations with clear colorless compound solutions were performed at 28 °C using either a single-beam Cary 60 UV–Visible spectrophotometer (Agilent, UK) or a dual-beam Cary 300 Bio UV–Visible spectrophotometer (Agilent, UK) recording between 240 and 800 nm. Temperature control was achieved using a Cary single or dual cell peltier accessory (Agilent, UK) and a Julabo AWC100 recirculating cooling bath (Fisher Scientific, UK). Titrations were performed in 1 cm path length quartz cuvettes (Starna Scientific, UK) using a matched pair when necessary. A solution of CYP121A1 (4–5 μM, Soret absorbance of 0.4–0.5 AU) was used per titration in sterile-filtered 100 mM HEPES, 100 mM KCl, 0.005% Tween-20, pH 7.8 at 28 °C (adjusted with KOH/HCl) to a final volume of 1 mL. Final DMSO concentrations were kept below 1% v/v. Prior to each titration, a baseline correction and zero was performed with buffer alone. Following this, CYP12A1 was added and the cuvette was incubated for 5 min to allow for temperature equilibration. A compound-free absorbance spectrum was recorded and then small volumes of compound (typically 0.05–0.5 μL) were introduced using a Hamilton syringe fitted with a syringe guide (Hamilton, USA). Following each compound addition an absorbance spectrum was recorded and this was repeated until no further spectral changes were observed. The data were baseline corrected and the difference plot (ΔAbs against wavelength) was generated by subtracting the compound-free absorbance spectrum from the spectra collected after each addition of compound. From this plot, the absorbance maximum ($A_{peak}$) and minimum ($A_{trough}$) were identified. Changes in absorbance due to compounds (ΔΔAbs) were calculated by subtracting the $\;A_{trough\;}$ value from the $A_{peak}$ value for each spectrum recorded. Values of ΔAbs were plotted against compound concentration (in μM) and the data were fitted using the Michaelis-Menten equation (see below) to derive the $K_{d}$ values (compound concentration at which $\Delta \Delta Abs=\;\frac{1}{2}A_{max}$). Analysis was performed in Microsoft Excel 2010 (Microsoft, USA) and OriginPro 9.1 (OriginLab, USA). Figures were generated using OriginPro 9.1 and CorelDRAW X7 (Corel, Canada).

$$\Delta \Delta Abs\;=\;\frac{A_{max}\times C}{K_{d}\times C}$$

In the Michaelis-Menten equation, ΔΔAbs is the observed change in absorbance for each compound addition (in AU), $A_{max}$ is the change in heme absorbance at apparent compound saturation (in AU), C is the compound concentration (in μM) and $K_{d}$ is the dissociation constant (in μM).

### 4.6 Isothermal titration calorimetry

ITC experiments to measure binding affinity of ligands to *Mtb* CYP121A1 were performed on a MicroCal Auto-iTC200 system (Malvern Instruments, UK) at 25 °C. Ligands were initially prepared as 25 mM stock solutions in DMSO‑*d*_6_ (and further diluted into DMSO‑*d*_6_ when necessary). Both ligands and CYP121A1 were diluted into identical buffer (50 mM Tris-HCl, 1 mM EDTA, pH 7.2) to generate mixtures containing 10% (v/v) DMSO‑*d*_6_, a final ligand concentration of either 0.5 or 2.5 mM (depending on ligand solubility) and a CYP121A1 concentration of 50 μM. Ligand titrations consisted of a small (0.2 μL) initial injection (that was discarded during data processing) followed by nineteen further injections (each of 2 μL) at 120 s intervals. Control titrations were also performed (adding ligand into buffer in the absence of protein) to measure any heats of dilution or buffer mismatch and were subtracted from ligand titrations during data processing. Titration isotherms were integrated to afford the enthalpy change of each injection and were plotted against the molar ratio of added ligand. Titrations were fitted using a one-site binding model using Origin Analysis Software by setting the stoichiometry (N) to one (for weak binding compounds) or allowing stoichiometry to vary (for more potent compounds).

### 4.7 Differential scanning fluorimetry

DSF was performed using a Bio-Rad CFX Connect system (Bio-Rad, UK), scanning from 25 °C to 95 °C in 0.5 °C increments each of 30 s duration. Samples were run in 96-well plates, with each well containing a final volume of 25 μL. Screening was conducted in 100 mM potassium phosphate pH 6.9, 2.5 × Sypro Orange, and with 5 μM Mtb CYP121A1 containing either 4% (v/v) DMSO‑*d*_6_ or 4% (v/v) of 25 mM stock solutions of ligands in DMSO‑*d*_6_ (final ligand concentration 1 mM). Experiments for samples giving a positive shift in protein melting temperature were repeated in triplicate under identical conditions and the collected data were averaged (over the four runs).

### 4.8 Liquid chromatography–mass spectrometry (LC–MS) activity assay

Untagged CYP121A1 was expressed and purified to homogeneity as previously reported [7,38]. *Escherichia coli* flavodoxin NADP + oxidoreductase (FLDR) was expressed and purified as previously reported [50,51]. *Spinacia oleracea* ferredoxin (FDX), *Leuconostoc mesenteroides* glucose-6-phosphate dehydrogenase (G6PDH), glucose-6-phosphate (G6P) and NADPH were purchased from Sigma–Aldrich (UK). Reaction mixtures comprising of 5 μM CYP121A1, 4.6 μM FLDR, 10 μM FDX, 2 units of G6PDH, 10 mM G6P and 2 mM NADPH were run in 50 mM Tris-base, 150 mM KCl, pH 7.6 at 28 °C in a final volume of 500 μL. Reactions were run in amber, silanized glass vials (Agilent, UK). Positive and negative controls were prepared containing either 100 μM cYY (Ambinter, France) or with none respectively. For experimental samples, either 100 μM of compound or 100 μM compound and 100 μM cYY were used. The amount of DMSO was normalized across all samples to 1.6% v/v. For the positive control and experimental samples, compound and/or cYY was introduced prior to CYP121A1 to allow for proper equilibration. Reactions were started with the introduction of a mastermix comprising G6PDH, G6P and NADPH. Samples were incubated at 28 °C and 220 rpm for 2 h before reactions were stopped by the addition of 1 mL DCM *via* glass pipette. Samples were then briefly vortexed and then centrifuged for 10 min (466×*g*), the resulting lower organic phase was extracted using a glass pipette. This process was repeated once more, the organic phases were pooled and dried overnight in a fumehood. Following this, samples were further dried for 20 min in an EZ-2 centrifugal evaporator (Genevac, UK) set to aqueous mode with the lamp off. Samples were resuspended in 150 μL ACN (Sigma-Aldrich, UK) supplemented with 0.1% formic acid (Sigma-Aldrich, UK). LC–MS analysis was performed on an Agilent 1290 uHPLC system coupled to an Agilent 6545XT LC-QTOF controlled by MassHunter 10 (Agilent, UK). Columns used were either an EclipsePlus C18 RRHD 1.8 μm 2.1 mm × 150 mm (Agilent, UK) or a BonusRP RRHD 1.8 μm 2.1 mm × 50 mm (Agilent, UK) eluting at 0.45 mL/min at 60 °C. Mobile phases were either water supplemented with 0.1% formic acid (A) or acetonitrile supplemented with 0.1% formic acid (B). A gradient of 3–40% B over 6 min was used for separation. Signal acquisition was achieved using MS1 mode scanning from 100 to 3000 Da at 4 Hz. Data were analyzed on MassHunter Qualitative 10 and Quantitative 10 (Agilent, UK).

### 4.9 UV–visible spectrophotometric competition assay

Compound stock solutions of both **14** and **61** at 100 mM and a 30 mM cYY stock solution were prepared in fresh DMSO for competition assays. A titration with cYY alone was performed as a control using a stock solution at 15 mM prepared in fresh DMSO. Assays were performed on a dual-beam Cary 300 UV–Visible spectrophotometer (Agilent, UK). Following introduction of 5 μM CYP121A1, 100 μM of either compound **14** or **61** was introduced in two equal additions and spectra recorded. The second spectrum taken was used as the starting point for a titration with cYY. Reverse competition assays were performed using a spectrum with 100 μM cYY present as the starting point for a titration with compound **14** or **61**. A 100 mM cYY stock solution and 30 mM compound stock solutions were prepared in fresh DMSO for the reverse competition assay. Final DMSO concentrations were kept below 1% v/v. Titrations were performed and data processed as previously described. Data were fit to the Hill equation to derive the $K_{d}$ values (compound concentration at which $\Delta \Delta Abs=\;\frac{1}{2}A_{max}$), as shown below:

$$\Delta \Delta Abs=\;\frac{\left(A_{max}*C^{n}\right)}{\left({K_{d}}^{n}+C^{n}\right)}$$

In the Hill equation, ΔΔAbs refers to the observed absorption difference at each compound addition (in AU), $A_{max}$ is the change in heme absorbance at apparent compound saturation (in AU), C is the compound concentration used (in μM), n is the number of cooperative binding sites and $K_{d}$ is the dissociation constant (in μM).

## Dedication

One of the authors, Professor Chris Abell, died suddenly during the preparation of this manuscript. His fellow authors wish to dedicate this paper to his memory.

## Author contributions

Chemistry (MF, BC, MEK and SC), expression and purification of CYP121A1 (IRS, MF, MMK), X-ray crystallography (IRS, RBT, CWL, DL), antimycobacterial activity assays (DE, LPSdC), UV-vis spectroscopy (IRS), DSF and ITC (MF), LC-MS activity assay and competition assay (IRS, RTB), manuscript preparation (MF, IRS, DE, AGC), concpts and experimental design (MF, IRS, AGC, KJM), project management and supervision (AGC, KJM, AWM, DL, CA).

## Declaration of competing interest

The authors declare that they have no known competing financial interests or personal relationships that could have appeared to influence the work reported in this paper.
