# Supplementary material for: A new strategy for hit generation: Novel in cellulo active inhibitors of CYP121A1 from Mycobacterium tuberculosis via a combined X-ray crystallographic and phenotypic screening approach (XP screen)
Source: Eur J Med Chem. 2022 Feb 15;230:114105. doi: 10.1016/j.ejmech.2022.114105 (PMC8856928; doi:10.1016/j.ejmech.2022.114105)
Supplement: Multimedia component 1 [file mmc1.pdf]

Supporting information

A new strategy for hit generation: novel *in cellulo* active inhibitors of CYP121A1 from *Mycobacterium tuberculosis* via a combined X-ray crystallographic and phenotypic screening approach (XP Screen)

Martyn Frederickson,[†] Irwin R. Selvam,[‡] Dimitrios Evangelopoulos,[§,‖] Kirsty J. McLean,[‡,¤] Mona M. Katariya,[†] Richard B. Tunnicliffe,[‡] Bethany Campbell,[†] Madeline E. Kavanagh,[†,☨] Sitthivut Charoensutthivarakul,[†,⁋] Richard T. Blankley,[Ω] Colin W. Levy,[#] Luiz Pedro S. de Carvalho,[§] David Leys,[‡] Andrew W. Munro,[‡,*] Anthony G. Coyne,[†,*] and Chris Abell[†]

† Yusuf Hamied Department of Chemistry, University of Cambridge, Lensfield Road, Cambridge, CB2 1EW, United Kingdom

‡ Department of Chemistry, Manchester Institute of Biotechnology, University of Manchester, 131 Princess Street, Manchester, M1 7DN, United Kingdom

§ Mycobacterium Metabolism and Antibiotic Research Laboratory, Francis Crick Institute, 1 Midland Road, London, NW1 1AT, United Kingdom

Manchester Protein Structure Facility (MPSF), Manchester Institute of Biotechnology, University of Manchester, Manchester, M1 7DN, United Kingdom

Ω Agilent Technologies U.K. Ltd, 5500 Lakeside, Cheadle Royal, Cheshire, SK8 3GR, United Kingdom

‖ Current address: Department of Microbial Diseases, Eastman Dental Institute, University College London, Royal Free Campus, Rowland Hill Street, London, NW3 2PF, United Kingdom

¤ Current address: Department of Biological and Geographical Sciences, University of Huddersfield, Queensgate, Huddersfield, HD1 3DH, United Kingdom

☨ Current address: Department of Chemistry, The Skaggs Institute for Chemical Biology, The Scripps Research Institute, La Jolla, CA, USA.

⁋ Current address: School of Bioinnovation and Bio-based Product Intelligence, Faculty of Science, Mahidol University, Bangkok, 10400, Thailand

* E-mail: agc40@cam.ac.uk (A.G.C.) and andrew.munro@manchester.ac.uk (A.W.M.)

Table of Contents

LC-MS traces (for compounds with MIC_{90}): S3

X-ray crystallography: S8

UV-Visible spectroscopy: S8

Isothermal Titration Calorimetry (ITC): S11

Differential scanning fluorimetry: S13

UV-Visible spectroscopy competition assay: S15

LC-MS traces (for compounds with MIC_{90}):

Compound **14**

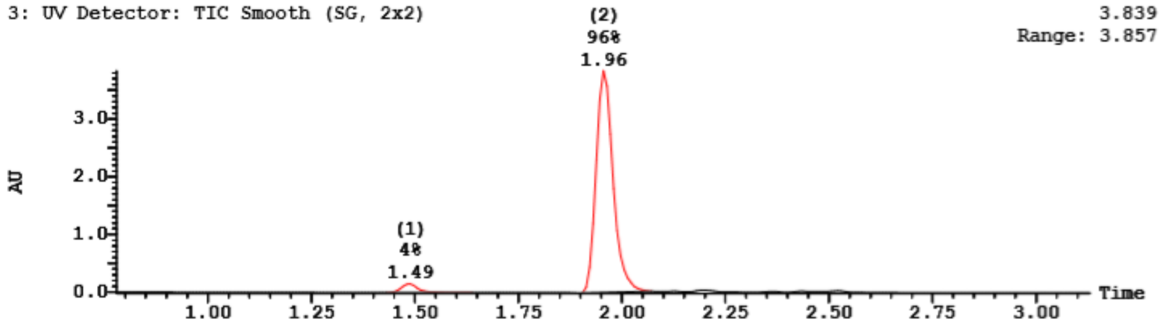

Peak Number	Time	AreaAbs	Area %Total	Height	Mass Found
1	1.49	6808	3.58	149194	
2	1.96	183558	96.42	3843988	

Compound **15**

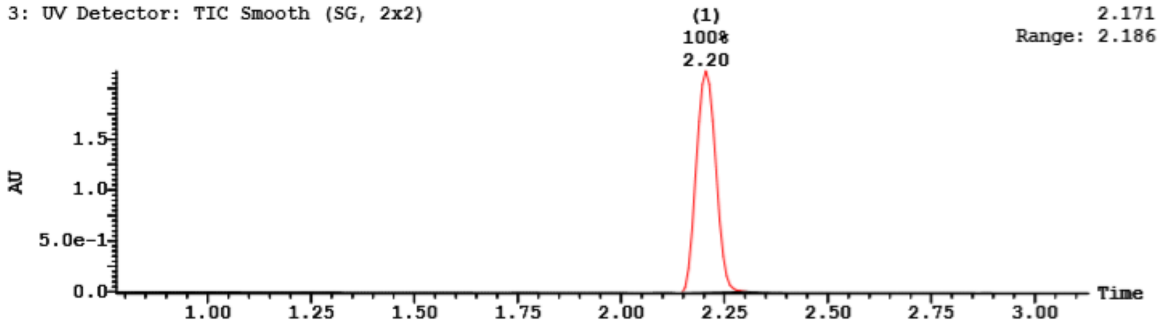

Peak Number	Time	AreaAbs	Area %Total	Height	Mass Found
1	2.20	118751	100.00	2177532	

Compound **23**

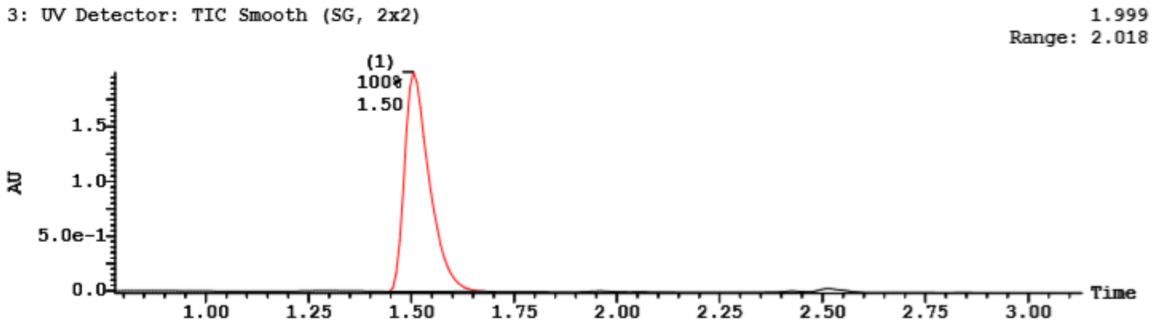

Peak Number	Time	AreaAbs	Area %Total	Height	Mass Found
1	1.50	140026	100.00	2004428	

Compound **30**

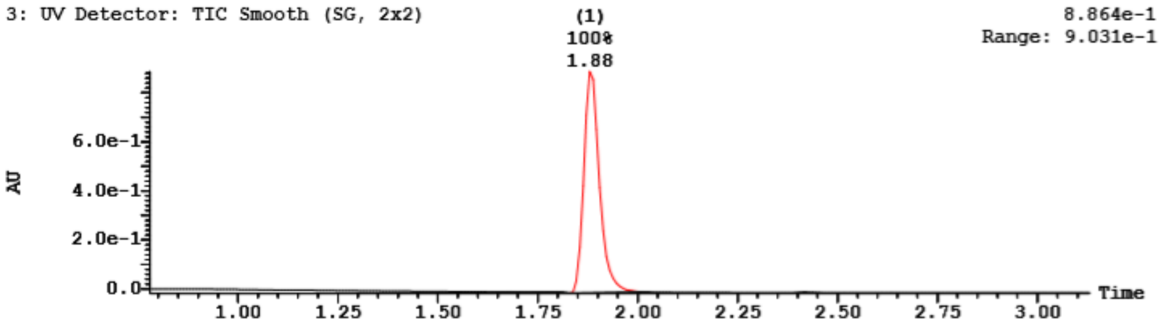

Peak Number	Time	AreaAbs	Area %Total	Height	Mass Found
1	1.88	40795	100.00	901434	

Compound **31**

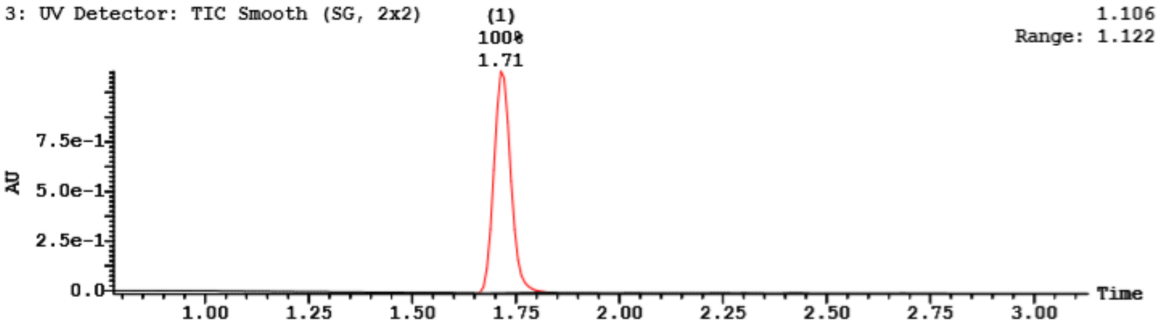

Peak Number	Time	AreaAbs	Area %Total	Height	Mass Found
1	1.71	52909	100.00	1117716	

Compound **32**

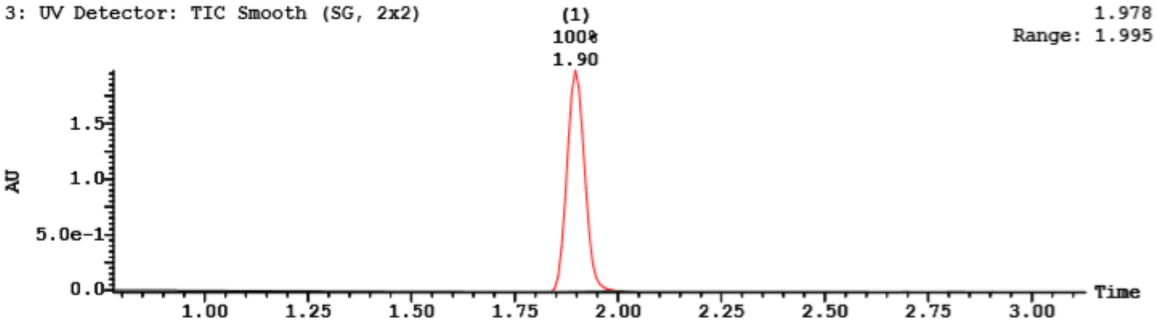

Peak Number	Time	AreaAbs	Area %Total	Height	Mass Found
1	1.90	98202	100.00	1989084	

Compound **34**

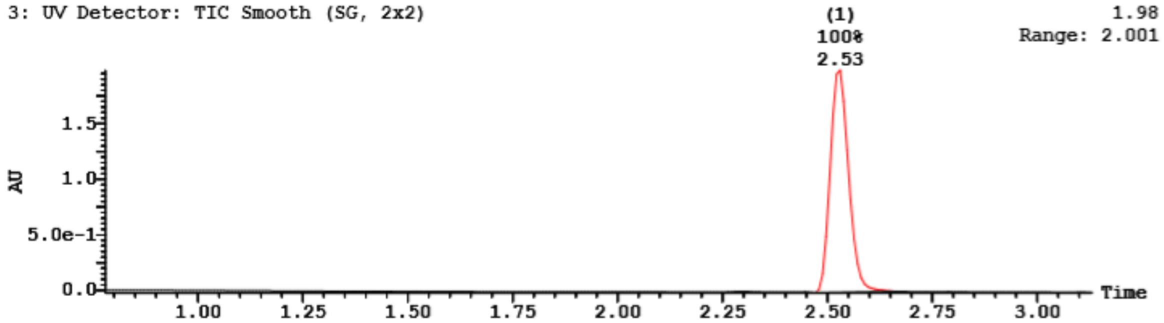

Peak Number	Time	AreaAbs	Area %Total	Height	Mass Found
1	2.53	101394	100.00	1996690	

Compound **51**

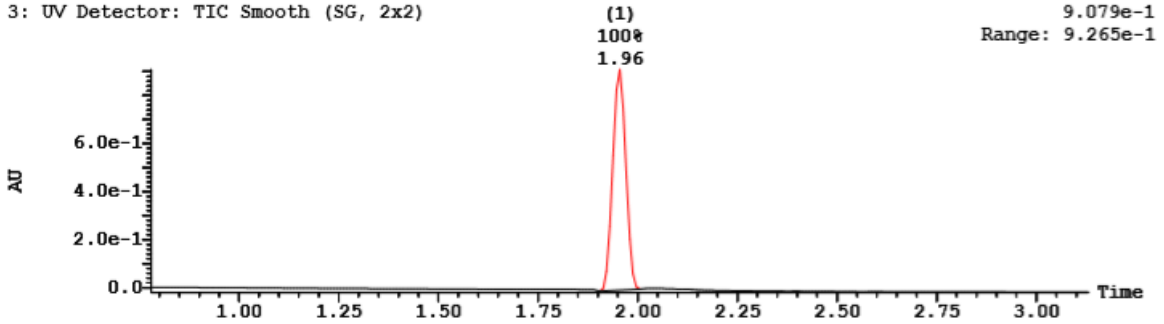

Peak Number	Time	AreaAbs	Area %Total	Height	Mass Found
1	1.96	34851	100.00	917218	

Compound **53**

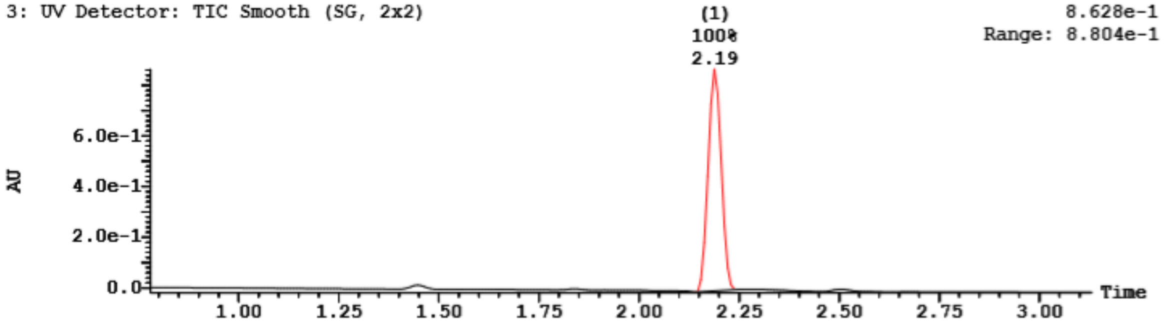

Peak Number	Time	AreaAbs	Area %Total	Height	Mass Found
1	2.19	33073	100.00	874809	

Compound **57**

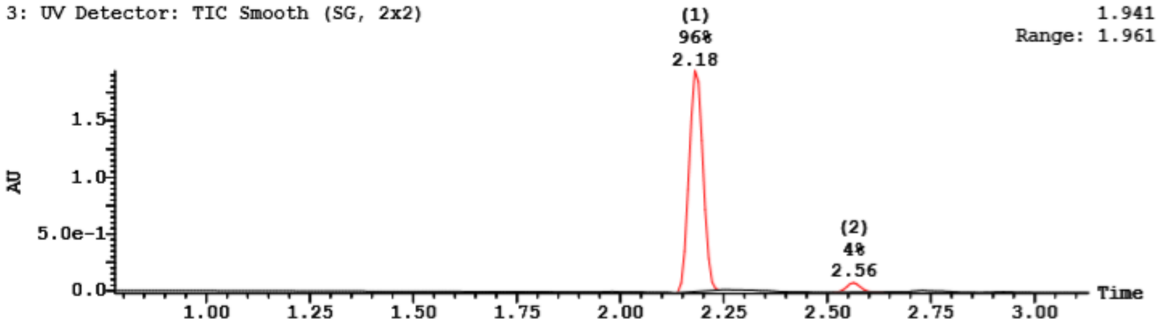

Peak Number	Time	AreaAbs	Area %Total	Height	Mass Found
1	2.18	75735	95.96	1946896	
2	2.56	3187	4.04	82707	

Compound **58**

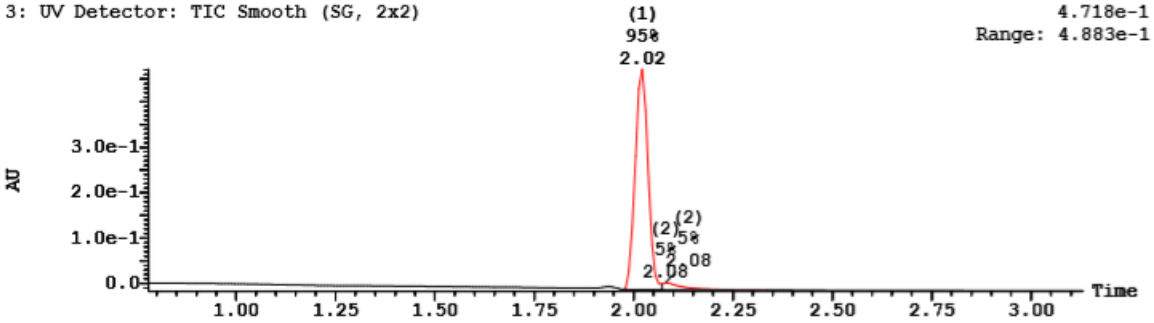

Peak Number	Time	AreaAbs	Area %Total	Height	Mass Found
1	2.02	18205	95.28	485562	
2	2.08	902	4.72	14896	

Compound **60**

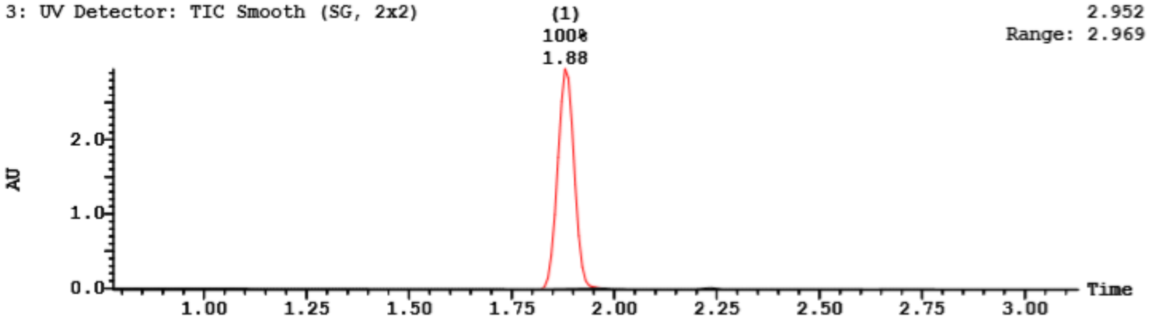

Peak Number	Time	AreaAbs	Area %Total	Height	Mass Found
1	1.88	137546	100.00	2957601	

Compound **61**

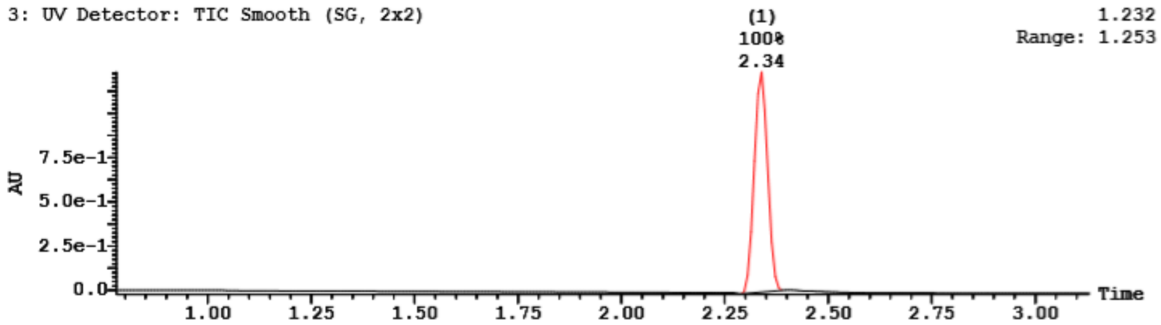

Peak Number	Time	AreaAbs	Area %Total	Height	Mass Found
1	2.34	46589	100.00	1242867	

Compound **63**

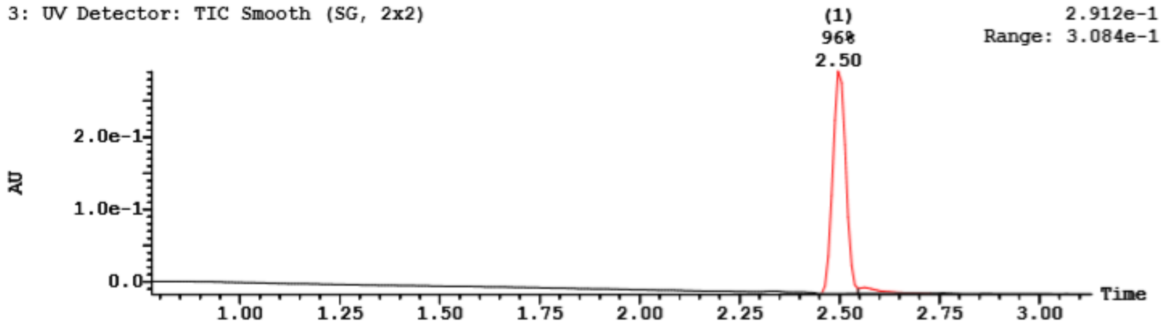

Peak Number	Time	AreaAbs	Area %Total	Height	Mass Found
1	2.50	11678	95.82	307640	
2	2.56	510	4.18	8340	

Compound **64**

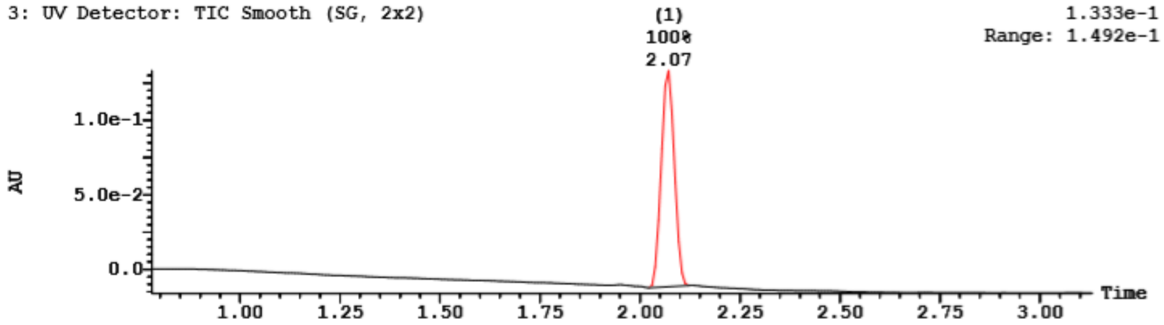

Peak Number	Time	AreaAbs	Area %Total	Height	Mass Found
1	2.07	5503	100.00	144997	

X-ray crystallography data collection and refinement statistics:

**Compound**	**10**	**14**	**21**
**PDB ID**	**7NQM**	**7NQN**	**7NQO**
**Data Collection**			
Wavelength (Å)	0.92	0.98	0.92
Resolution range (Å)	64.3–1.60 (1·66–1.60)	65.5–1.60 (1·66–1.60)	53.6–1.60 (1.66–1.60)
Space group	$P6_5\,2\,2$	$P6_5\,2\,2$	$P6_5\,2\,2$
Cell dimensions			
a, b, c (Å)	76.6, 76.63, 262	78.1, 78.1, 264	77.9, 77.9, 264
α, β, γ (°)	90, 90, 120	90, 90, 120	90, 90, 120
Total reflections	1141001 (109898)	1170918 (112447)	1176974 (112635)
Unique reflections	60413 (5826)	63068 (6119)	63696 (6252)
Multiplicity	18.9 (18.9)	18.6 (18.4)	18.5 (18.0)
Completeness (%)	98.9 (97.9)	98.8 (97.8)	100 (100)
Mean $I/\sigma(I)$	15.3 (1.15)	15.5 (0.73)	7.29 (0.40)
Wilson *B*-factor	18.3	22.6	19.6
R_{merge}	0.09 (0.83)	0.08 (0.98)	0.17 (0.73)
R_{meas}	0.09 (0.85)	0.08 (1.01)	0.17 (0.75)
R_{pim}	0.02 (0.19)	0.02 (0.23)	0.04 (0.17)
$CC_{1/2}$	1.00 (0.92)	0.97 (0.89)	1.00 (0.91)
CC*	1.00 (0.98)	0.99 (0.97)	1.00 (0.98)
**Refinement**			
Resolution range (Å)	64.3–1.6	65.5–1.6	53.6–1.6
Reflections used in refinement	60407 (5825)	63054 (6117)	63691 (6251)
Reflections used for R-free	1993 (192)	1997 (194)	1999 (196)
R_{work}	0.16 (0.26)	0.18 (0.29)	0.17 (0.24)
R_{free}	0.19 (0.28)	0.21 (0.35)	0.20 (0.26)

CC_{work}	0.97 (0.91)	0.96 (0.88)	0.97 (0.92)
CC_{free}	0.99 (0.86)	0.95 (0.81)	0.96 (0.88)
Number of non-hydrogen atoms	4168	3980	4118
Protein	3287	3203	3267
Ligand/Ion	314	217	223
Water	675	636	709
Number of residues			
Protein	395	395	394
Ligand/Ion	1 heme, 2 **10**, 4 MES, 16 SO_4, 2 Cl^-	1 heme, 1 **14**, 2 MES, 9 SO_4, 6 Cl^-	1 heme, 1 **21**, 2 MES, 1 DMSO, 9 SO_4, 2 Cl^-
RMS deviations			
Bond length (Å)	0.01	0.02	0.02
Bond angle (°)	1.05	1.75	1.47
Ramachandran favored (%)	99.2	99.5	99.2
Ramachandran allowed (%)	0·76	0·51	0·77
Ramachandran outliers (%)	0.00	0.00	0.00
Rotamer outliers (%)	1.36	1.98	0.82
Clashscore	6.77	5.04	5.47
B-factors ($Å^2$)			
Average	22.4	26.4	23.2
Protein	19.6	24.3	20.8
Ligand/Ion	33.6	34.0	31.0
Water	32.5	35.6	33.0

Table S1: Data collection and refinement statistics for 7NQM, 7NQN and 7NQO in complex with lead compounds **10**, **14** and **21** respectively. Statistics for the highest resolution shell are shown in parentheses. Prepared with Phenix 1.19-4092.

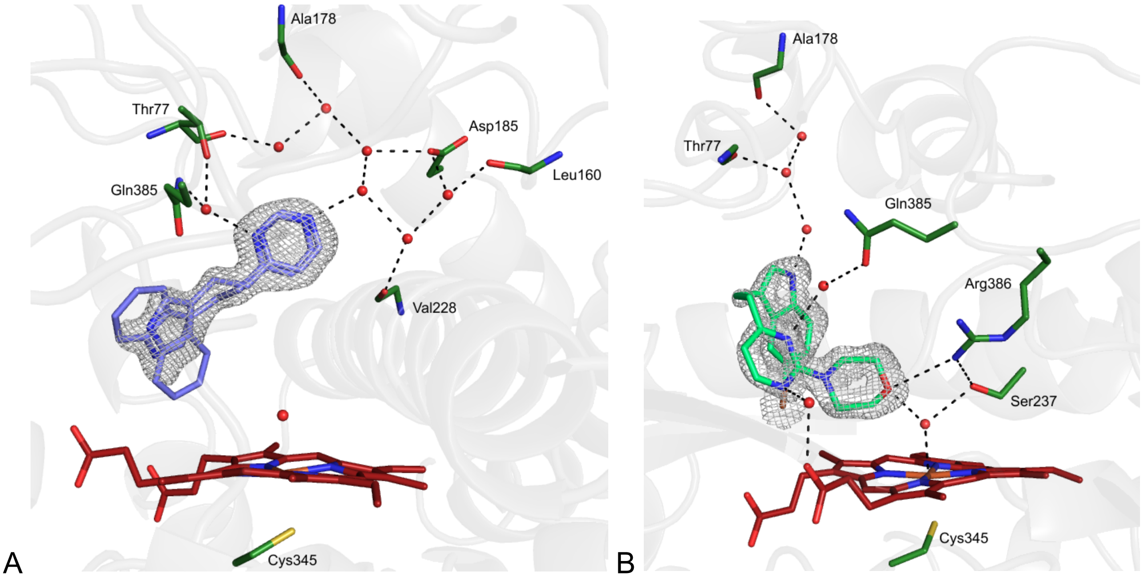

Figure S1: X-ray crystallographic structures. a) CYP121A1 in complex with **10** (7NQM) showing two possible alternative ligand binding modes, based upon the incomplete density for the 3-indolyl moiety of the ligand. b) CYP121A1 in complex with **21** (7NQO). The ligand assumes a highly folded conformation. 2Fo-Fc maps are shown as a fine mesh contoured to one sigma. Possible hydrogen bonding networks are shown as black dashes, all distances are <3.2 Å.

UV-Visible spectroscopy (UV-Vis):

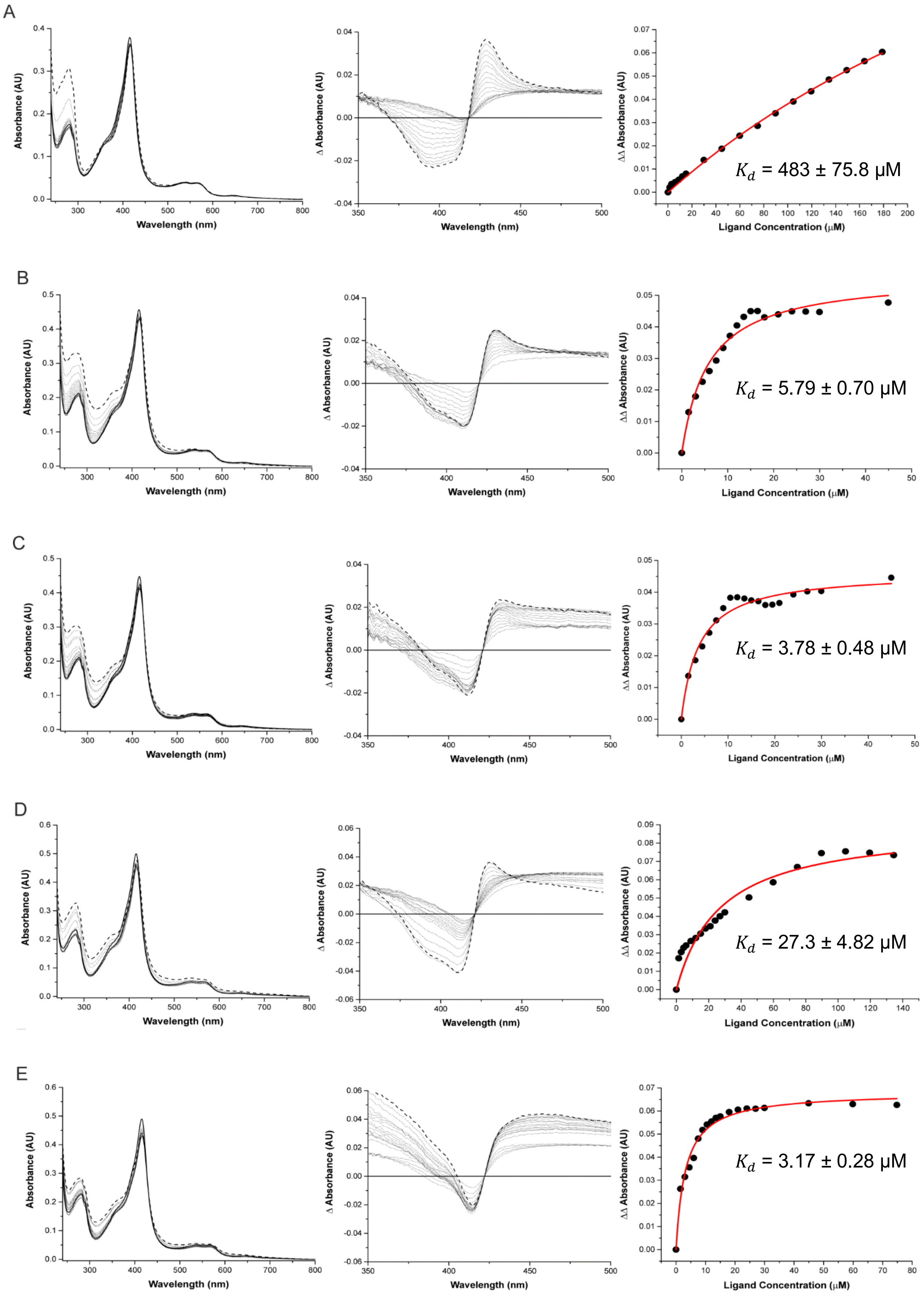

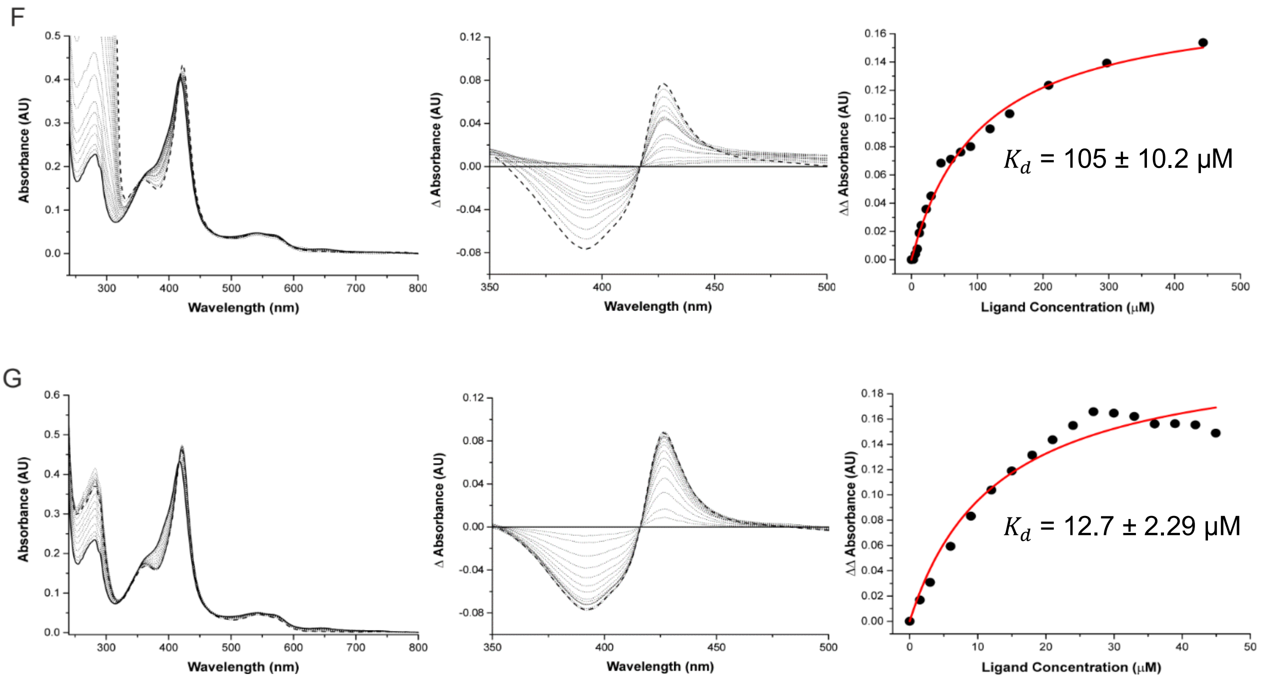

Figure S2: UV-Vis spectra for compounds **7** (A), **8** (B), **9** (C), **41** (D), **42** (E), **43** (F) and **44** (G). Left: Titrations of CYP121A1 with compounds (compound-free spectra represented by thick solid lines, intermediate spectra by thin dotted lines and final spectra by thick dashed lines); Middle: Difference plots generated from titrations of CYP121A1 with compounds (compound-free spectra represented by the *x*-axis, intermediate spectra by thin dotted lines and final spectra by thick dashed lines); Right: Concentration-dependent changes in heme absorbance of CYP121A1 with compounds (solid dots represent changes in absorbance after each addition of compound, red curves were fitted using the Michaelis-Menten equation).

Isothermal Titration Calorimetry (ITC) :

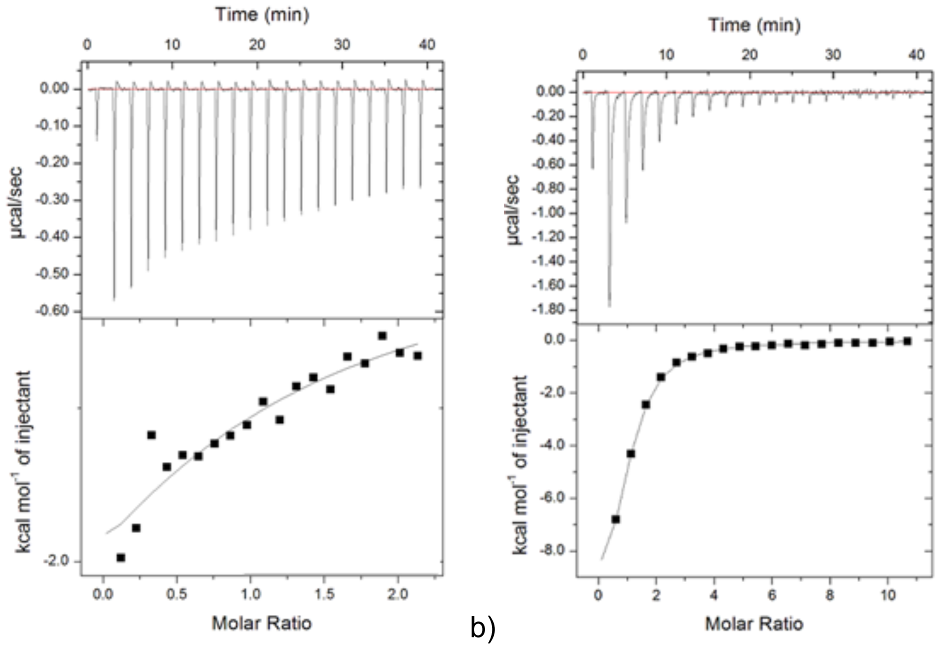

a) b)

Figure S3: ITC thermograms for: a) **14** and b) **45**.

Differential scanning fluorimetry :

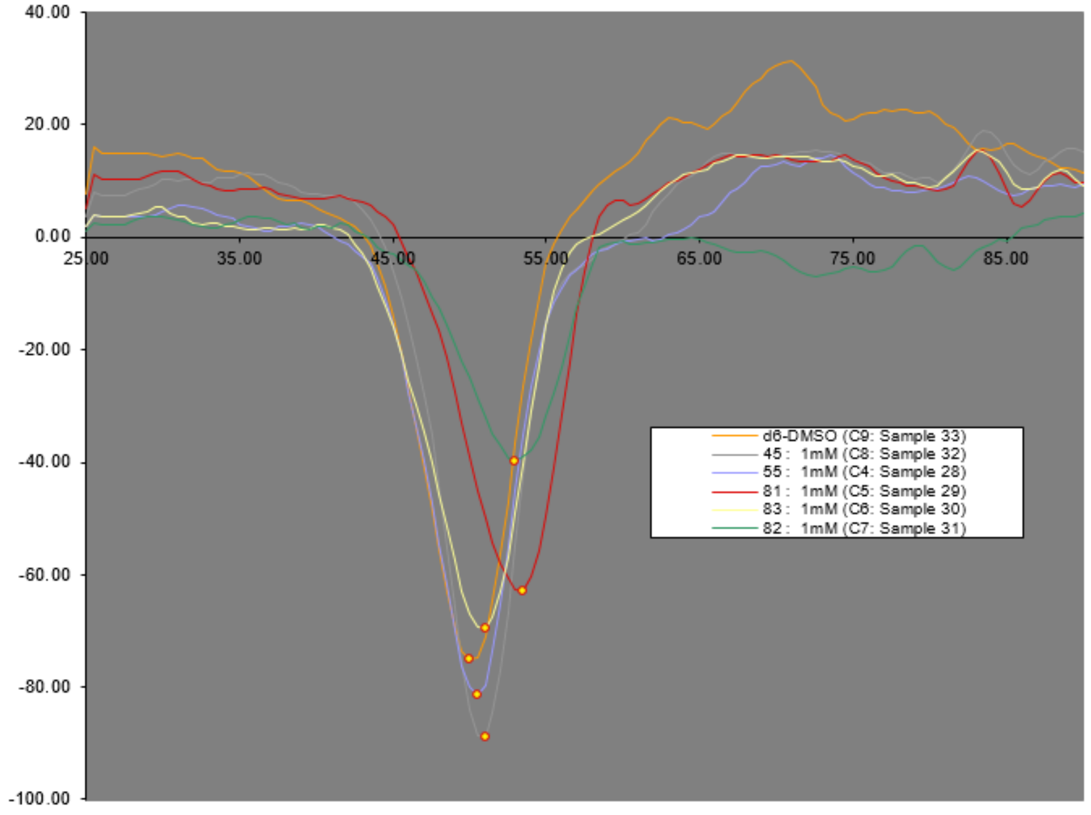

a)

b)

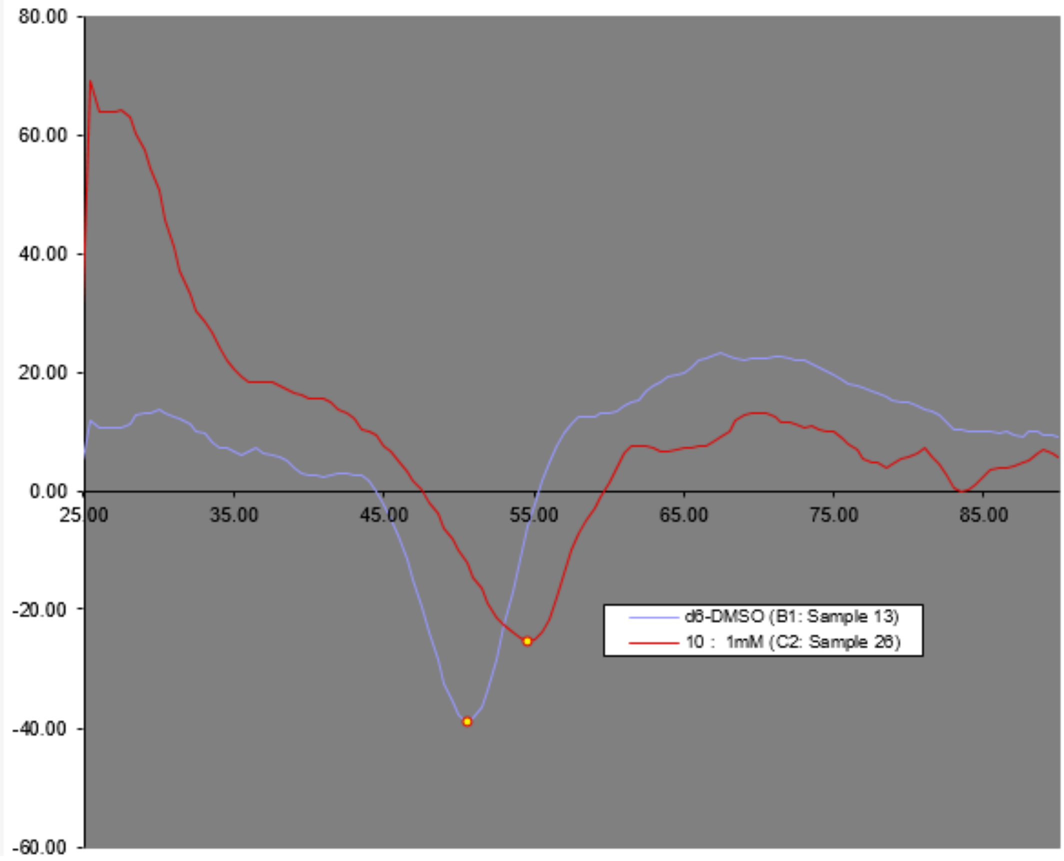

Figure S4: Differential scanning fluorimetry melting curves for : a) d_6-DMSO, **45**, **55**, **81**, **82** and b) **83** b) d_6-DMSO and **10**.

UV–Visible spectroscopy competition assay:

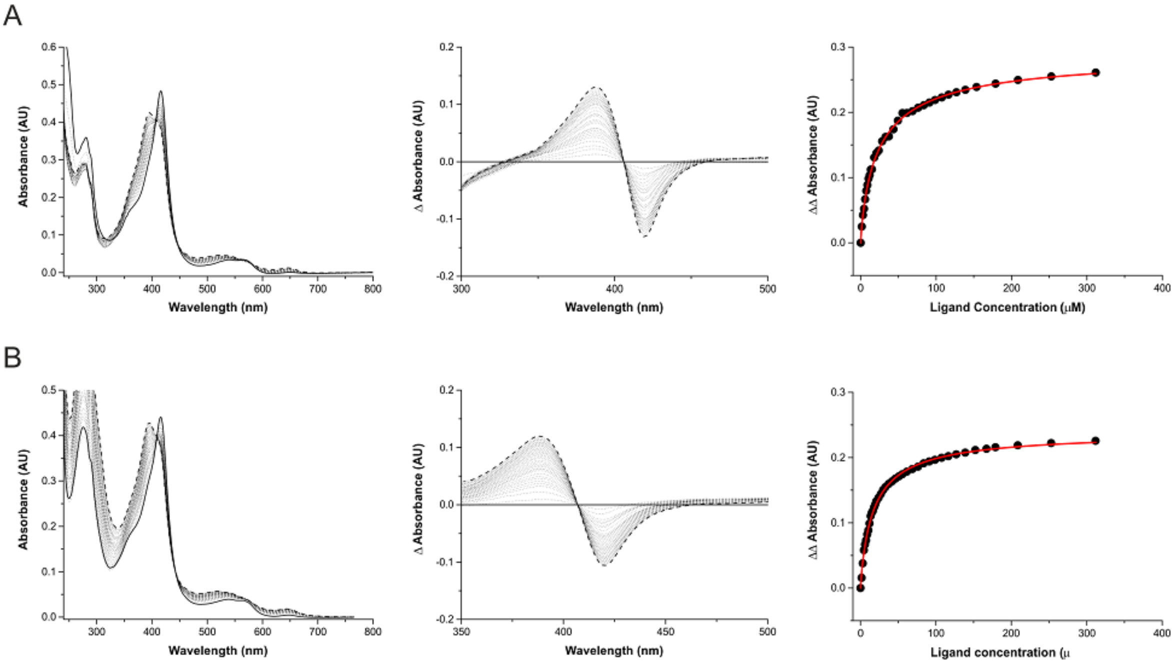

Figure S5: UV-Visible spectra for titrations with cYY (30 mM) in the presence of **14** (100 mM) (A) or **61** (100 mM) (B). Left: Titrations with CYP121A1 (**14** or **61**-bound spectra represented by thick solid lines, intermediate spectra with cYY by thin dotted lines and final spectra with cYY by thick dashed lines); Middle: Difference plots generated from the titrations (**14** or **61**-bound spectra represented by the *x*-axis, intermediate spectra by thin dotted lines and final spectra by thick dashed lines); Right: Concentration-dependent changes in heme absorbance of CYP121A1 with cYY (solid dots represent changes in absorbance after each addition of cYY, red curves were fitted using the Hill equation).
